# Synthesis of Benzofuran Derivatives via a DMAP-Mediated Tandem Cyclization Reaction Involving *ortho*-Hydroxy α-Aminosulfones

**DOI:** 10.3390/molecules29163725

**Published:** 2024-08-06

**Authors:** Rong-Rong Zhu, Xi-Qiang Hou, Da-Ming Du

**Affiliations:** 1 School of Chemistry and Chemical Engineering, Beijing Institute of Technology, No. 5 Zhongguancun South Street, Beijing 100081, China; 3120221250@bit.edu.cn (R.-R.Z.);; 2 Key Laboratory of Medicinal Molecule Science and Pharmaceutical Technology, Ministry of Industry and Information Technology, No. 5 Zhongguancun South Street, Beijing 100081, China

**Keywords:** benzofuran, *ortho*-hydroxy α-amino sulfones, tandem cyclization reaction, spirocyclic compounds, Mannich reaction

## Abstract

An efficient cascade cyclization strategy was developed to synthesize aminobenzofuran spiroindanone and spirobarbituric acid derivatives utilizing 2-bromo-1,3-indandione, 5-bromo-1,3-dimethylbarbituric acid, and *ortho*-hydroxy α-aminosulfones as substrates. Under the optimized reaction conditions, the corresponding products were obtained with high efficiency, exceeding 95% and 85% yields for the respective derivatives. This protocol demonstrates exceptional substrate versatility and robust scalability up to the Gram scale, establishing a stable platform for the synthesis of 3-aminobenzofuran derivative. The successful synthesis paves the way for further biological evaluations with potential implications in scientific research.

## 1. Introduction

Benzofuran, a crucial fused heterocycle, exhibits diverse pharmacological properties, including antitumor, anti-inflammatory, antiviral, and anti-osteoporotic activities [1,2,3,4,5,6,7,8]. For instance, compound **A** can stimulate bone formation, accelerate bone turnover, augment the proportion of osteoblasts, and effectively prevent and treat glucocorticoid-induced osteoporosis by upregulating BMP-2 expression [9]. Compound **B** demonstrates exceptional anticancer activity against breast cancer cells [10,11].

**Figure 1:**
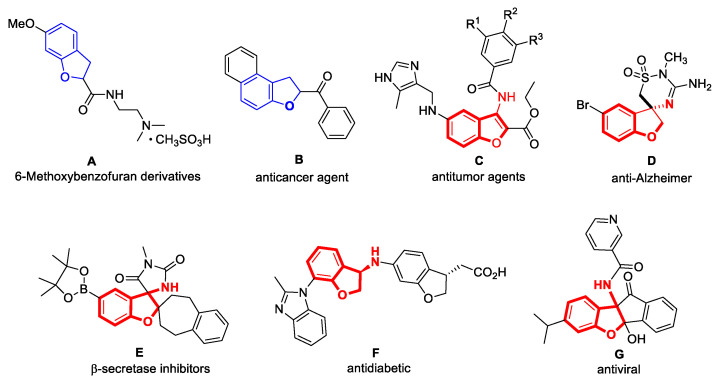
Compounds with benzofuran and 3-aminobenzofuran as core scaffolds.

The 3-aminobenzofuran scaffold represents a significant subclass within the diverse family of benzofuran-based compounds, occupying a prominent position at the forefront of medical treatment advancements and innovations [12,13,14]. Its unique structural features contribute to its promising potential in developing novel therapeutic agents [15,16,17] (Figure 1). Compound **C** exhibits potent antitumor properties by inducing cell cycle arrest and promoting apoptosis within cellular environments [18]. Compound **D** stands as a crucial constituent in therapeutic agents aimed at alleviating the symptoms of Alzheimer’s disease [19].

The 3-amino substitution enhances the biological activities of benzofuran derivatives, prompting research into innovative synthetic strategies to facilitate their clinical applications [20,21,22,23,24]. Recently, Helgren et al. introduced a microwave-facilitated, unprotected group synthesis of 3-amino-2,3-dihydrobenzofurans via chalcone precursors. This synthesis uses acid-catalyzed aldol condensation, reduction, and epoxidation reactions, facilitating C2, C3, and A-ring modifications [25]. Wang and colleagues have developed a method for the synthesis of enantiomerically enriched 2,3-dihydrobenzofurans bearing quaternary carbon stereocenters in moderate to excellent yields (31–98%) through the copper/BOX complex-catalyzed reaction of 2-imino-substituted phenols with aryl diazoacetates. This reaction employs readily accessible catalysts and mild reaction conditions, demonstrating broad functional group tolerance, single diastereoisomer formation, and excellent enantioselectivity (88–97% ee). The reaction proceeds via a distinctive mechanism in which the copper catalyst exerts two distinct functions. Initially, it reacts with the diazo compound to generate a metallacarbene. Subsequently, it acts as a Lewis acid to activate the imine following the formation of an oxonium ylide [26]. Panday and coworkers swiftly synthesized 3-amino-2-arylbenzofurans at room temperature via Cs_2_CO_3_-catalyzed route, advancing to Cu(II)-catalyzed *N*-arylation [27]. Abtahi and coworkers achieved one-pot 3-aminobenzofuran synthesis using CuI in a deep eutectic solvent [28]. The current synthetic methodologies for the construction of 3-aminobenzofuran frameworks, which encompass radical cyclization, coupling-cyclization, and Lewis acid/transition metal catalysis, are frequently impeded by complex purification procedures and rigorous reaction conditions. This hinders their practical applicability.

**Scheme 1:**
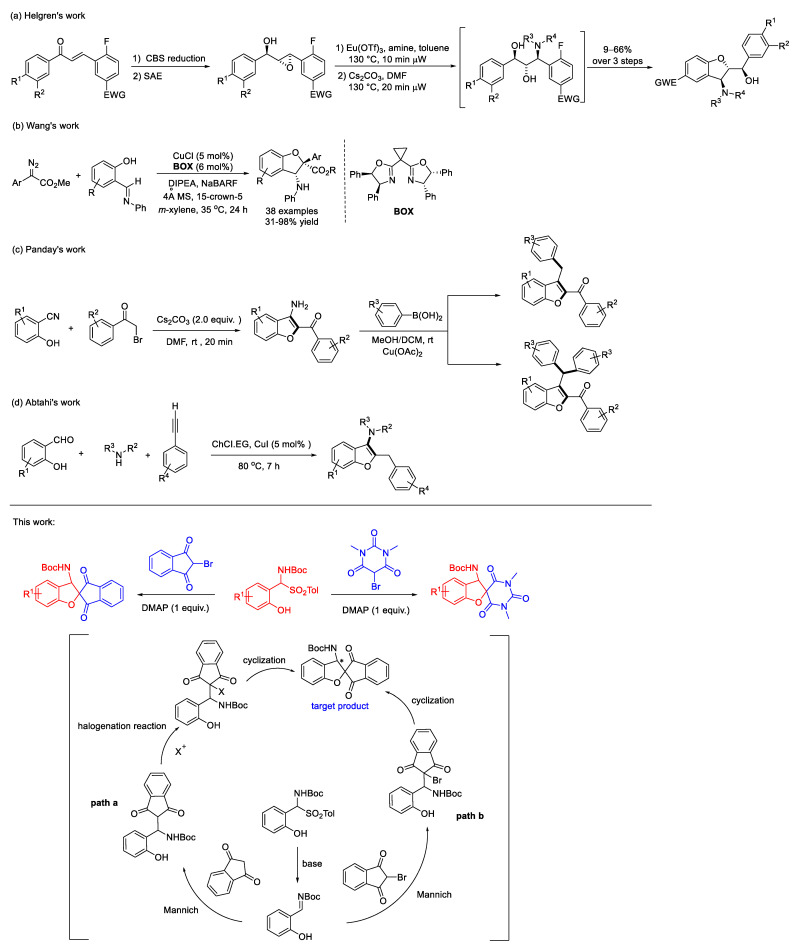
Synthesis of 3-aminobenzofuran derivatives and our contributions.

Meanwhile, indanone and barbituric acid are prevalent in natural products and bioactive pharmaceutical molecules [29,30,31,32,33]. The structures of 1,3-indanedione and 1,3-dimethylbarbituric acid, which serve as pivotal pharmacophore scaffolds, have garnered extensive research attention. The conventional utilization of 1,3-indandione [34] and 1,3-dimethylbarbituric acid [35,36] structures has been primarily focused on their benzylidene unsaturation moiety, offering a rich platform for chemical modifications and diverse functionalization. However, there is a scarcity of reports detailing their involvement in tandem cyclization reactions, highlighting an underexplored area with significant potential for further investigation and development [37,38,39,40]. The combined pharmacophore strategy allows us to harness the potential of 1,3-indandione and 1,3-dimethylbarbituric acid to engage in tandem reactions with *ortho*-hydroxy α-amino sulfones, thereby constructing a series of target products featuring an aminobenzofuranospiroindanone and spirobarbiturate core skeleton. While devising a synthetic route, several unsuccessful attempts were made to utilize halogen-substituted *N*-succinimides as the primary halogen source (Scheme 1, path a). We then strategically shifted our focus to bromine-substituted 1,3-indandione as the substrate (Scheme 1, path b), a pivotal decision that ultimately led to the synthesis of a product structure with profound implications for the development of novel therapeutic agents.

## 2. Results and Discussion

### 2.1. Optimization of Reaction Conditions

To ascertain the optimal synthetic conditions for the benzofuran-spiroindenone derivative, we employed *ortho*-hydroxy α-aminosulfone **1a** and 2-bromo-1,3-indandione **2a** as the starting materials and stirred them in dichloromethane solvent at ambient temperature. This approach facilitated the identification of the most favorable experimental parameters. A preliminary investigation was conducted to assess the impact of various bases on the reaction efficiency (Table 1, entries 1–9). When using NaHCO₃ as the base, the desired product, namely **3aa**, was obtained in 42% yield after 20 h reaction (Table 1, entry 1). Subsequently, Na_2_CO_3_, K_2_CO_3_, and Cs_2_CO_3_ were subjected to screening. The results demonstrated that the effects of Na_2_CO_3_ and K_2_CO_3_ were comparable (Table 1, entries 2 and 3). However, when Cs_2_CO_3_ was utilized, there was a considerable reduction in the yield (41%) (Table 1, entry 4). A selection of organic bases (Table 1, entries 5–9) was subjected to preliminary screening. When Et_3_N was used, there was no discernible improvement in the reaction yield, which remained at approximately 45% (Table 1, entry 5). Additionally, the other three organic bases, DABCO, DBU, and TMG, were also subjected to preliminary screening. When using these bases in the template reaction, there was no discernible alteration in the actual reaction outcome, with the yield fluctuating around 30% (Table 1, entries 6–8). It is noteworthy that when the organic base DMAP was subjected to evaluation (Table 1, entry 9), the reaction outcome exhibited a notable improvement, resulting in a yield increase of 70%.

Following the identification of the optimal base, a screening of solvents for the reaction was conducted, with the solvents listed in Table 1 (entries 10–15). When dichloroethane (DCE) was employed as the solvent (Table 1, entry 10), the yield was further augmented to 85%. In contrast, when the aromatic hydrocarbon solvent toluene was used, the yield of the reaction was not significantly enhanced (Table 1, 11). The large polar solvents, CH₃CN and EtOH, were also tested (Table 1, 12, and 14), but no improvement was observed in the reaction. Apart from exploring protic solvents, we also evaluated aprotic solvents, including THF (Table 1, entry 13) and MTBE (Table 1, entry 15). Nevertheless, the outcomes did not meet our initial expectations of significant enhancement. Through method optimization, it was established that the most favorable reaction conditions entailed utilizing DMAP as the base and DCE as the solvent, leading to a high yield of up to 85% for the desired product.

### 2.2. Substrate Scope

**Scheme 2:**
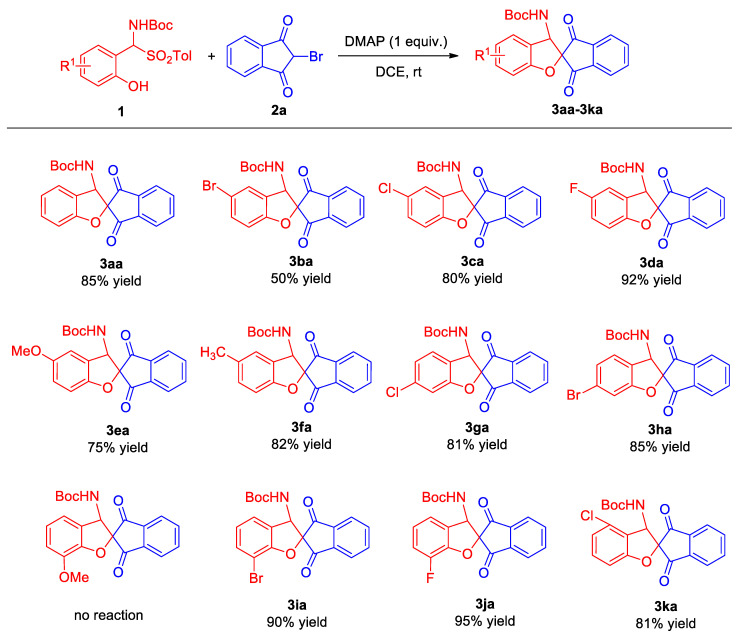
Substrate expansion for the tandem cyclization reaction of *ortho*-hydroxy α-aminosulfone and 2-bromo-1,3-indandione. The reaction was conducted under the following conditions: *ortho*-hydroxy α-aminosulfone **1** (0.15 mmol), 2-bromo-1,3-indandione **2** (0.1 mmol), and DMAP (1 equiv.) in DCE (1.0 mL) was stirred for 20 h at room temperature, followed by silica gel column chromatography for the separation of the products.

Once the optimal reaction conditions had been determined, a comprehensive investigation was conducted into the substrate scope of this reaction (Scheme 2). The examination of the 5-position substituents on *ortho*-hydroxy α-aminosulfone revealed that substitution with chlorine or fluorine at this position facilitated the synthesis of the target products **3ca** and **3da** with good to excellent yields. Unexpectedly, the substitution of the 5-position with bromine led to the synthesis of the desired product **3ba**, albeit with a modest yield of merely 50%. It is noteworthy that the strategic substitution of the 5-position in *ortho*-hydroxy α-aminosulfone with electron-rich groups, particularly methoxy and methyl groups, resulted in the successful synthesis of target compounds **3ea** and **3fa**, which exhibited good yields. Moreover, a comprehensive examination was undertaken to ascertain the impact of substituents at the 4-position on the physicochemical properties of these α-aminosulfones. The substitution of the 4-position with halogen atoms, such as chlorine or bromine, resulted in the successful acquisition of target products **3ga** and **3ha** with satisfactory yields. Subsequently, the substitution pattern at the 3-position of *ortho*-hydroxy α-aminosulfone was investigated. When the 3-position was substituted with the electron-donating methoxy group, the reaction failed to yield the desired target product. However, when the 3-position was substituted with halogen bromine or fluorine, the target products **3ia** and **3ja** were obtained with excellent yields of 90% and 95%, respectively. This highlights the superior reactivity of halogen-substituted *ortho*-hydroxy α-aminosulfones in comparison to those bearing electron-donating groups. Lastly, the reactivity of the 6-chloro-substituted *ortho*-hydroxy α-aminosulfone was evaluated, revealing a smooth reaction course that led to the formation of product **3ka** with a good yield of 81%. These findings underscore the intricate interplay between substitution patterns and reactivity patterns in the synthesis of functionalized *ortho*-hydroxy α-aminosulfones.

Building upon the successful outcomes of the preceding experiment, the tandem cyclization reaction between *ortho*-hydroxy α-aminosulfone and bromo-substituted 1,3-dimethylbarbituric acid was subjected to an extensive optimization process. This refinement effort revealed that the reaction, when conducted with DMAP serving as the base and DCE as the solvent, was capable of producing the target product **5aa** in 80% yield.

**Scheme 3:**
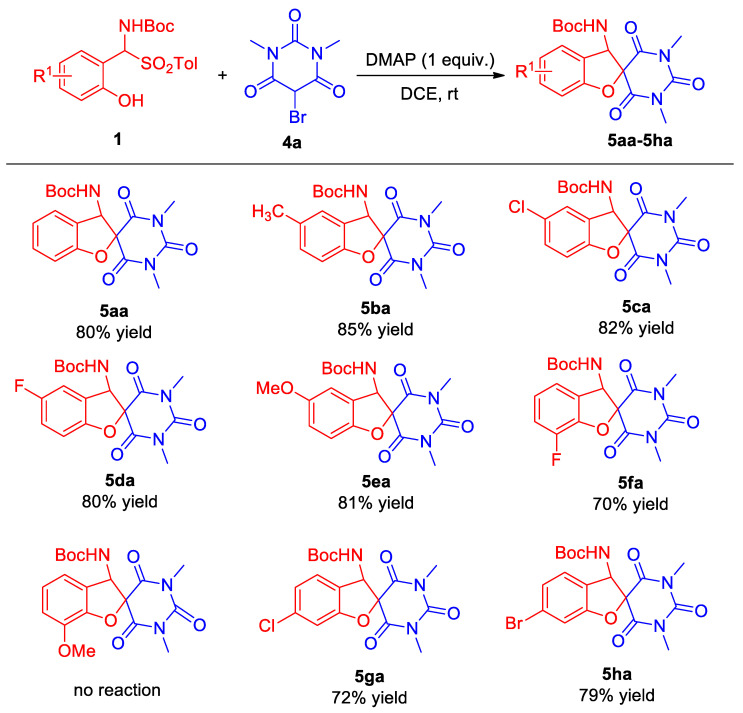
Substrate scope for benzofuranobarbituric acid. *ortho*-hydroxy α-aminosulfone **1** (0.15 mmol) and 5-bromo-1,3-dimethylbarbituric acid **4** (0.1 mmol) in DCE (1.0 mL) were stirred for 10 h at room temperature. The product yield was isolated.

Prompted by this finding, an investigation into the versatility of the reaction toward a diverse array of substituted *ortho*-hydroxy α-aminosulfone substrates was undertaken (Scheme 3). When the substituent is located at the 5-position of the aromatic ring, whether it is an electron-donating group or a halogen substituent, the target products (**5ba**–**5ea**) can be obtained with a relatively high yield (80–85%). For the 3-position-substituted *ortho*-hydroxy α-aminosulfones, their performance did not show significant improvement. Specifically, the 3-fluoro-substituted *ortho*-hydroxy α-aminosulfone only produced the target product **5fa** in 70% yield, while the 3-position electron-donating methoxy-substituted *ortho*-hydroxy α-aminosulfone failed to yield the target product. It is worthy of note that the 3-methoxy-substituted *ortho*-hydroxy α-aminosulfone also exhibited no observable reaction with 2-bromo-1,3-indandione during the substrate extension of the tandem cyclization reaction between *ortho*-hydroxy α-aminosulfone and 2-bromo-1,3-indandione. The 4-chloro- and 4-bromo-substituted *ortho*-hydroxy α-aminosulfones exhibited good performances, and **5ga** and **5ha** were obtained in 72% and 79% yields, respectively. Overall, in the substrate extension of these two parts, *ortho*-hydroxy α-aminosulfones demonstrated better performance in the tandem cyclization reaction with 2-bromo-1,3-indandione.

### 2.3. Scaled-Up Synthesis

**Scheme 4:**
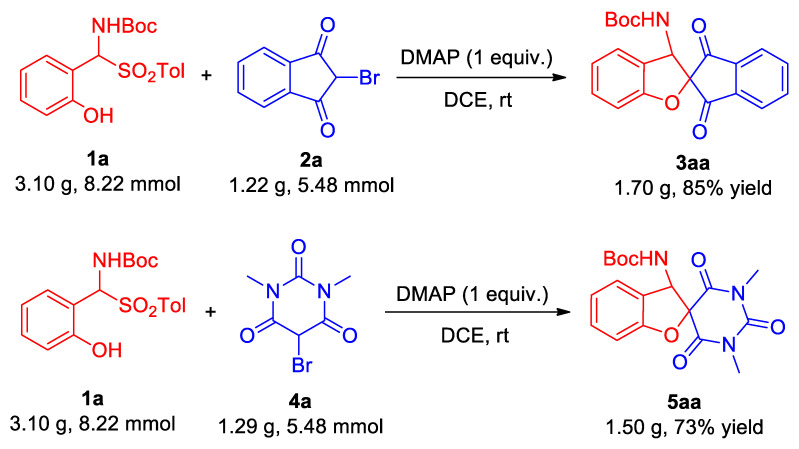
Gram-scale synthesis of **3aa** and **5aa**.

To demonstrate the practicality and stability of the DMAP-catalyzed tandem cyclization reaction involving *ortho*-hydroxy α-aminosulfone, an endeavor has also been made to conduct the reaction on a Gram-scale basis. As depicted in Scheme 4, the initial reaction scale was enlarged by approximately 55-fold. For the Gram-scale synthesis of *ortho*-hydroxy α-aminosulfone **1a** with 2-bromo-1,3-indandione **2a**, the target product **3aa** was ultimately achieved with the same high yield (85%) as in previous smaller-scale reactions. Nevertheless, in the Gram-scale reaction involving *ortho*-hydroxy α-aminosulfone **1a** and 5-bromo-1,3-dimethylbarbituric acid **4a**, the yield decreased slightly but remained moderate (73%), enabling the successful isolation of the target product **5aa**. This underscores the considerable potential of this methodology for the large-scale production of such compounds via tandem cyclization reactions.

### 2.4. Further Evaluation of Substrate Scope of Dicarbonyl α-Bromo-Substituted Compounds

**Scheme 5:**
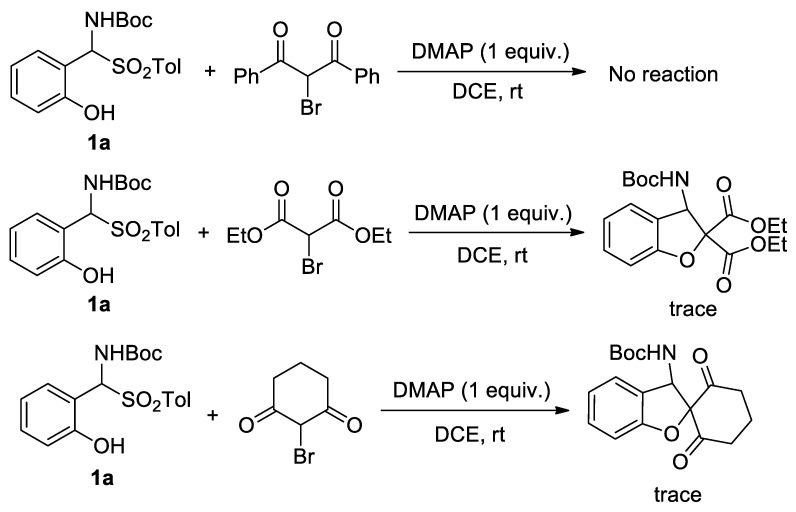
Further valuation of substrate scope.

Based on the structural features of 2-bromo-1,3-indandione and 5-bromo-1,3-dimethylbarbituric acid, several analogous dicarbonyl α-bromo-substituted compounds have been synthesized and applied to the tandem reaction with *ortho*-hydroxy α-aminosulfone **1a** (Scheme 5). Initially, linear chain-derived dicarbonyl α-bromo-substituted compounds, namely, α-bromo-substituted 1,3-diphenylpropane-1,3-dione and diethyl malonate, were used as reactants. The 2-bromo-1,3-diphenylpropane-1,3-dione was found to be unreactive with **1a**. In contrast, diethyl 2-bromomalonate exhibited reactivity, although the yield was deemed inadequate, with only a minimal amount of product observed via TLC. Moreover, a six-membered ring-derived dicarbonyl α-bromo-substituted compound, 2-bromocyclohexane-1,3-dione, was used as a reactant. However, the reaction between 2-bromocyclohexane-1,3-dione and **1a** exhibited a low efficiency.

### 2.5. Asymmetric Catalytic Reaction Trials

**Scheme 6:**
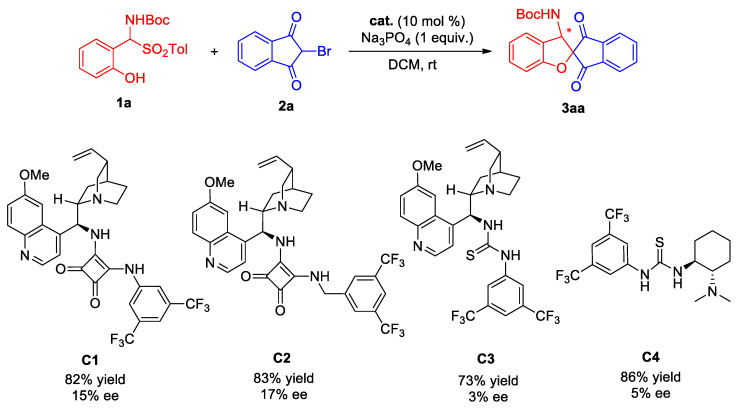
Preliminary evaluation for asymmetric catalytic reaction. * The chiral carbon, absolute configuration not determined.

Drawing upon our previous investigations into asymmetric catalysis [41,42,43], we have embarked on an extended exploration of this particular reaction, aiming for a more profound understanding. Consequently, catalysts **C1**–**C4** with varying structural types were selected for application in this asymmetric catalytic reaction (Scheme 6). Firstly, the reactions of cinchona alkaloid-derived squaramide catalysts (**C1** and **C2**) catalyzed addition/cyclization of *ortho*-hydroxy-α-aminosulfone **1a** with 2-bromo-1,3-indandione **2a** showed relatively good catalytic performance, with catalyst **C2** exhibiting the best performance. The catalytic product **3aa** was obtained in 85% yield with 17% ee. When cinchona alkaloid and 1,2-diaminocyclohexane-derived thiourea catalysts (**C3** and **C4**) were employed, the catalytic effect of the reactions was found to be even worse. Furthermore, catalyst **C1** was used to catalyze the asymmetric tandem cyclization reaction of *ortho*-hydroxy α-aminosulfone **1a** and 5-bromo-1,3-dimethylbarbituric acid **4a**, yet the enantiomeric excess of the product was only 22% ee. Through analysis of the results, it can be seen that the multifunctional hydrogen bond-derived thiourea and squaramide catalysts exhibit generally poor asymmetric catalytic performance for this type of reaction.

## 3. Materials and Methods

### 3.1. General Information

In the context of this study, commercially sourced reagents were utilized without undergoing any additional purification procedures. Commonly used solvents, including petroleum ether (boiling range 60–90 °C), ethyl acetate, dichloromethane, xylene, and methanol, as well as other analytical grade reagents, were produced by the Beijing Chemical Plant (Beijing, China). Column chromatography silica gel (200–300 mesh) and thin-layer chromatography silica gel plates were manufactured by Yantai Xinnuo New Materials Technology Co., Ltd. (Yantai, China) and Yantai Dexin Biotechnology Co., Ltd. (Yantai, China), respectively. The melting points were determined using an XT-4 melting point apparatus, and these values were reported without any correction. For nuclear magnetic resonance (NMR) analysis, ^1^H NMR spectra were acquired on a Bruker Ascend 400 MHz spectrometer, with chemical shifts expressed in δ (ppm) units, utilizing tetramethylsilane (TMS) as the internal standard for calibration. ^13^C NMR spectra were recorded at 100 MHz on the same 400 MHz spectrometer, with chemical shifts also reported in ppm, referenced to TMS, and further calibrated against the solvent peak (CDCl_3_, δC = 77.00). For high-resolution mass spectrometry, electron spray ionization (ESI) mass spectra were obtained using an Agilent 6520 Accurate-Mass Q-TOF MS system (Santa Clara, CA, USA), which was equipped with an ESI source and ensured precise and accurate mass measurements.

### 3.2. Experimental Materials for Tandem Reactions

The products **1a**–**1k** were prepared according to the literature reported by Liu and coworkers [43,44].

2-Bromo-1*H*-indene-1,3(2*H*)-dione (**2a**) was synthesized according to the literature [45,46].

A mixture of 1,3-indandione (219 mg, 1.5 mmol), KBr (835 mg, 7.5 mmol), 1 M HCl (7.5 mL), and 30% H_2_O_2_ (3.4 mL) in toluene (7.5 mL) was stirred at a room temperature. Reaction progress was monitored by TLC. Upon completion, the reaction was quenched with saturated Na_2_SO_3_ (10 mL) and neutralized with saturated NaHCO_3_ (10 mL). The organic layer was separated, extracted with EtOAc (2 × 20 mL), washed with water and brine, dried over MgSO_4_, and concentrated under vacuum to give product **2a** (300 mg, 90% yield).

5-bromo-1,3-dimethylpyrimidine-2,4,6(1*H*,3*H*,5*H*)-trione (**4a**) was prepared following the same procedure as **2a**.

The 1,3-indanedione utilized in the synthesis reaction was procured from Innochem (Beijing) Technology Co., Ltd. (Beijing, China). The potassium bromide and hydrogen peroxide were procured from Beijing MREDA Technology Co., Ltd. (Beijing, China) and Xilong Scientific Co., Ltd. (Shantou, China), respectively. The cinchona alkaloid-derived squaramide catalysts and cyclohexanediamine-derived thiourea catalyst utilized in the primary test were prepared in accordance with the literature reports [47,48,49,50].

### 3.3. Procedure for the Synthesis of Aminobenzofuran Spiroindanone ***3***

*ortho*-Hydroxy α-aminosulfone **1** (0.15 mmol), 2-bromo-1,3-indandione **2a** (0.1 mmol), and 4-dimethylaminopyridine (DMAP) (1.0 equiv.) were combined in dry DCE (1.0 mL) in a 5 mL glass reaction vessel. The reaction system was stirred at room temperature for 20 h. After the completion of the reaction, which was monitored by thin-layer chromatography, the crude product was separated by means of a silica gel rapid column chromatography (ethyl acetate/petroleum ether 1:5) to obtain **3**.
*tert*-Butyl (1′,3′-dioxo-1′,3′-dihydro-3*H*-spiro[benzofuran-2,2′-inden]-3-yl)carbamate (**3aa**). Compound **3aa** (31.0 mg, 85% yield) was isolated as a yellow solid through a standard purification process involving column chromatography on silica gel (200–300 mesh), utilizing a mixture of petroleum ether and ethyl acetate (5:1) as the eluent. m.p. 148–150 °C. ^1^H NMR (400 MHz, CDCl_3_): δ 8.09 (d, *J* = 6.8 Hz, 1H, ArH), 7.98–7.88 (m, 3H, ArH), 7.32–7.26 (m, 2H, ArH), 7.02 (t, *J* = 7.6 Hz, 1H, ArH), 6.93 (d, *J* = 8.4 Hz, 1H, ArH), 5.70 (d, *J* = 8.8 Hz, 1H, NH), 5.11 (d, *J* = 8.4 Hz, 1H, CH), 1.22 (s, 9H, CH_3_) ppm. ^13^C NMR (100 MHz, CDCl_3_): δ195.5, 194.1, 159.9, 154.9, 141.5, 141.4, 136.5, 136.3, 130.8, 124.8, 124.3, 123.7, 123.4, 122.3, 110.6, 88.2, 80.5, 59.7, 27.9 ppm. HRMS (ESI): *m*/*z* calcd. for C_21_H_19_NNaO_5_ [M + Na]^+^ 388.1156, found 388.1151.
*tert*-Butyl (5-bromo-1′,3′-dioxo-1′,3′-dihydro-3*H*-spiro[benzofuran-2,2′-inden]-3-yl)-carbamate (**3ba**). Compound **3ba** (22.2 mg, 50% yield) was isolated as a white solid through a standard purification process involving column chromatography on silica gel (200–300 mesh), utilizing a mixture of petroleum ether and ethyl acetate (5:1) as the eluent. m.p. 140–142 °C. ^1^H NMR (400 MHz, CDCl_3_): δ 8.10 (d, *J* = 6.8 Hz, 1H, ArH), 7.99–7.90 (m, 3H, ArH), 7.43–7.39 (m, 2H, ArH), 6.86 (d, J = 8.4 Hz, 1H, ArH), 5.69 (d, *J* = 8.8 Hz, 1H, NH), 5.07 (d, *J* = 8.8 Hz, 1H, CH), 1.22 (s, 9H, CH_3_) ppm. ^13^C NMR (100 MHz, CDCl_3_): δ 195.0, 193.6, 159.1, 154.7, 141.4, 136.8, 136.5, 133.7, 127.8, 126.1, 124.5, 123.5, 114.2, 112.3, 88.6, 80.9, 59.2, 27.8 ppm. HRMS (ESI): *m*/*z* calcd. for C_21_H_18_^79^BrNNaO_5_ [M + Na]^+^ 466.0261, found 466.0271; calcd. for C_21_H_18_^81^BrNNaO_5_ [M + Na]^+^ 468.0241, found 468.0252.
*tert*-Butyl (5-chloro-1′,3′-dioxo-1′,3′-dihydro-3*H*-spiro[benzofuran-2,2′-inden]-3-yl)-carbamate (**3ca**). Compound **3ca** (31.9 mg, 80% yield) was isolated as a yellow solid through a standard purification process involving column chromatography on silica gel (200–300 mesh), utilizing a mixture of petroleum ether and ethyl acetate (5:1) as the eluent. m.p. 217–219 °C. ^1^H NMR (400 MHz, CDCl_3_): δ 8.10 (d, *J* = 6.8 Hz, 1H, ArH), 7.99–7.88 (m, 3H, ArH), 7.28–7.25 (m, 2H, ArH), 6.90 (d, *J* = 8.4 Hz, 1H, ArH), 5.68 (d, *J* = 8.8 Hz, 1H, NH), 5.08 (d, *J* = 8.4 Hz, 1H, CH), 1.22 (s, 9H, CH_3_) ppm. ^13^C NMR (100 MHz, CDCl_3_): δ 195.0, 193.6, 158.6, 154.7, 141.4, 136.7, 136.5, 130.8, 127.2, 125.6, 124.9, 124.5, 123.5, 111.7, 88.6, 80.8, 59.3, 27.9 ppm. HRMS (ESI): *m*/*z* calcd. for C_21_H_18_ClNNaO_5_ [M + Na]^+^ 422.0766, found 422.0764.
*tert*-Butyl (5-fluoro-1′,3′-dioxo-1′,3′-dihydro-3*H*-spiro[benzofuran-2,2′-inden]-3-yl)carbamate (**3da**). Compound **3da** (35.3 mg, 92% yield) was isolated as a white solid through a standard purification process involving column chromatography on silica gel (200–300 mesh), utilizing a mixture of petroleum ether and ethyl acetate (5:1) as the eluent. m.p. 213–215 °C. ^1^H NMR (400 MHz, CDCl_3_): δ 8.10 (d, *J* = 6.8 Hz, 1H, ArH), 7.99–7.90 (m, 3H, ArH), 7.03–6.98 (m, 2H, ArH), 6.89 (dd, *J*_1_ = 3.8 Hz, *J*_2_ = 8.6 Hz, 1H, ArH), 5.69 (d, *J* = 8.8 Hz, 1H, NH), 5.09 (d, *J* = 8.4 Hz, 1H, CH), 1.22 (s, 9H, CH_3_) ppm. ^13^C NMR (100 MHz, CDCl_3_): δ 195.2, 193.9, 158.4 (d, ^1^*J*_C–F_ = 238.9 Hz), 155.8, 154.7, 141.4, 136.7, 136.5, 125.0 (d, ^3^*J*_C–F_ = 8.2 Hz), 124.4, 123.5, 117.4 (d, ^2^*J*_C–F_ = 24.4 Hz), 111.8 (d, ^2^*J*_C–F_ = 25.2 Hz), 111.2 (d, ^3^*J*_C–F_ = 8.3 Hz), 88.7, 80.8, 59.5, 27.9 ppm. HRMS (ESI): *m*/*z* calcd. for C_21_H_18_FNNaO_5_ [M + Na]^+^ 406.1062, found 406.1043.
*tert*-Butyl (5-methoxy-1′,3′-dioxo-1′,3′-dihydro-3*H*-spiro[benzofuran-2,2′-inden]-3-yl)carbamate (**3ea**). Compound **3ea** (29.6 mg, 75% yield) was isolated as a white solid through a standard purification process involving column chromatography on silica gel (200–300 mesh), utilizing a mixture of petroleum ether and ethyl acetate (5:1) as the eluent. m.p. 224–226 °C. ^1^H NMR (400 MHz, CDCl_3_): δ 8.09 (d, *J* = 6.8 Hz, 1H, ArH), 7.98–7.86 (m, 3H, ArH), 6.89–6.81 (m, 3H, ArH), 5.67 (d, *J* = 8.8 Hz, 1H, NH), 5.10 (d, *J* = 8.8 Hz, 1H, CH), 3.78 (s, 3H, OMe), 1.23 (s, 9H, CH_3_) ppm. ^13^C NMR (100 MHz, CDCl_3_): δ155.5, 154.8, 153.9, 141.5, 141.4, 136.5, 136.3, 124.4, 124.3, 123.4, 116.7, 110.9, 109.9, 80.6, 59.9, 56.1, 28.2, 27.9 ppm. HRMS (ESI): *m*/*z* calcd. for C_22_H_21_NNaO_6_ [M + Na]^+^ 418.1261, found 418.1252.
*tert*-Butyl (5-methyl-1′,3′-dioxo-1′,3′-dihydro-3*H*-spiro[benzofuran-2,2′-inden]-3-yl)carbamate (**3fa**). Compound **3fa** (31.1 mg, 82% yield) was isolated as a yellow solid through a standard purification process involving column chromatography on silica gel (200–300 mesh), utilizing a mixture of petroleum ether and ethyl acetate (5:1) as the eluent. m.p. 181–183 °C. ^1^H NMR (400 MHz, CDCl_3_): δ 8.08 (d, *J* = 6.8 Hz, 1H, ArH), 7.97–7.86 (m, 3H, ArH), 7.11–7.07 (m, 2H, ArH), 6.85 (d, *J* = 8.0 Hz, 1H, ArH), 5.65 (d, *J* = 8.8 Hz, 1H, NH), 5.06 (d, *J* = 8.4 Hz, 1H, CH), 2.31 (s, 3H, CH_3_), 1.22 (s, 9H, CH_3_) ppm. ^13^C NMR (100 MHz, CDCl_3_): δ 195.6, 194.3, 158.0, 154.8, 141.5, 141.4, 136.5, 136.3, 131.8, 131.3, 125.1, 124.3, 123.5, 123.4, 110.2, 88.4, 80.5, 59.8, 27.9, 20.7 ppm. HRMS (ESI): *m*/*z* calcd. for C_22_H_21_NNaO_5_ [M + Na]^+^ 402.1312, found 402.1310.
*tert*-Butyl (6-chloro-1′,3′-dioxo-1′,3′-dihydro-3*H*-spiro[benzofuran-2,2′-inden]-3-yl)carbamate (**3ga**). Compound **3ga** (32.3 mg, 81% yield) was isolated as a yellow solid through a standard purification process involving column chromatography on silica gel (200–300 mesh), utilizing a mixture of petroleum ether and ethyl acetate (5:1) as the eluent. m.p. 225–227 °C. ^1^H NMR (400 MHz, CDCl_3_): δ 8.10 (d, *J* = 6.8 Hz, 1H, ArH), 7.98–7.88 (m, 3H, ArH), 7.18 (d, *J* = 7.6 Hz, 1H, ArH), 7.02–6.98 (m, 2H, ArH), 5.65 (d, J = 8.8 Hz, 1H, NH), 5.05 (d, *J* = 8.8 Hz, 1H, CH), 1.22 (s, 9H, CH_3_) ppm. ^13^C NMR (100 MHz, CDCl_3_): δ 194.9, 193.5, 160.7, 154.8, 141.4, 136.7, 136.5, 136.4, 125.4, 124.5, 123.5, 122.7, 122.6, 111.5, 88.7, 80.8, 59.0, 27.9 ppm. HRMS (ESI): *m*/*z* calcd. for C_21_H_18_ClNNaO_5_ [M + Na]^+^ 422.0766, found 422.0765.
*tert*-Butyl (6-bromo-1′,3′-dioxo-1′,3′-dihydro-3*H*-spiro[benzofuran-2,2′-inden]-3-yl)carbamate (**3ha**). Compound **3ha** (37.7 mg, 85% yield) was isolated as a white solid through a standard purification process involving column chromatography on silica gel (200–300 mesh), utilizing a mixture of petroleum ether and ethyl acetate (5:1) as the eluent. m.p. 164–166 °C. ^1^H NMR (400 MHz, CDCl_3_): δ 8.09 (d, *J* = 6.8 Hz, 1H, ArH), 7.98–7.88 (m, 3H, ArH), 7.17–7.12 (m, 3H, ArH), 5.63 (d, *J* = 8.8 Hz, 1H, NH), 5.07 (d, *J* = 8.4 Hz, 1H, CH), 1.22 (s, 9H, CH_3_) ppm. ^13^C NMR (100 MHz, CDCl_3_): δ 194.9, 193.5, 160.7, 154.8, 141.4, 136.7, 136.5, 125.8, 125.5, 124.4, 124.1, 123.5, 123.1, 114.4, 88.6, 80.8, 59.1, 27.9 ppm. HRMS (ESI): *m*/*z* calcd. for C_21_H_18_^79^BrNNaO_5_ [M + Na]^+^ 466.0261, found 466.0271; calcd. for C_21_H_18_^81^BrNNaO_5_ [M + Na]^+^ 468.0241, found 468.0252.
*tert*-Butyl (7-bromo-1′,3′-dioxo-1′,3′-dihydro-3*H*-spiro[benzofuran-2,2′-inden]-3-yl)carbamate (**3ia**). Compound **3ia** (39.9 mg, 90% yield) was isolated as a yellow solid through a standard purification process involving column chromatography on silica gel (200–300 mesh), utilizing a mixture of petroleum ether and ethyl acetate (5:1) as the eluent. m.p. 172–174 °C. ^1^H NMR (400 MHz, CDCl_3_): δ 8.10 (d, *J* = 6.8 Hz, ArH), 7.99–7.88 (m, 3H, ArH), 7.46 (d, *J* = 8.0 Hz, 1H, ArH), 7.21 (d, *J* = 7.6 Hz, 1H, ArH), 6.92 (t, *J* = 7.6 Hz, 1H, ArH), 5.77 (d, *J* = 8.8 Hz, 1H, NH), 5.12 (d, *J* = 8.8 Hz, 1H, CH), 1.23 (s, 9H, CH_3_) ppm. ^13^C NMR (100 MHz, CDCl_3_): δ 194.8, 193.3, 157.2, 154.8, 141.5 136.7, 136.5, 133.9, 125.2, 124.5, 123.8, 123.7, 123.5, 103.4, 87.6, 80.8, 60.0, 27.9 ppm. HRMS (ESI): *m*/*z* calcd. for C_21_H_18_^79^BrNNaO_5_ [M + Na]^+^ 466.0261, found 466.0271, C_21_H_18_^81^BrNNaO_5_ [M + Na]^+^ 468.0241, found 468.0264.
*tert*-Butyl (7-fluoro-1′,3′-dioxo-1′,3′-dihydro-3*H*-spiro[benzofuran-2,2′-inden]-3-yl)carbamate (**3ja**). Compound **3ja** (36.4 mg, 95% yield) was isolated as a yellow solid through a standard purification process involving column chromatography on silica gel (200–300 mesh), utilizing a mixture of petroleum ether and ethyl acetate (5:1) as the eluent. m.p. 175–177 °C. ^1^H NMR (400 MHz, CDCl_3_): δ 8.10 (d, *J* = 6.4 Hz, 1H, ArH), 7.99–7.90 (m, 3H, ArH), 7.12–7.05 (m, 2H, ArH), 7.00–6.95 (m, 1H, ArH), 5.74 (d, *J* = 8.8 Hz, 1H, NH), 5.12 (d, *J* = 8.8 Hz, 1H, CH), 1.23 (s, 9H, CH_3_) ppm. ^13^C NMR (100 MHz, CDCl_3_): δ 194.7, 193.3, 154.8, 147.2 (d, ^1^*J* _C–F_ = 247.0 Hz), 146.7 (^2^*J*_C–F_ = 11.6 Hz), 141.44, 141.36, 136.7, 136.5, 127.2, 124.5, 123.5, 123.1 (d, ^3^*J* _C–F_ = 5.5 Hz), 120.0 (d, ^3^*J*_C–F_ = 3.3 Hz), 117.9 (d, ^2^*J*_C–F_ = 16.5 Hz), 88.5, 80.8, 59.6, 27.8 ppm. HRMS (ESI): *m*/*z* calcd. for C_21_H_18_FNNaO_5_ [M + Na]^+^ 406.1062, found 406.1057.
*tert*-Butyl (4-chloro-1′,3′-dioxo-1′,3′-dihydro-3*H*-spiro[benzofuran-2,2′-inden]-3-yl)carbamate (**3ka**). Compound **3ka** (32.3 mg, 81% yield) was isolated as a white solid through a standard purification process involving column chromatography on silica gel (200–300 mesh), utilizing a mixture of petroleum ether and ethyl acetate (5:1) as the eluent. m.p. 178–180 °C. ^1^H NMR (400 MHz, CDCl_3_): δ 8.10–8.08 (m, 1H, ArH), 7.99–7.89 (m, 3H, ArH), 7.26 (t, *J* = 8.2 Hz, 1H, ArH), 6.99 (d, *J* = 8.0 Hz, 1H, ArH), 6.88 (d, J = 8.4 Hz, 1H, ArH), 5.73 (d, *J* = 8.8 Hz, 1H, NH), 5.07 (d, *J* = 8.4 Hz, 1H, CH), 1.27 (s, 9H, CH_3_) ppm. ^13^C NMR (176 MHz, CDCl_3_): δ 194.7, 193.1, 161.1, 154.7, 141.8, 140.8, 136.7, 136.6, 132.0, 131.3, 124.5, 123.5, 122.9, 121.6, 109.2, 88.2, 80.6, 58.9, 27.9 ppm. HRMS (ESI): *m*/*z* calcd. for C_21_H_18_ClNNaO_5_ [M + Na]^+^ 422.0766, found 422.0748.

### 3.4. Procedure for the Synthesis of Aminobenzofuran Spirobarbituric ***5***

A mixture comprising *ortho*-hydroxy α-aminosulfone **1** (0.15 mmol), 5-bromo-1,3-dimethylpyrimidine-2,4,6(1*H*,3*H*,5*H*)-trione **4a** (0.1 mmol), and 4-dimethlaminopyridine (DMAP) (1.0 equiv.) was assembled in anhydrous dichloroethane (DCE, 1.0 mL) within a 10 mL glass reaction vessel. This reaction ensemble was agitated at ambient temperature for 10 h. Upon completion of the reaction, monitored via thin-layer chromatography (TLC), the crude product mixture underwent purification via silica gel flash column chromatography, employing a solvent system of ethyl acetate and petroleum ether in a ratio of 1:5, affording the desired product **5**.

*tert*-Butyl (1′,3′-dimethyl-2′,4′,6′-trioxo-1′,3′,4′,6′-tetrahydro-2′*H*,3*H*-spiro[benzofuran-2,5′-pyrimidin]-3-yl)carbamate (**5aa**). Compound 5aa (30.0 mg, 80% yield) was isolated as a white solid through a standard purification process involving column chromatography on silica gel (200–300 mesh), utilizing a mixture of petroleum ether and ethyl acetate (5:1) as the eluent. m.p. 198–200 °C. ^1^H NMR (400 MHz, CDCl_3_): *δ* 7.33 (t, *J* = 7.6 Hz, 1H, ArH), 7.22 (d, *J* = 7.6 Hz, 1H, ArH), 7.03–7.00 (m, 2H, ArH), 5.75 (d, *J* = 8.4 Hz, 1H, NH), 5.14 (d, *J* = 8.4 Hz, 1H, CH), 3.40 (s, 3H, CH_3_), 3.30 (s, 3H, CH_3_), 1.43 (s, 9H, CH_3_) ppm. ^13^C NMR (100 MHz, CDCl_3_): *δ* 167.4, 165.2, 159.9, 155.0, 150.3, 131.2, 124.7, 122.5, 121.9, 110.8, 87.6, 81.3, 63.9, 29.2, 28.8, 28.1 ppm. HRMS (ESI): *m*/*z* calcd. for C_18_H_21_N_3_NaO_6_ [M + Na]^+^ 398.1322, found398.1307.
*tert*-Butyl (1′,3′,5-trimethyl-2′,4′,6′-trioxo-1′,3′,4′,6′-tetrahydro-2′*H*,3*H*-spiro[benzo-furan-2,5′-pyrimidin]-3-yl)carbamate (**5ba**). Compound 5ba (31.1 mg, 85% yield) was isolated as a white solid through a standard purification process involving column chromatography on silica gel (200–300 mesh), utilizing a mixture of petroleum ether and ethyl acetate (5:1) as the eluent. m.p. 203–205 °C. ^1^H NMR (400 MHz, CDCl_3_): *δ* 7.11 (d, *J* = 8.4 Hz, 1H, ArH), 7.02 (s, 1H, ArH), 6.90 (d, *J* = 8.4 Hz, 1H, ArH), 5.70 (d, *J* = 8.8 Hz, 1H, NH), 5.12 (d, *J* = 8.4 Hz, 1H, CH), 3.38 (s, 3H, CH_3_), 3.28 (s, 3H, CH_3_), 2.30 (s, 3H, CH_3_), 1.42 (s, 9H, CH_3_) ppm. ^13^C NMR (176 MHz, CDCl_3_): *δ* 167.5, 165.3, 157.9, 154.9, 150.3, 132.0, 131.7, 124.9, 121.7, 110.3, 87.8, 81.3, 63.9, 29.2, 28.7, 28.1, 20.7 ppm. HRMS (ESI): *m*/*z* calcd. for C_19_H_23_N_3_NaO_6_ [M + Na]^+^ 412.1479, found 412.1471.
*tert*-Butyl (5-chloro-1′,3′-dimethyl-2′,4′,6′-trioxo-1′,3′,4′,6′-tetrahydro-2′*H*,3*H*-spiro-[benzofuran-2,5′-pyrimidin]-3-yl)carbamate (**5ca**). Compound 5ca (33.5 mg, 82% yield) was isolated as a white solid through a standard purification process involving column chromatography on silica gel (200–300 mesh), utilizing a mixture of petroleum ether and ethyl acetate (5:1) as the eluent. m.p. 218–220 °C. ^1^H NMR (400 MHz, CDCl_3_): *δ* 7.29 (dd, *J*_1_ = 8.8 Hz, *J*_2_ = 2.0 Hz, 1H, ArH), 7.21 (s, 1H, ArH), 6.95 (d, *J* = 8.4 Hz, 1H, ArH), 5.73 (d, *J* = 8.8 Hz, 1H, NH), 5.12 (d, *J* = 8.4 Hz, 1H, CH), 3.39 (s, 3H, CH_3_), 3.29 (s, 3H, CH_3_), 1.42 (s, 9H, CH_3_) ppm. ^13^C NMR (176 MHz, CDCl_3_): *δ* 167.0, 164.8, 158.5, 154.8, 150.1, 131.2, 127.4, 124.8, 123.8, 111.8, 87.9, 81.6, 63.4, 29.3, 28.8, 28.1 ppm. HRMS (ESI): *m*/*z* calcd. for C_18_H_20_ClN_3_NaO_6_ [M + Na]^+^ 432.0933, found 432.0924.
*tert*-Butyl (5-fluoro-1′,3′-dimethyl-2′,4′,6′-trioxo-1′,3′,4′,6′-tetrahydro-2′*H*,3*H*-spiro-[benzofuran-2,5′-pyrimidin]-3-yl)carbamate (**5da**). Compound 5da (31.4 mg, 80% yield) was isolated as a white solid through a standard purification process involving column chromatography on silica gel (200–300 mesh), utilizing a mixture of petroleum ether and ethyl acetate (5:1) as the eluent. m.p. 205–207 °C. ^1^H NMR (400 MHz, CDCl_3_): *δ* 7.02 (td, *J*_1_ = 8.8 Hz, *J*_2_ = 2.4 Hz, 1H, ArH), 6.96–6.93 (m, 2H, ArH), 5.74 (d, *J* = 8.8 Hz, 1H, NH), 5.19 (d, *J* = 8.4 Hz, 1H, CH), 3.39 (s, 3H, CH_3_), 3.29 (s, 3H, CH_3_), 1.42 (s, 9H, CH_3_) ppm. ^13^C NMR (176 MHz, CDCl_3_): *δ* 167.2, 165.0, 158.4 (^1^*J*_C–F_ = 240.8 Hz), 155.7, 154.9, 150.1, 123.2 (^3^*J*_C–F_ = 8.8 Hz), 117.8 (^2^*J*_C–F_ = 24.6 Hz), 111.7 (^2^*J*_C-F_ = 25.7 Hz), 111.3 (^3^*J*_C–F_ = 8.1 Hz), 88.0, 81.6, 63.6, 29.2, 28.8, 28.1 ppm. HRMS (ESI): *m*/*z* calcd. for C_18_H_20_FN_3_NaO_6_ [M + Na]^+^ 416.1228, found 416.1209.
*tert*-Butyl (5-methoxy-1′,3′-dimethyl-2′,4′,6′-trioxo-1′,3′,4′,6′-tetrahydro-2′*H*,3*H*-spiro-[benzofuran-2,5′-pyrimidin]-3-yl)carbamate (**5ea**). Compound 5ea (32.8 mg, 81% yield) was isolated as a white solid through a standard purification process involving column chromatography on silica gel (200–300 mesh), utilizing a mixture of petroleum ether and ethyl acetate (5:1) as the eluent. m.p. 228–230 °C. ^1^H NMR (400 MHz, CDCl_3_): *δ* 6.93 (d, *J* = 8.8 Hz, 1H, ArH), 6.86 (dd, *J*_1_ = 2.6 Hz, *J*_2_ = 9.0 Hz, 1H, ArH), 6.74 (d, *J* = 2.0 Hz, 1H, ArH), 5.72 (d, *J* = 8.8 Hz, 1H, NH), 5.13 (d, *J* = 8.4 Hz, 1H, CH), 3.76 (s, 3H, OCH_3_), 3.39 (s, 3H, CH_3_), 3.29 (s, 3H, CH_3_), 1.43 (s, 9H, CH_3_) ppm. ^13^C NMR (176 MHz, CDCl_3_): *δ* 167.5, 165.3, 155.5, 154.9, 153.8, 150.3, 122.5, 117.0, 111.0, 109.8, 87.9, 81.4, 64.0, 56.1, 29.2, 28.7, 28.1 ppm. HRMS (ESI): *m*/*z* calcd. for C_19_H_23_N_3_NaO_7_ [M + Na]^+^ 428.1428, found 428.1417.
*tert*-Butyl (7-fluoro-1′,3′-dimethyl-2′,4′,6′-trioxo-1′,3′,4′,6′-tetrahydro-2′*H*,3*H*-spiro-[benzofuran-2,5′-pyrimidin]-3-yl)carbamate (**5fa**). Compound 5fa (27.5 mg, 70% yield) was isolated as a white solid through a standard purification process involving column chromatography on silica gel (200–300 mesh), utilizing a mixture of petroleum ether and ethyl acetate (5:1) as the eluent. m.p. 201–203 °C. ^1^H NMR (400 MHz, CDCl_3_): *δ* 7.13–7.09 (m, 1H, ArH), 7.01–6.94 (m, 2H, ArH), 5.79 (d, *J* = 8.4 Hz, 1H, NH), 5.17 (d, *J* = 8.4 Hz, 1H, CH), 3.40 (s, 3H, CH_3_), 3.30 (s, 3H, CH_3_), 1.42 (s, 9H, CH_3_) ppm. ^13^C NMR (100 MHz, CDCl_3_): *δ* 166.8, 164.5, 155.0, 150.1, 147.1 (^1^*J*_C–F_ = 247.6 Hz), 146.7 (^2^*J*_C–F_ = 11.5 Hz), 125.5 (^4^*J*_C–F_ = 2.5 Hz), 123.3 (^3^*J*_C–F_ = 5.3 Hz), 119.8 (^3^*J*_C–F_ = 3.4 Hz), 118.2 (^2^*J*_C–F_ = 16.4 Hz), 87.9, 81.6, 63.9, 29.2, 28.8, 28.1 ppm. HRMS (ESI): *m*/*z* calcd. for C_18_H_20_FN_3_NaO_6_ [M + Na]^+^ 416.1228, found 416.1224.
*tert*-Butyl (6-chloro-1′,3′-dimethyl-2′,4′,6′-trioxo-1′,3′,4′,6′-tetrahydro-2′*H*,3*H*-spiro-[benzofuran-2,5′-pyrimidin]-3-yl)carbamate (**5ga**). Compound 5ga (29.4 mg, 72% yield) was isolated as a white solid through a standard purification process involving column chromatography on silica gel (200–300 mesh), utilizing a mixture of petroleum ether and ethyl acetate (5:1) as the eluent. m.p. 202–204 °C. ^1^H NMR (400 MHz, CDCl_3_): *δ* 7.14 (d, *J* = 8.0 Hz, 1H, ArH), 7.03–6.99 (m, 2H, ArH), 5.70 (d, *J* = 8.8 Hz, 1H, NH), 5.10 (d, *J* = 8.4 Hz, 1H, CH), 3.39 (s, 3H, CH_3_), 3.29 (s, 3H, CH_3_), 1.42 (s, 9H, CH_3_) ppm. ^13^C NMR (100 MHz, CDCl_3_): *δ* 166.9, 164.8, 160.5, 154.9, 150.1, 136.8, 125.3, 122.8, 120.7, 111.6, 88.0, 81.6, 63.2, 29.2, 28.8, 28.1 ppm. HRMS (ESI): *m*/*z* calcd. for C_18_H_20_ClN_3_NaO_6_ [M + Na]^+^ 432.0933, found 432.0928.
*tert*-Butyl (6-bromo-1′,3′-dimethyl-2′,4′,6′-trioxo-1′,3′,4′,6′-tetrahydro-2′*H*,3*H*-spiro-[benzofuran-2,5′-pyrimidin]-3-yl)carbamate (**5ha**). Compound 5ha (35.8 mg, 79% yield) was isolated as a white solid through a standard purification process involving column chromatography on silica gel (200–300 mesh), utilizing a mixture of petroleum ether and ethyl acetate (5:1) as the eluent. m.p. 198–200 °C. ^1^H NMR (400 MHz, CDCl_3_): *δ* 7.18–7.14 (m, 2H, ArH), 7.08 (d, *J* = 8.0 Hz, 1H, ArH), 5.68 (d, *J* = 8.8 Hz, 1H, NH), 5.20 (d, *J* = 8.8 Hz, 1H, CH), 3.38 (s, 3H, CH_3_), 3.27 (s, 3H, CH_3_), 1.42 (s, 9H, CH_3_) ppm. ^13^C NMR (100 MHz, CDCl_3_): *δ* 166.9, 164.7, 160.5, 154.9, 150.1, 125.7, 125.6, 124.4, 121.3, 114.4, 87.9, 81.5, 63.3, 29.2, 28.7, 28.1 ppm. HRMS (ESI): *m*/*z* calcd. for C_18_H_21_^79^BrN_3_O_6_ [M + H]^+^ 454.0608, found 454.0305, calcd. for C_18_H_21_^81^BrN_3_O_6_ [M + H]^+^ 456.0588, found 456.0287.

### 3.5. Procedure for the Scaled-Up Synthesis of Compound ***3aa*** and ***5aa***

A 100 mL round-bottomed flask was charged with *ortho*-hydroxy α-aminosulfone **1a** (3.10 g, 8.22 mmol), 2-bromo-1*H*-indene-1,3(2*H*)-dione **2a** (1.22 g, 5.48 mmol), and 4-dimethylaminopyridine (DMAP) (668.6 mg, 5.48 mmol). The mixture was dissolved in 55 mL of DCE and stirred at room temperature while monitoring the reaction progress using TLC. After completion of the reaction, the resulting mixture was separated and purified via silica gel column chromatography using an eluent composed of ethyl acetate/petroleum ether in a ratio of 1:5. Finally, a product yield of 85% (1.70 g) for compound **3aa** was achieved.

The Gram-scale preparation method of **5aa** is analogous to the aforementioned **3aa**.

### 3.6. Asymmetric Catalyzed Cyclization

The *ortho*-hydroxy α-aminosulfone **1a** (56.6 mg, 0.15 mmol), 2-bromo-1,3-indandione **2a** (22.3 mg, 0.10 mmol), trisodium phosphate (38 mg, equimolar amount), and catalyst **C2** (6.5 mg, 0.5 mol%) were combined in a 5 mL glass vial. The mixture was dissolved in dichloromethane (1.0 mL) containing catalyst **C2** at a concentration of 10 mol%. The reaction system was then stirred at room temperature until complete conversion of substrate **2a** was confirmed by thin-layer chromatography analysis. Subsequently, the solvent was evaporated under vacuum rotation conditions, and the resulting product was purified using rapid column chromatography on silica gel with an eluent consisting of ethyl acetate/petroleum ether ratios ranging from 1:8 to 1:5, yielding catalytic product **3aa** (30.3 mg, 83% yield, 17% ee).

## 4. Conclusions

In summary, a comprehensive series of benzofuran-fused indanone and barbiturate derivatives were synthesized with remarkable success through tandem cyclization reactions, featuring 2-bromo-1,3-indenedione, 5-bromo-1,3-dimethylbarbituric acid, and *ortho*-hydroxy α-aminosulfones as pivotal reactants. By meticulously optimizing the reaction conditions with DMAP as the base catalyst, DCE as the solvent, and gentle stirring at room temperature, we achieved exceptional yields of up to 95% and 85% for benzofuran-fused indanone and barbiturate derivatives, respectively. The extension of the substrate scope and the successful execution of Gram-scale syntheses underscored the robustness and versatility of this methodology, significantly expanding the benzofuran derivative library and facilitating further biological evaluations.

## Figures and Tables

**Table 1:** Optimization of reaction conditions ^a^.

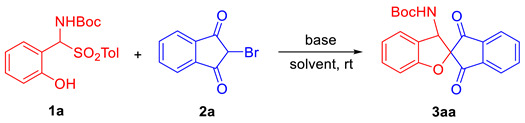				
Entry	Catalyst	Solvent	Time (h)	Yield ^b^ (%)
1	NaHCO_3_	CH_2_Cl_2_	20	42
2	Na_2_CO_3_	CH_2_Cl_2_	20	50
3	K_2_CO_3_	CH_2_Cl_2_	20	52
4	Cs_2_CO_3_	CH_2_Cl_2_	20	41
5	Et_3_N	CH_2_Cl_2_	20	45
6	DABCO	CH_2_Cl_2_	20	31
7	DBU	CH_2_Cl_2_	20	32
8	TMG	CH_2_Cl_2_	20	36
9	DMAP	CH_2_Cl_2_	20	70
10	DMAP	DCE	20	85
11	DMAP	toluene	20	54
12	DMAP	CH_3_CN	20	41
13	DMAP	THF	20	33
14	DMAP	EtOH	15	25
15	DMAP	MTBE	16	30

^a^ Unless otherwise specified, the reaction conditions are as follows: *ortho*-hydroxy α-aminosulfone **1a** (0.15 mmol), 2-bromo-1,3-indandione **2a** (0.1 mmol), and base (1.0 equiv.) were dissolved in a solvent (1.0 mL), and the reaction was stirred at room temperature. ^b^ The yield of silica gel column chromatography separation.

## Data Availability

Data are contained within this article and Supplementary Materials.

## Supplementary Materials

The following supporting information can be downloaded at https://www.mdpi.com/article/10.3390/molecules29163725/s1, Copies of ^1^H and ^13^C NMR spectra of new compounds.
