# Supplementary material for: Synthesis of Benzofuran Derivatives via a DMAP-Mediated Tandem Cyclization Reaction Involving ortho-Hydroxy α-Aminosulfones
Source: Molecules. 2024 Aug 6;29(16):3725. doi: 10.3390/molecules29163725 (PMC11357171; doi:10.3390/molecules29163725)
Supplement: Supplementary file 1 [file molecules-29-03725-s001.zip › molecules-3136992-supplementary.pdf]

Synthesis of Benzofuran Derivatives via a DMAP-Mediated Tandem Cyclization Reaction Involving *ortho*-Hydroxy α-Aminosulfones

Rong-Rong Zhu, Xi-Qiang Hou and Da-Ming Du*

School of Chemistry and Chemical Engineering, Beijing Institute of Technology; Key Laboratory of Medicinal Molecule Science and Pharmaceutical Technology, Ministry of Industry and Information Technology, No. 5 Zhongguancun South Street, Beijing 100081, China

E-mail: dudm@bit.edu.cn

Supporting Information

Contents

1. Example synthetic procedure of organocatalyst for asymmetric catalyzed cyclization.....S1
2. Copies of ^{1}H and ^{13}C NMR spectra of new spirocyclic benzofuran derivatives S2

1. Example synthetic procedure of organocatalyst for asymmetric catalyzed cyclization

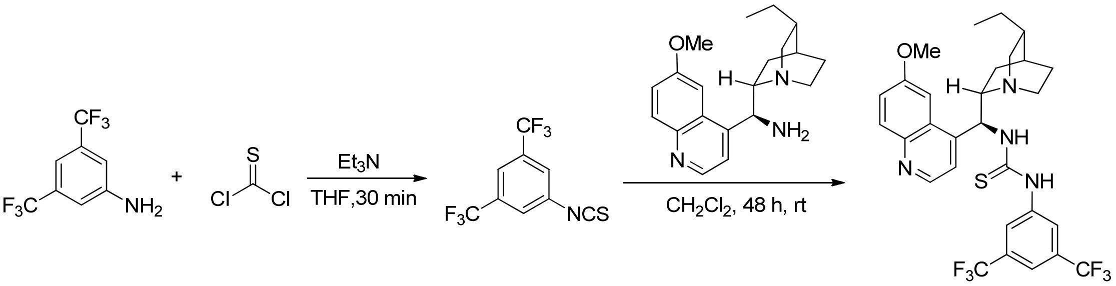

C3

Catalyst **C3** as an illustrative example. Initially, a pre-dried 50 mL round-bottom flask was charged with 20 mL of tetrahydrofuran (THF) to ensure a dry reaction environment. Subsequently, 3,5-bis(trifluoromethyl)aniline (0.23 g, 1.0 mmol, 1.0 equiv.) was added to the flask, and thiophosgene (13.68 mg, 1.2 equiv.) was precisely injected via a syringe in order to ensure thorough mixing and stirring.

Subsequently, under a controlled temperature of 0 °C, triethylamine dissolved in THF (2.4 equiv. in 10 mL THF) was gradually introduced into the reaction system, and stirring was continued for approximately 30 min. Afterward, the temperature of the system was gradually raised to room temperature, and stirring was maintained for an additional 2 h.

Once the reaction was complete, the reaction mixture was concentrated by rotary evaporation, and the residue was dissolved in ethyl acetate. To remove potential impurities, the mixture was washed with a saturated sodium bicarbonate solution and then dried with anhydrous sodium sulfate. After filtering the suspension and further concentration, the mixture was purified using a silica gel chromatography column, employing petroleum ether as the eluent, ultimately yielding the desired orange oily liquid. In the subsequent synthesis of catalyst **C3**, 0.33 g (1.0 mmol) of hydroquinine amine were dissolved in 10 mL of dichloromethane. Additionally, 0.27 g (1.0 mmol) of 3,5-bis(trifluoromethyl)phenyl isothiocyanate, obtained in the previous step, were added. The mixture was stirred at room temperature for up to 48 h, with the reaction progress monitored using thin-layer chromatography (TLC). Upon confirmation of complete consumption of the reactants, the solvent was removed using reduced-pressure rotary evaporation, and the concentrate was purified by silica gel column chromatography (methanol/ethyl acetate 5:95). The resulting white solid catalyst was obtained (0.51 g, 81% yield).

Other chiral catalysts were prepared strictly according to the literature.

2. Copies of ^1H, ^{13}C NMR spectra of new spirocyclic benzofuran derivatives.

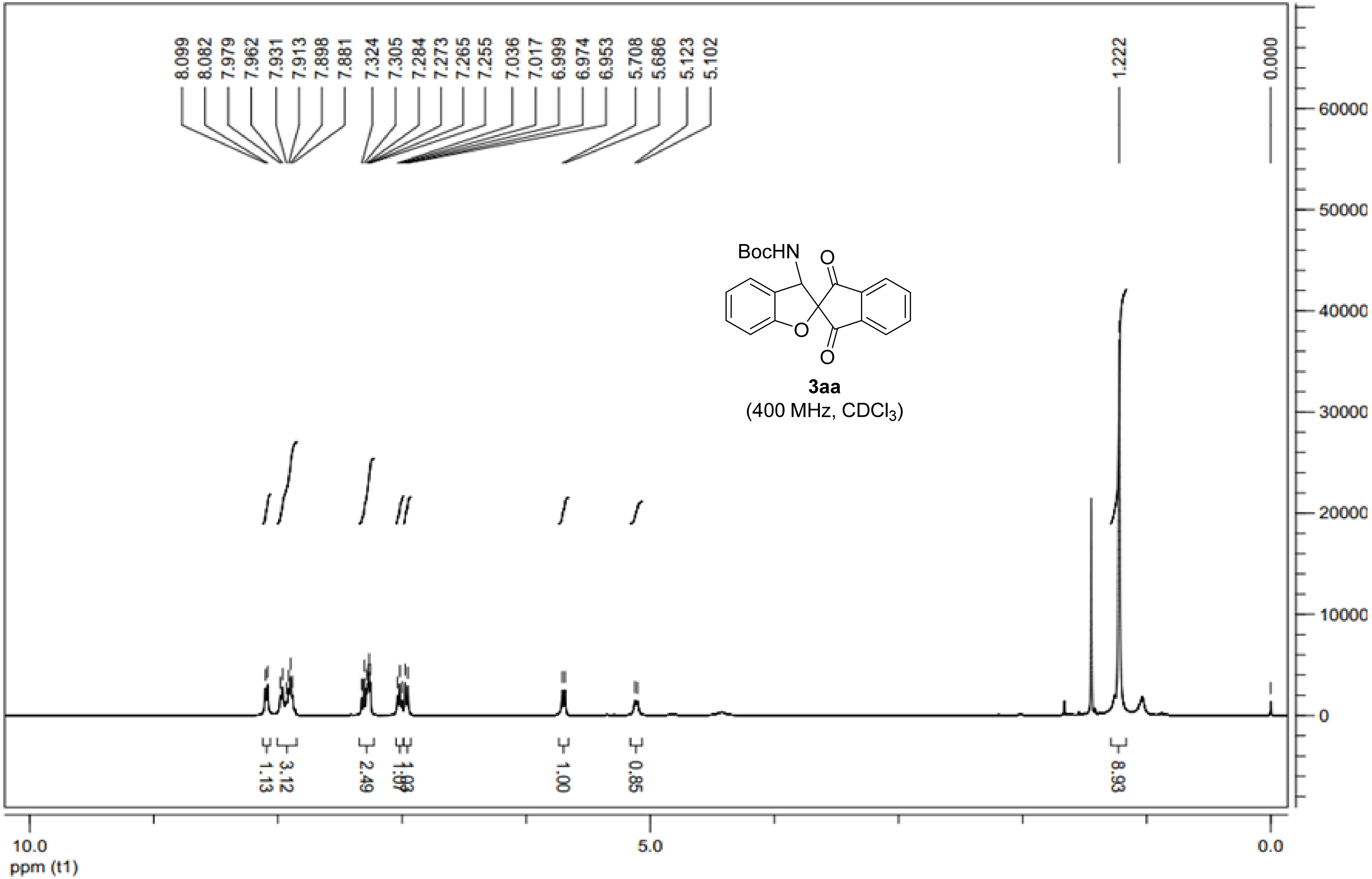

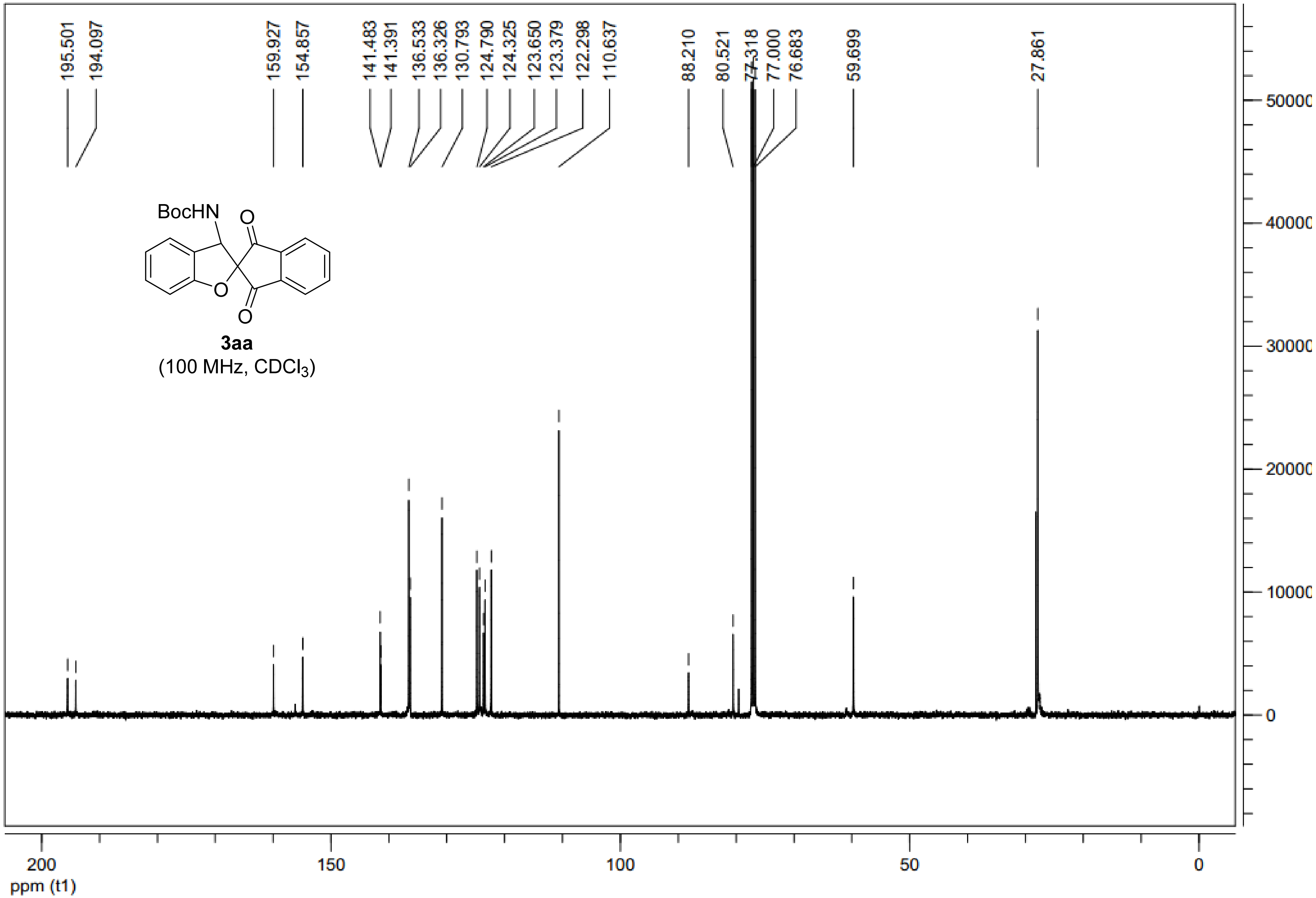
195.501
194.097
159.927
154.857
141.483
141.391
136.533
136.326
130.793
124.790
124.325
123.650
123.379
122.298
110.637
88.210
80.521
77.318
77.000
76.683
59.699
27.861
BocHN
O
O
O
3aa
(100 MHz, CDCl3)
50000
40000
30000
20000
10000
0
200
150
100
50
0
ppm (t1)

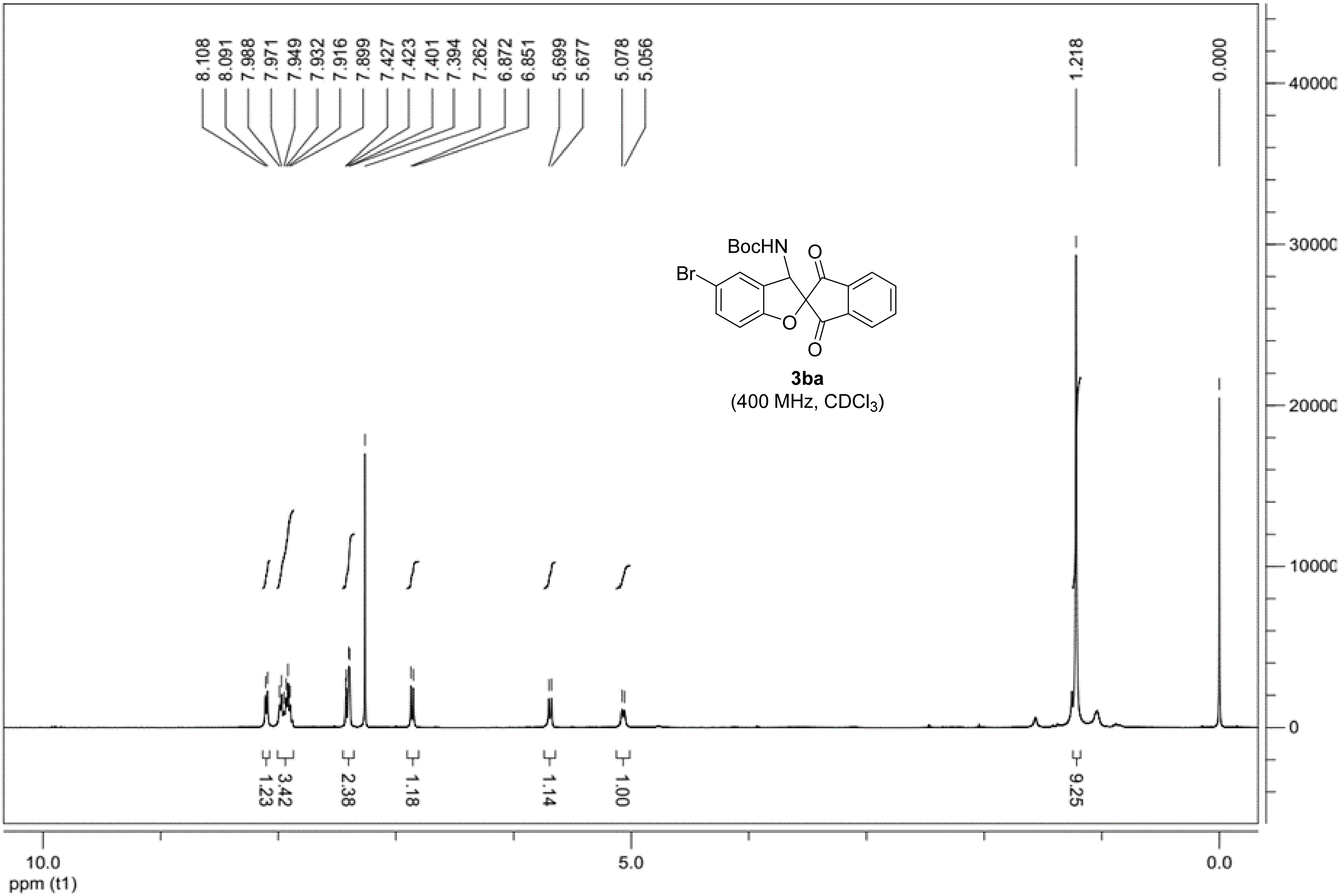

8.108
8.091
7.988
7.971
7.949
7.932
7.916
7.899
7.427
7.423
7.401
7.394
7.262
6.872
6.851
5.699
5.677
5.078
5.056
1.218
0.000
BocHN
Br
O
O
O
3ba
(400 MHz, CDCl3)
1.23
3.42
2.38
1.18
1.14
1.00
9.25
10.0
5.0
0.0
ppm (t1)
40000
30000
20000
10000
0

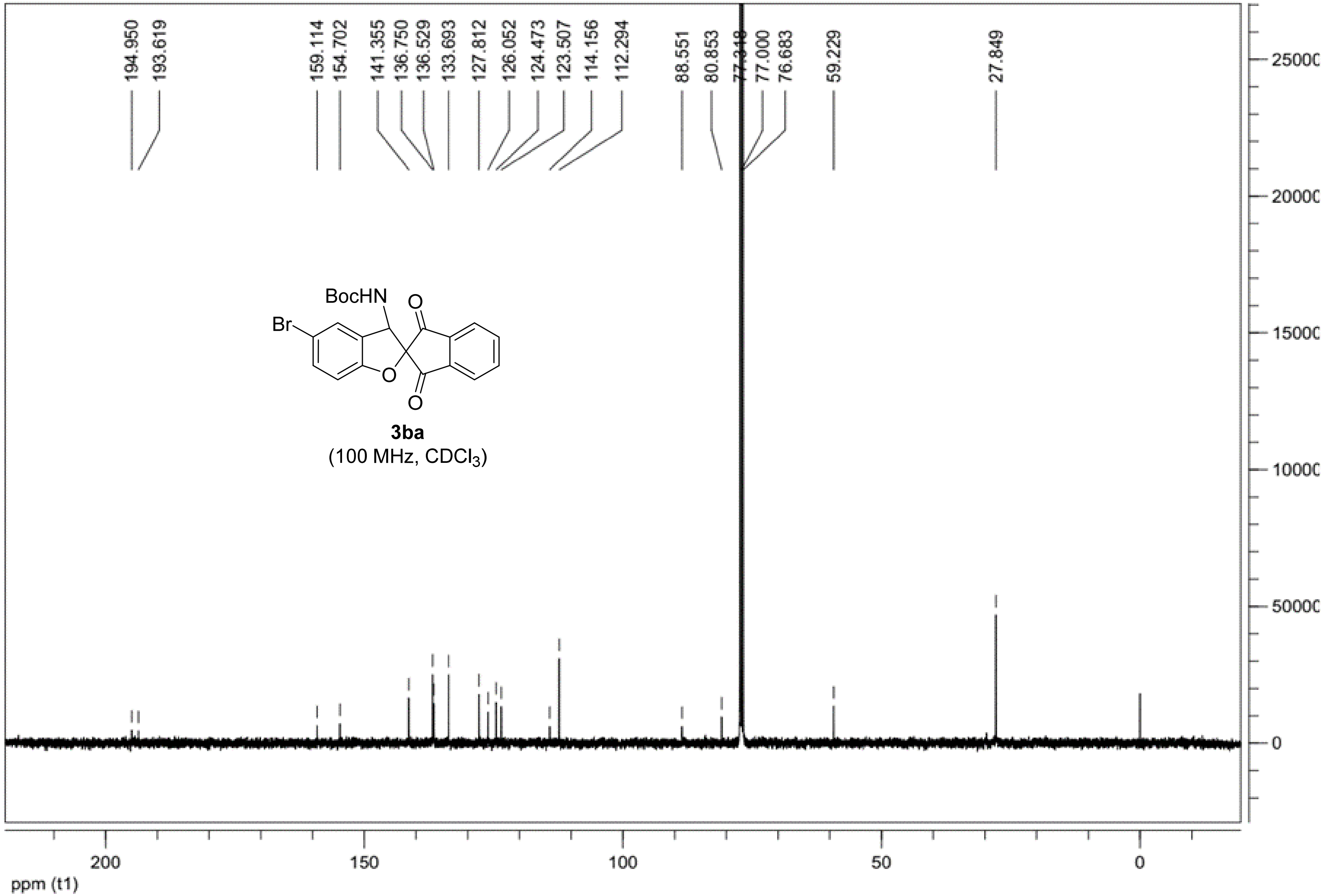

3ba
(100 MHz, $CDCl_3$)

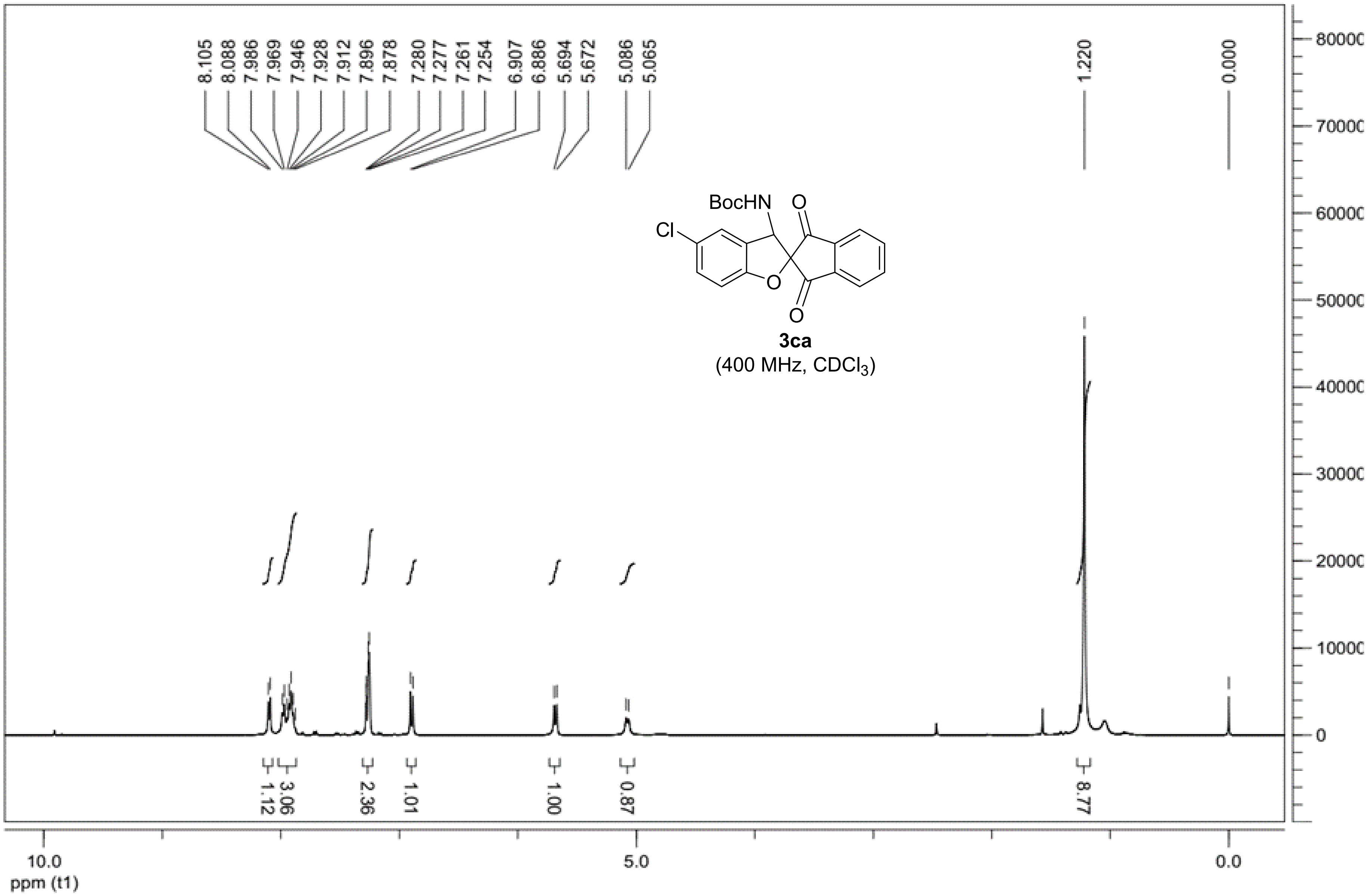
8.105
8.088
7.986
7.969
7.946
7.928
7.912
7.896
7.878
7.280
7.277
7.261
7.254
6.907
6.886
5.694
5.672
5.086
5.065
1.220
0.000
BocHN
Cl
O
3ca
(400 MHz, CDCl3)
1.12
3.06
2.36
1.01
1.00
0.87
8.77
10.0
5.0
0.0
ppm (t1)

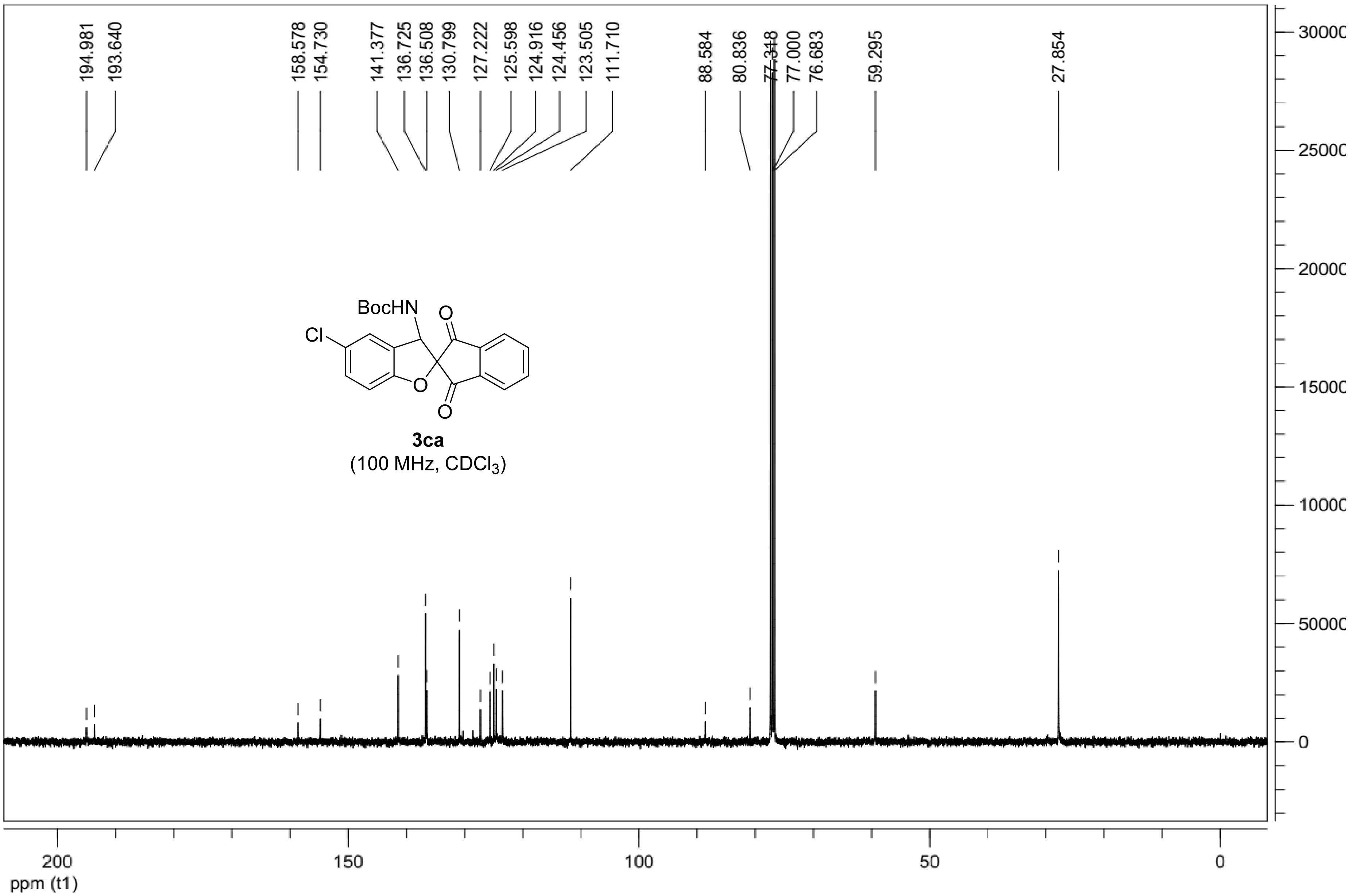
194.981
193.640
158.578
154.730
141.377
136.725
136.508
130.799
127.222
125.598
124.916
124.456
123.505
111.710
88.584
80.836
77.318
77.000
76.683
59.295
27.854
BocHN
Cl
O
O
O
3ca
(100 MHz, CDCl3)
200
150
100
50
0
ppm (t1)
30000
25000
20000
15000
10000
50000
0

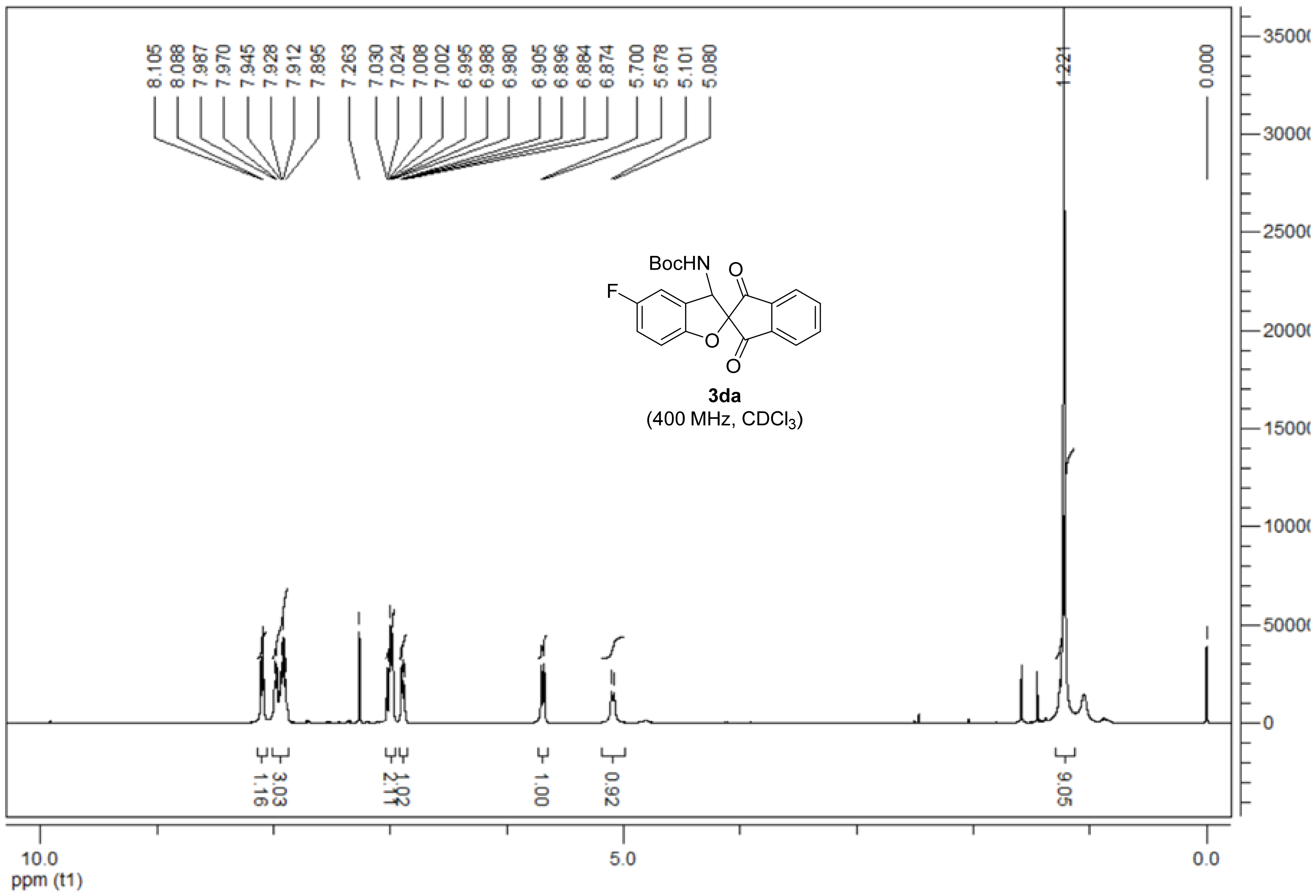
8.105
8.088
7.987
7.970
7.945
7.928
7.912
7.895
7.263
7.030
7.024
7.008
7.002
6.995
6.988
6.980
6.905
6.896
6.884
6.874
5.700
5.678
5.101
5.080
1.221
0.000
BocHN
F
O
O
O
3da
(400 MHz, CDCl3)
1.16
3.03
2.11
1.02
1.00
0.92
9.05
10.0
5.0
0.0
ppm (t1)

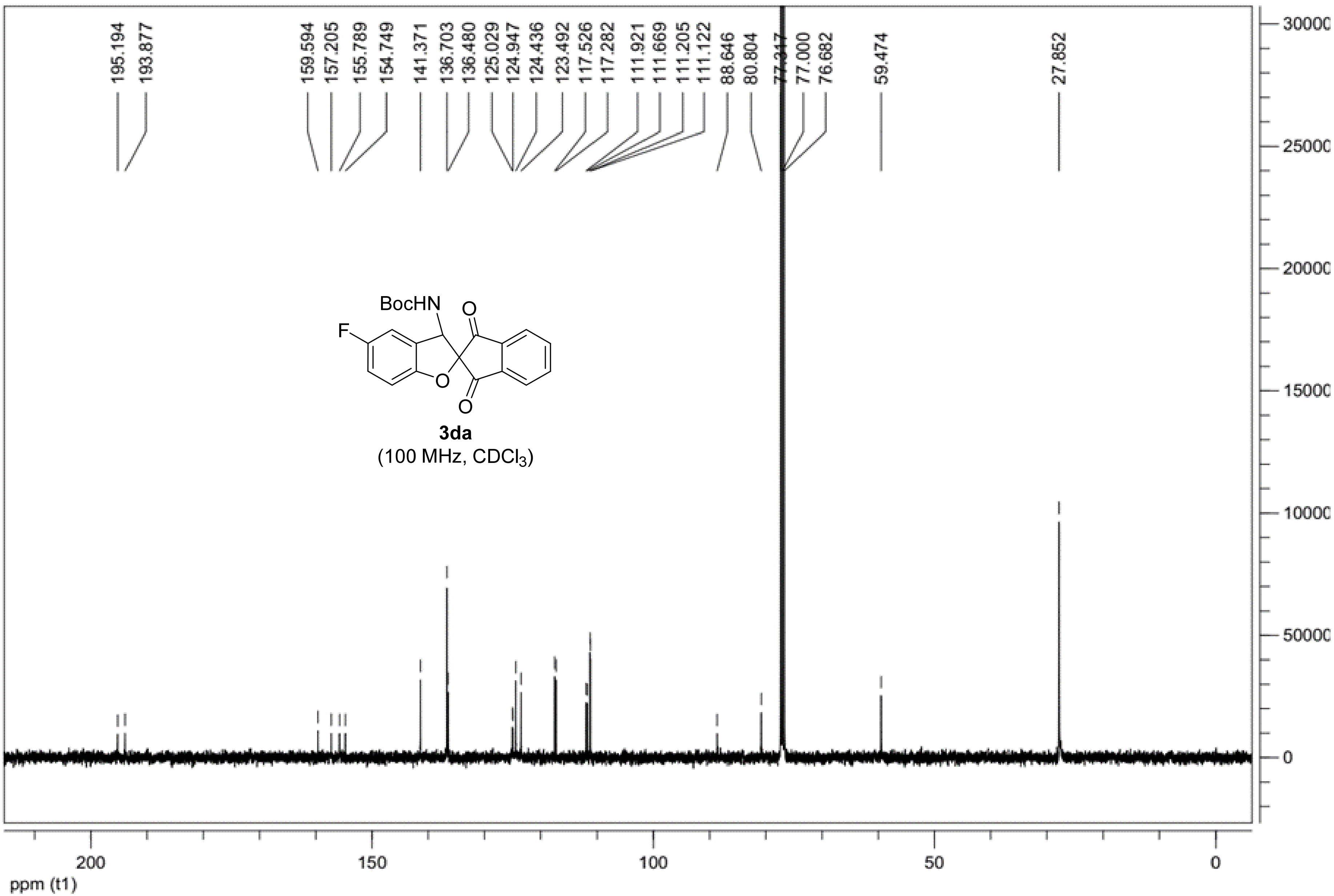
195.194
193.877
159.594
157.205
155.789
154.749
141.371
136.703
136.480
125.029
124.947
124.436
123.492
117.526
117.282
111.921
111.669
111.205
111.122
88.646
80.804
77.317
77.000
76.682
59.474
27.852
BocHN
F
O
O
O
3da
(100 MHz, CDCl3)
ppm (t1)

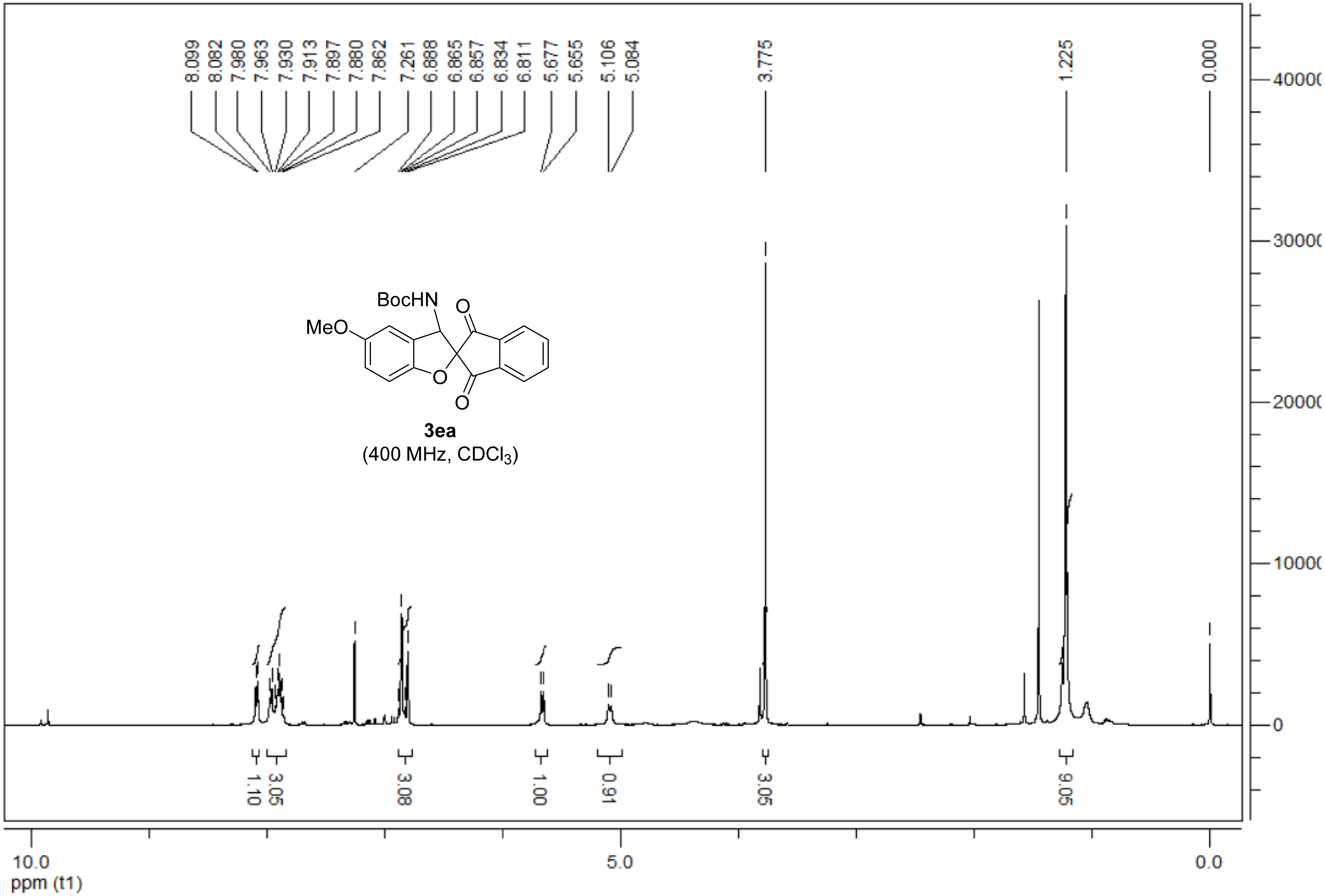

8.099
8.082
7.980
7.963
7.930
7.913
7.897
7.880
7.862
7.261
6.888
6.865
6.857
6.834
6.811
5.677
5.655
5.106
5.084
3.775
1.225
0.000
BocHN
MeO
O
O
O
3ea
(400 MHz, CDCl$_3$)
1.10
3.05
3.08
1.00
0.91
3.05
9.05
10.0
ppm (t1)
5.0
0.0
0
10000
20000
30000
40000

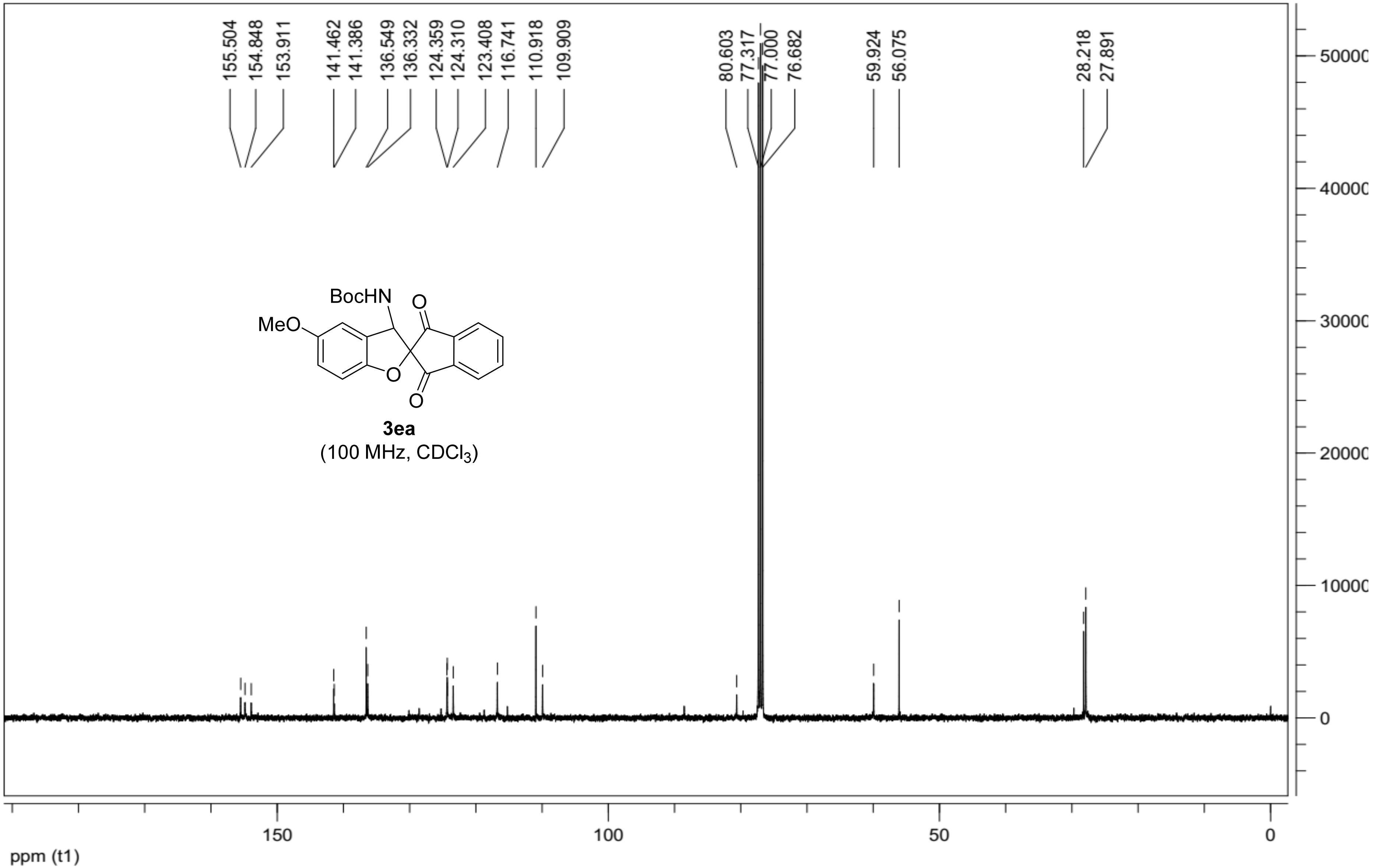
155.504
154.848
153.911
141.462
141.386
136.549
136.332
124.359
124.310
123.408
116.741
110.918
109.909
80.603
77.317
77.000
76.682
59.924
56.075
28.218
27.891
BocHN
MeO
O
O
O
3ea
(100 MHz, CDCl3)
50000
40000
30000
20000
10000
0
150
100
50
0
ppm (t1)

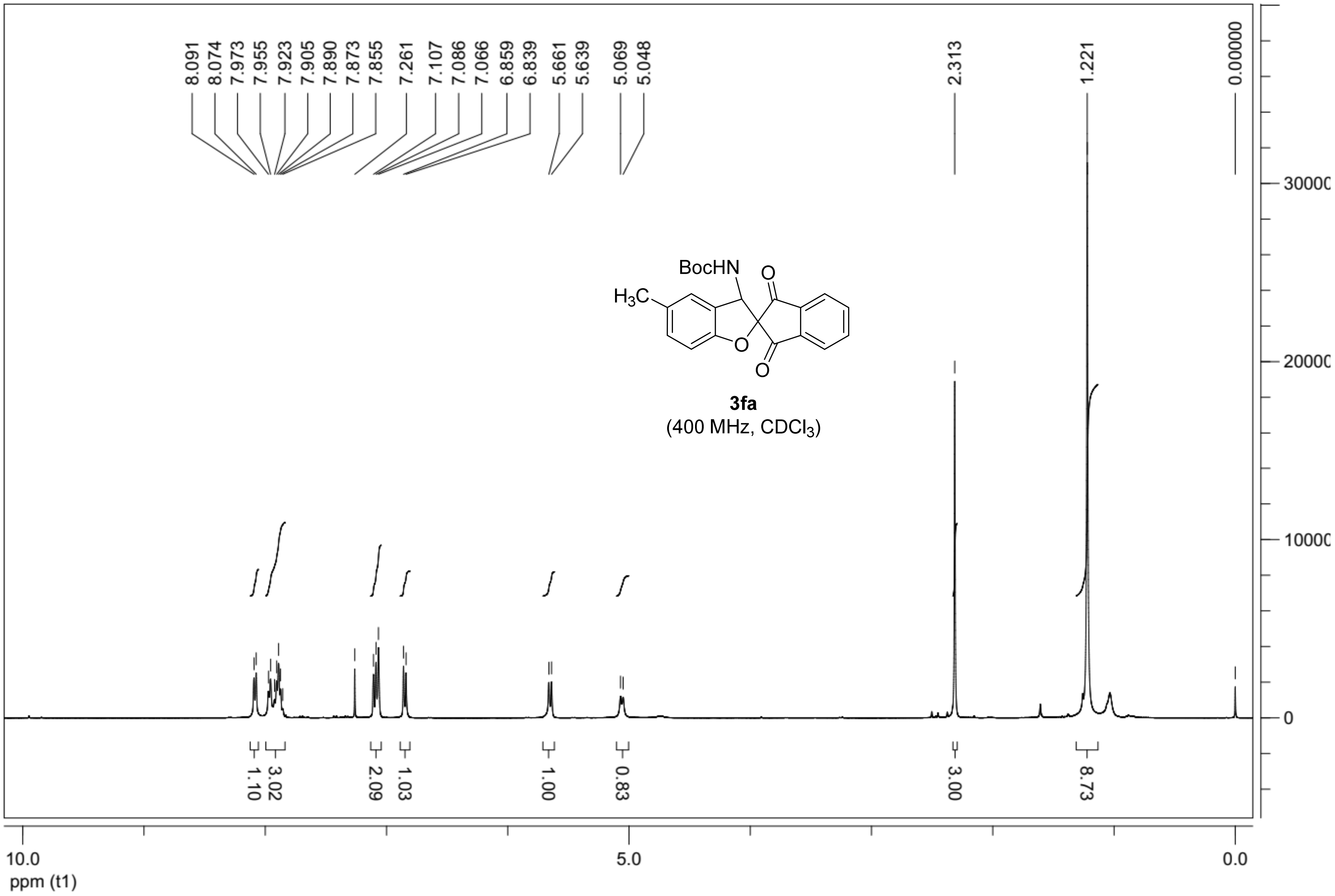

8.091 8.074 7.973 7.955 7.923 7.905 7.890 7.873 7.855 7.261 7.107 7.086 7.066 6.859 6.839 5.661 5.639 5.069 5.048
2.313
1.221
0.00000
BocHN
H3C
O
O
O
3fa
(400 MHz, CDCl3)
1.10 3.02 2.09 1.03 1.00 0.83 3.00 8.73
30000
20000
10000
0
10.0
5.0
0.0
ppm (t1)

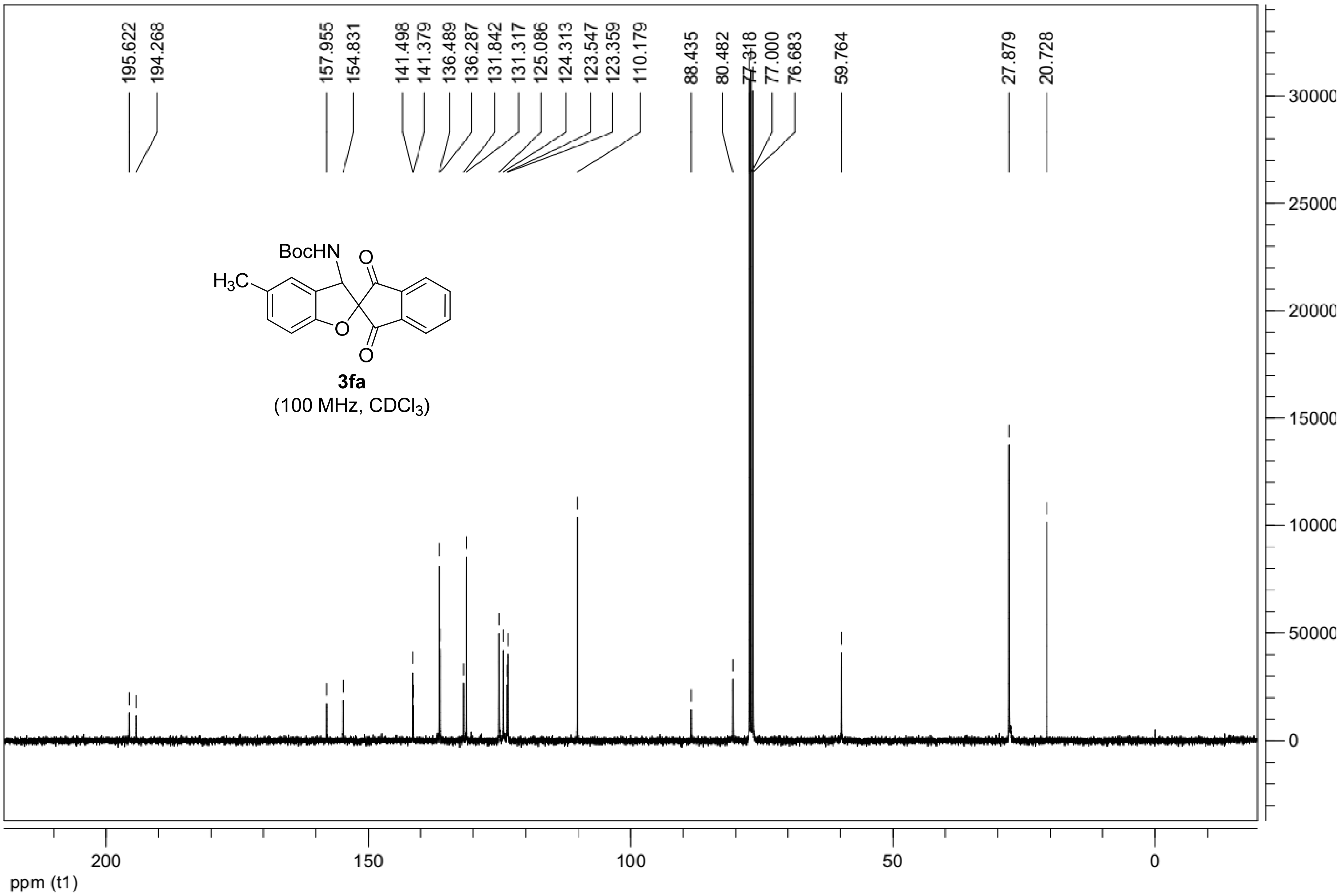

195.622
194.268
157.955
154.831
141.498
141.379
136.489
136.287
131.842
131.317
125.086
124.313
123.547
123.359
110.179
88.435
80.482
77.318
77.000
76.683
59.764
27.879
20.728
BocHN
O
H_3C
O
O
3fa
(100 MHz, $CDCl_3$)
30000
25000
20000
15000
10000
50000
0
200
150
100
50
0
ppm (t1)

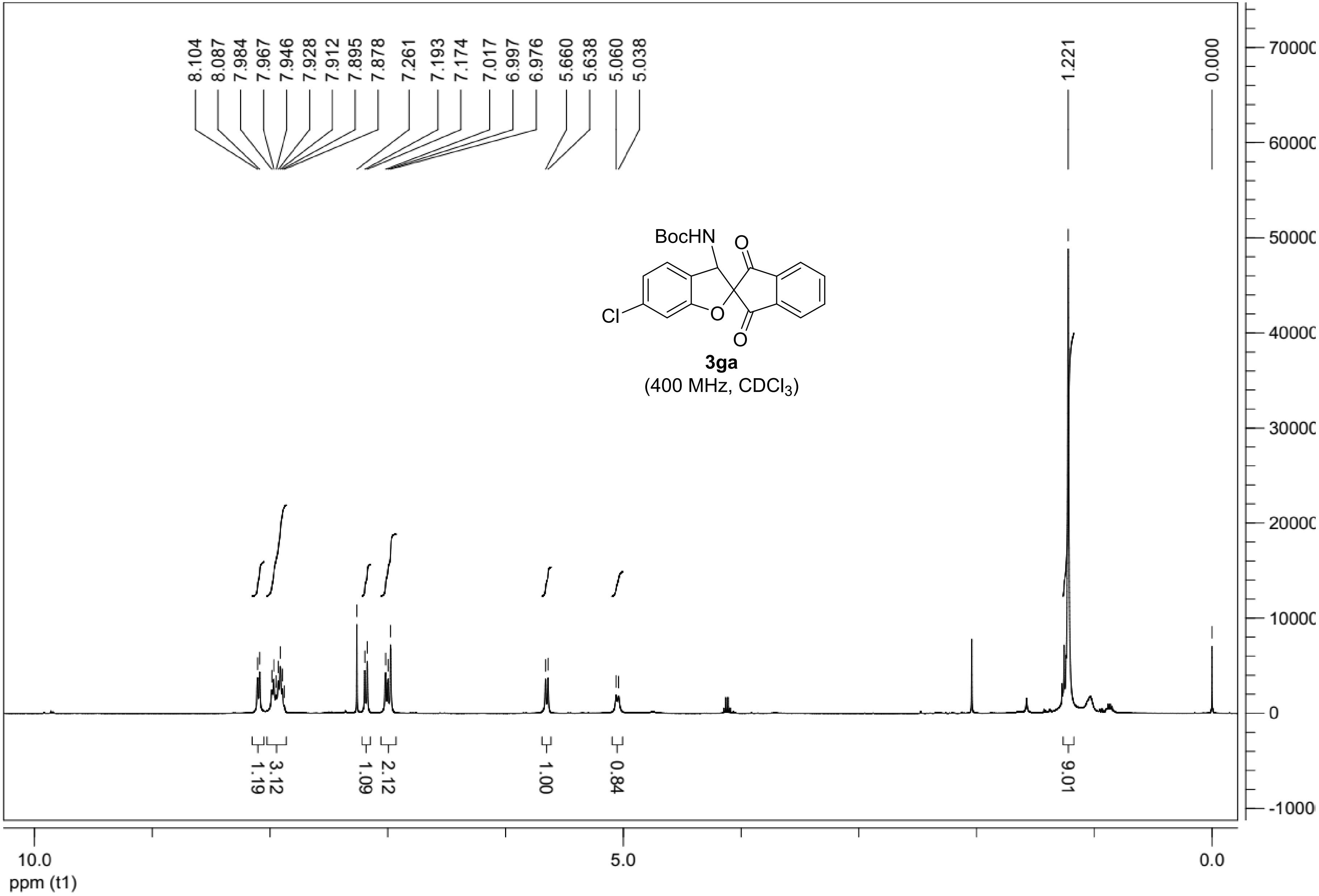

8.104
8.087
7.984
7.967
7.946
7.928
7.912
7.895
7.878
7.261
7.193
7.174
7.017
6.997
6.976
5.660
5.638
5.060
5.038
1.221
0.000
BocHN
O
O
O
Cl
3ga
(400 MHz, CDCl3)
1.19
3.12
1.09
2.12
1.00
0.84
9.01
70000
60000
50000
40000
30000
20000
10000
0
-1000
10.0
5.0
0.0
ppm (t1)

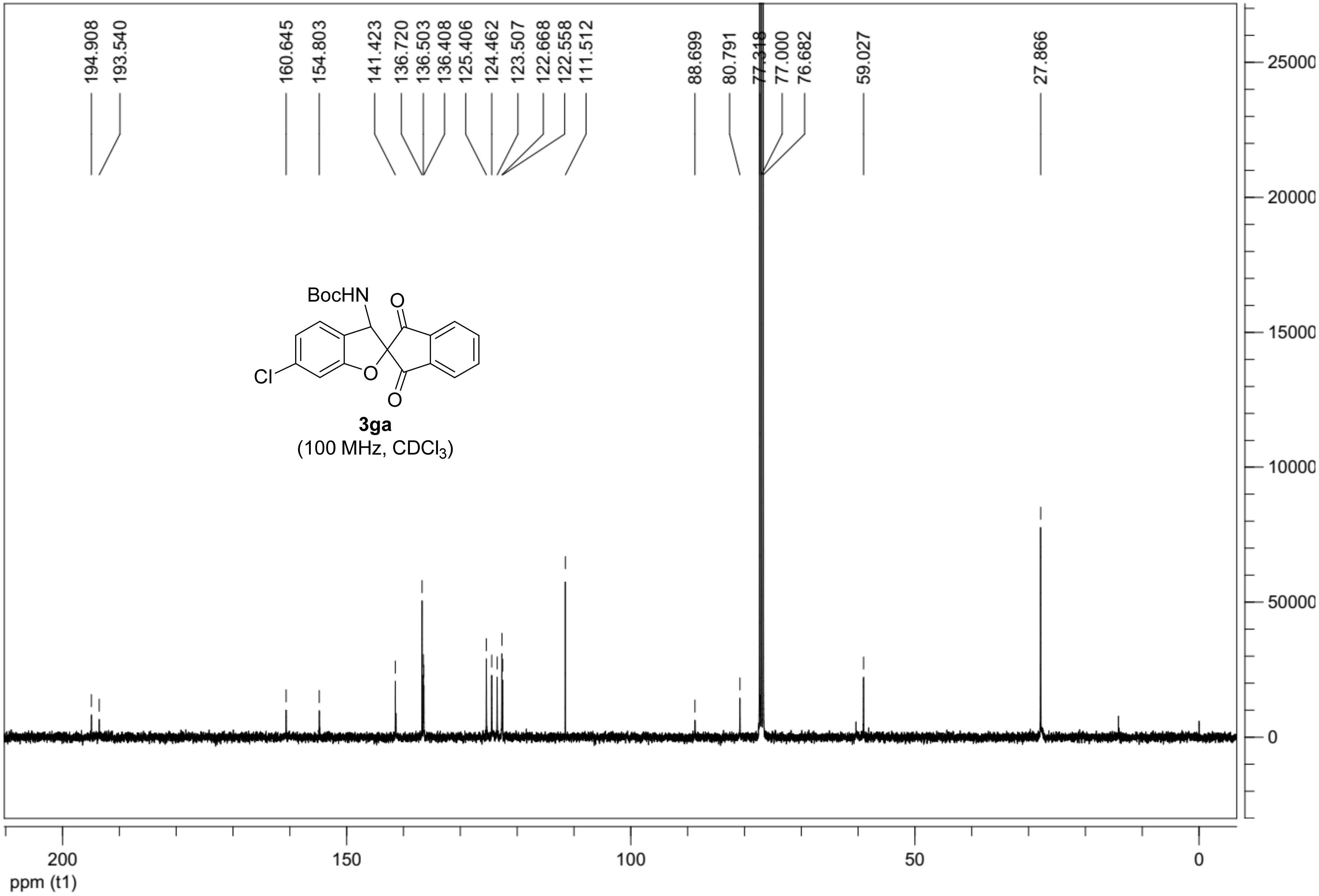
194.908
193.540
160.645
154.803
141.423
136.720
136.503
136.408
125.406
124.462
123.507
122.668
122.558
111.512
88.699
80.791
77.318
77.000
76.682
59.027
27.866
BocHN
O
Cl
O
O
3ga
(100 MHz, CDCl3)
25000
20000
15000
10000
50000
0
200
150
100
50
0
ppm (t1)

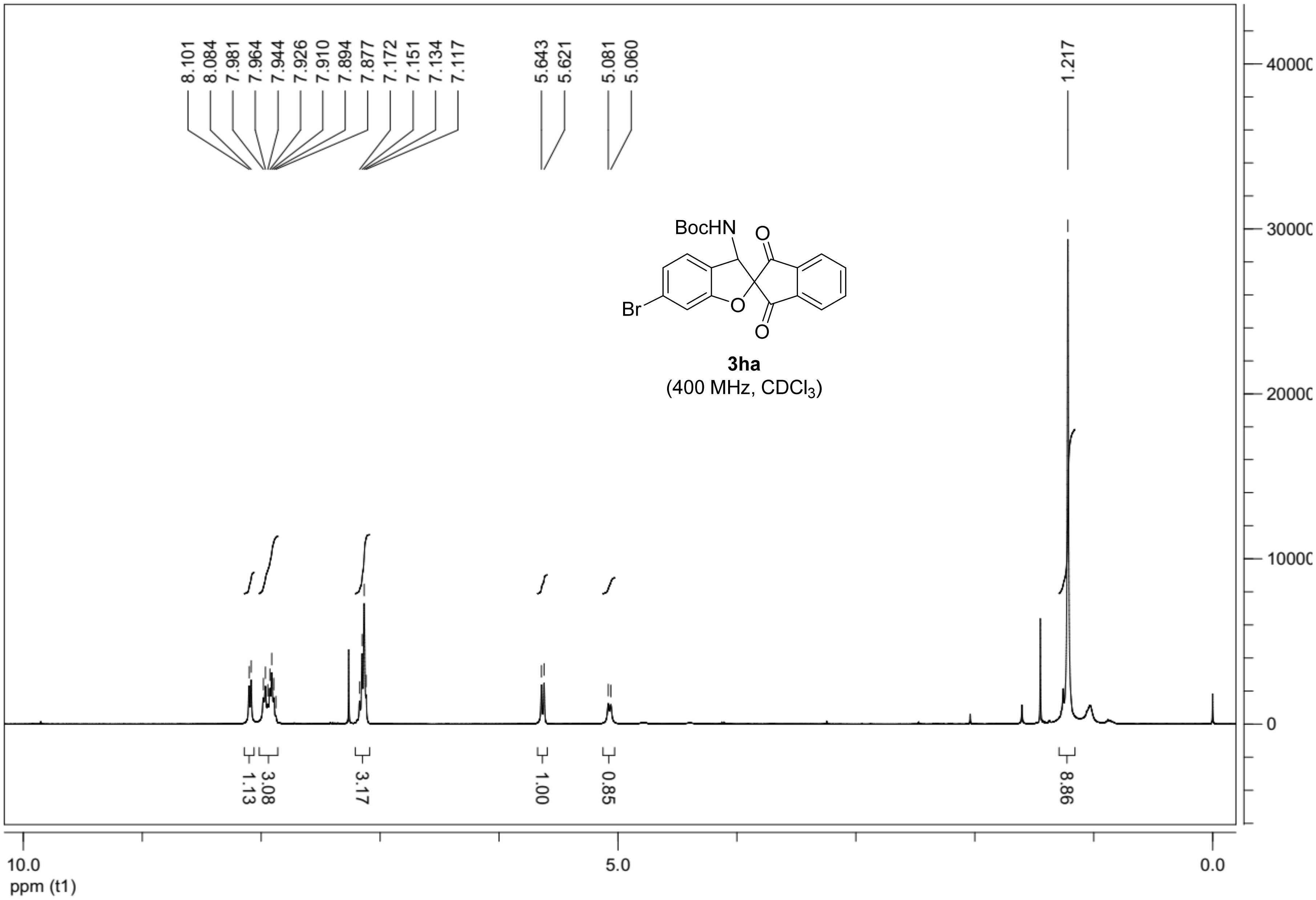
8.101
8.084
7.981
7.964
7.944
7.926
7.910
7.894
7.877
7.172
7.151
7.134
7.117
5.643
5.621
5.081
5.060
1.217
BocHN
O
Br
3ha
(400 MHz, CDCl3)
1.13
3.08
3.17
1.00
0.85
8.86
40000
30000
20000
10000
0
10.0
5.0
0.0
ppm (t1)

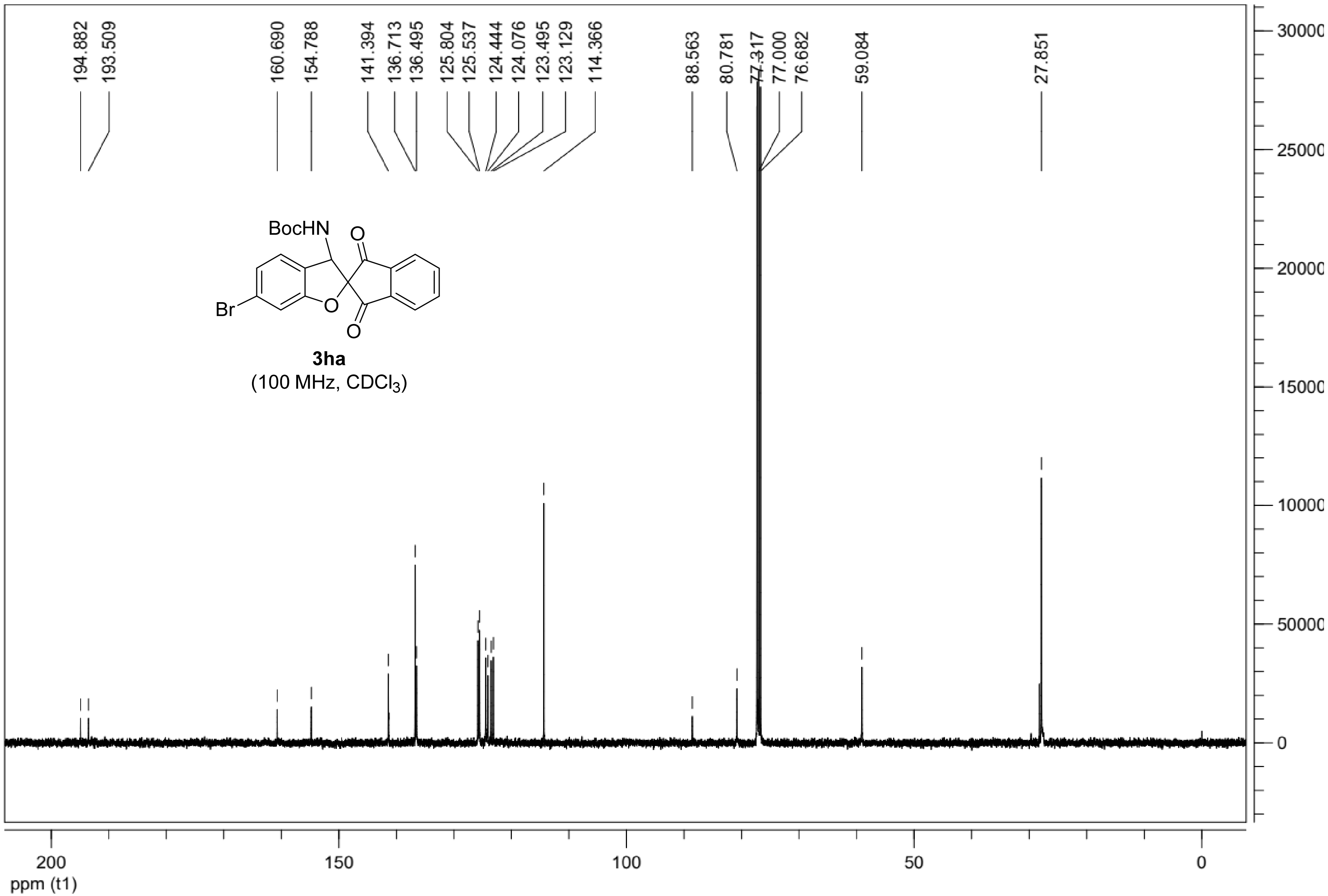
194.882
193.509
160.690
154.788
141.394
136.713
136.495
125.804
125.537
124.444
124.076
123.495
123.129
114.366
88.563
80.781
77.317
77.000
76.682
59.084
27.851
BocHN
Br
O
3ha
(100 MHz, CDCl3)
30000
25000
20000
15000
10000
50000
0
200
150
100
50
0
ppm (t1)

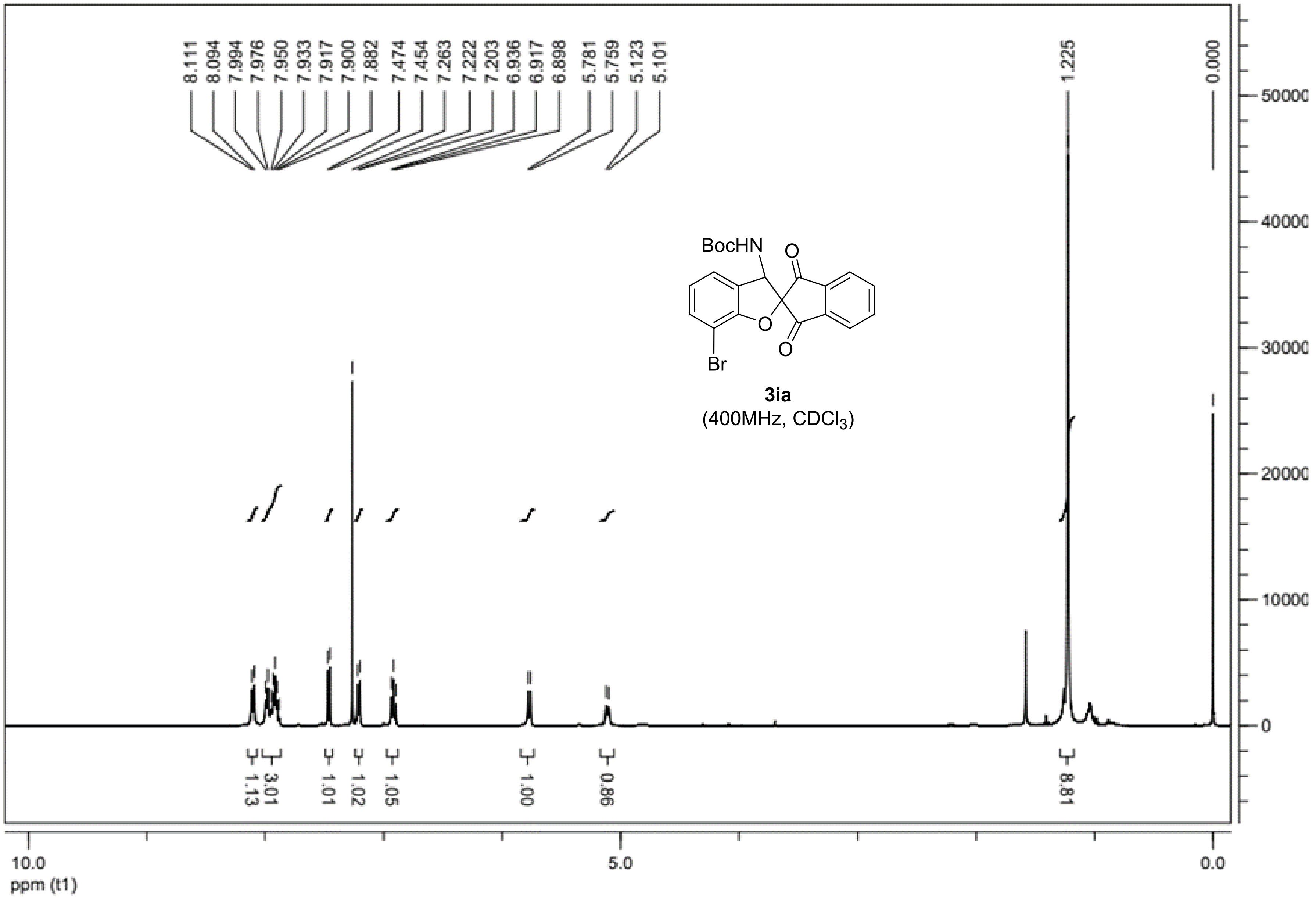
8.111
8.094
7.994
7.976
7.950
7.933
7.917
7.900
7.882
7.474
7.454
7.263
7.222
7.203
6.936
6.917
6.898
5.781
5.759
5.123
5.101
1.225
0.000
BocHN
O
O
O
Br
3ia
(400MHz, CDCl3)
1.13
3.01
1.01
1.02
1.05
1.00
0.86
8.81
10.0
5.0
0.0
ppm (t1)
50000
40000
30000
20000
10000
0

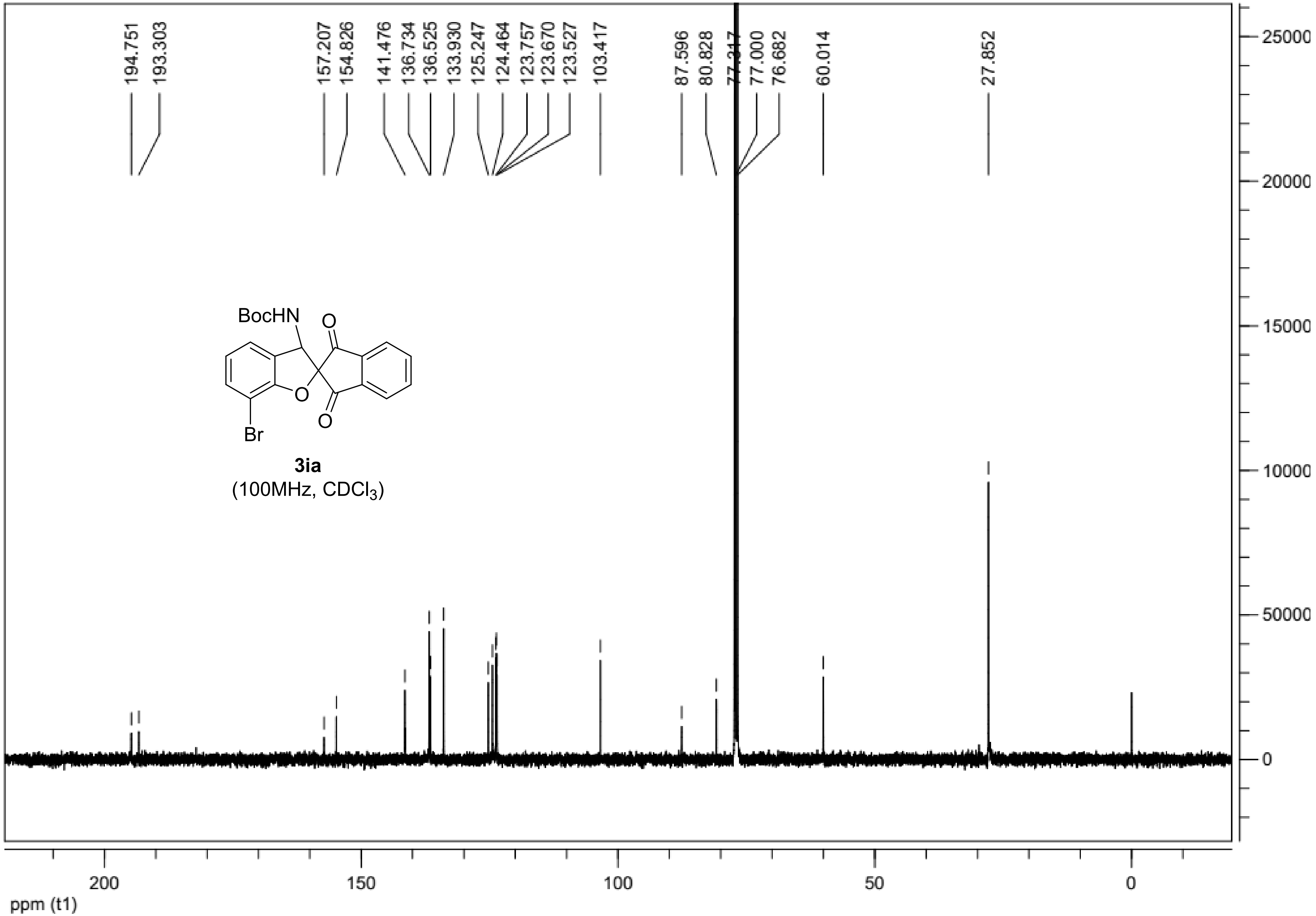
194.751
193.303
157.207
154.826
141.476
136.734
136.525
133.930
125.247
124.464
123.757
123.670
123.527
103.417
87.596
80.828
77.317
77.000
76.682
60.014
27.852
BocHN
O
O
O
Br
3ia
(100MHz, CDCl3)
25000
20000
15000
10000
50000
0
200
150
100
50
0
ppm (t1)

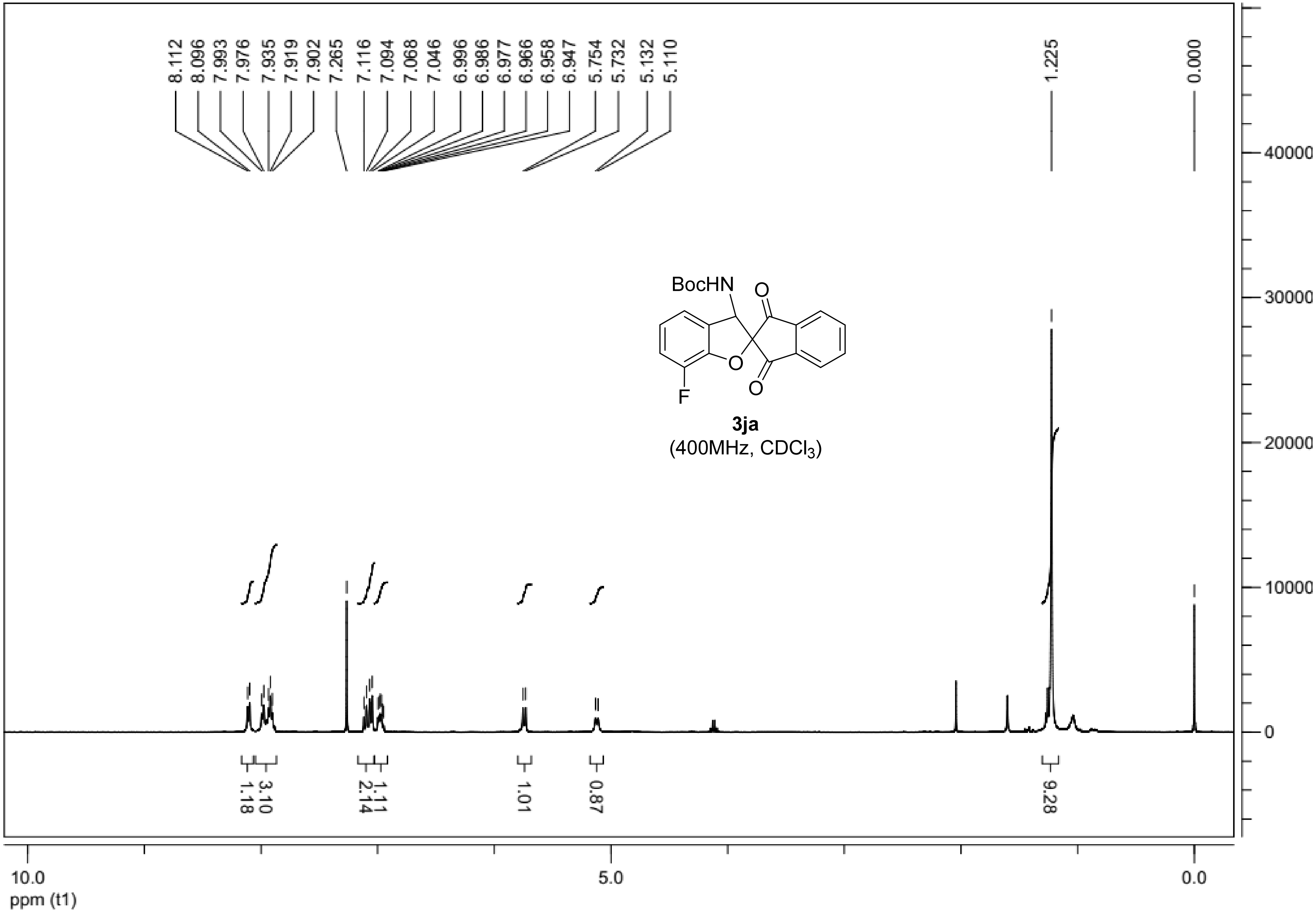

8.112
8.096
7.993
7.976
7.935
7.919
7.902
7.265
7.116
7.094
7.068
7.046
6.996
6.986
6.977
6.966
6.958
6.947
5.754
5.732
5.132
5.110
1.225
0.000
BocHN
O
O
O
F
3ja
(400MHz, $CDCl_3$)
40000
30000
20000
10000
0
1.18
3.10
2.14
1.11
1.01
0.87
9.28
10.0
5.0
0.0
ppm (t1)

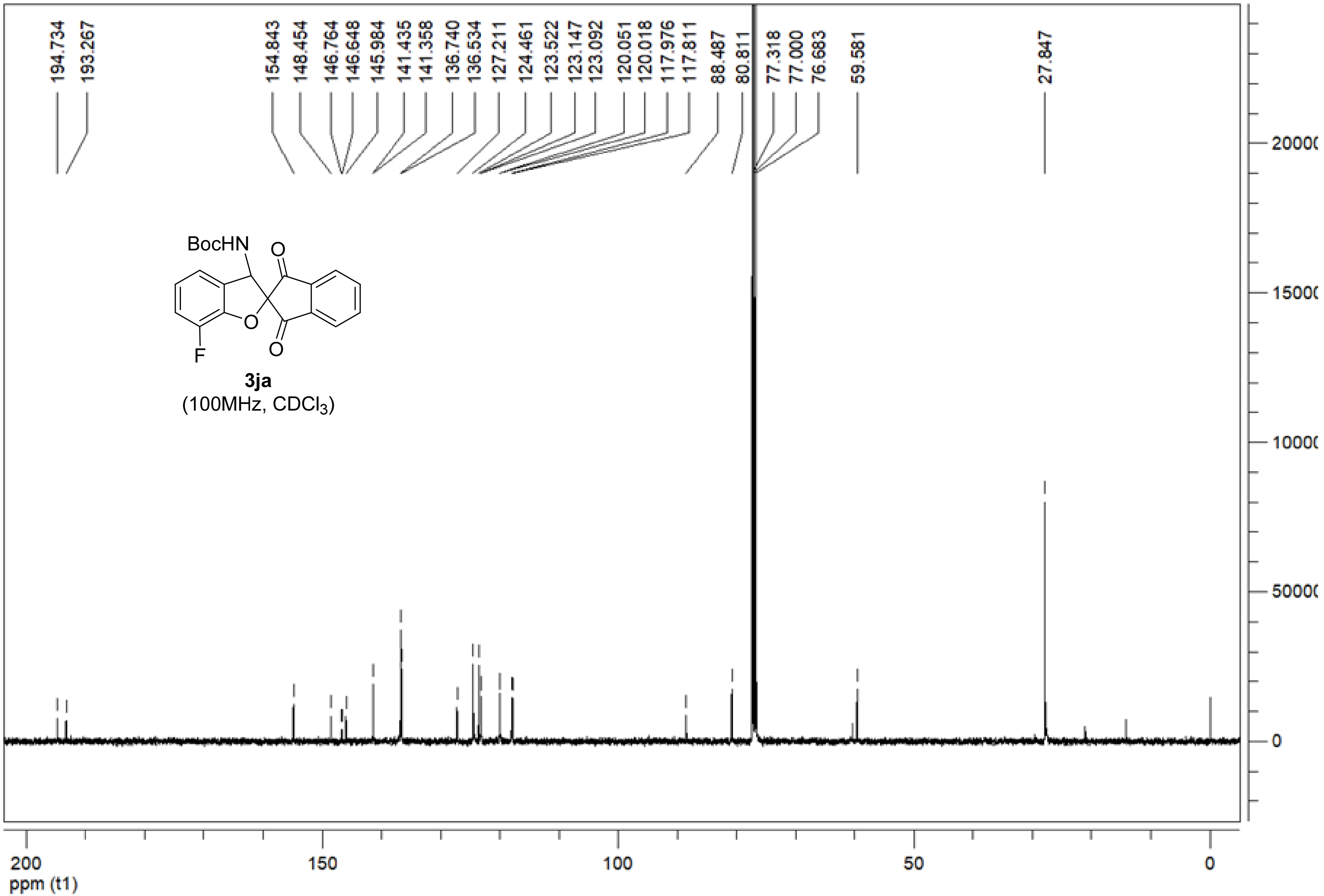
194.734
193.267
154.843
148.454
146.764
146.648
145.984
141.435
141.358
136.740
136.534
127.211
124.461
123.522
123.147
123.092
120.051
120.018
117.976
117.811
88.487
80.811
77.318
77.000
76.683
59.581
27.847
BocHN
O
O
O
F
3ja
(100MHz, CDCl3)
20000
15000
10000
50000
0
200
150
100
50
0
ppm (t1)

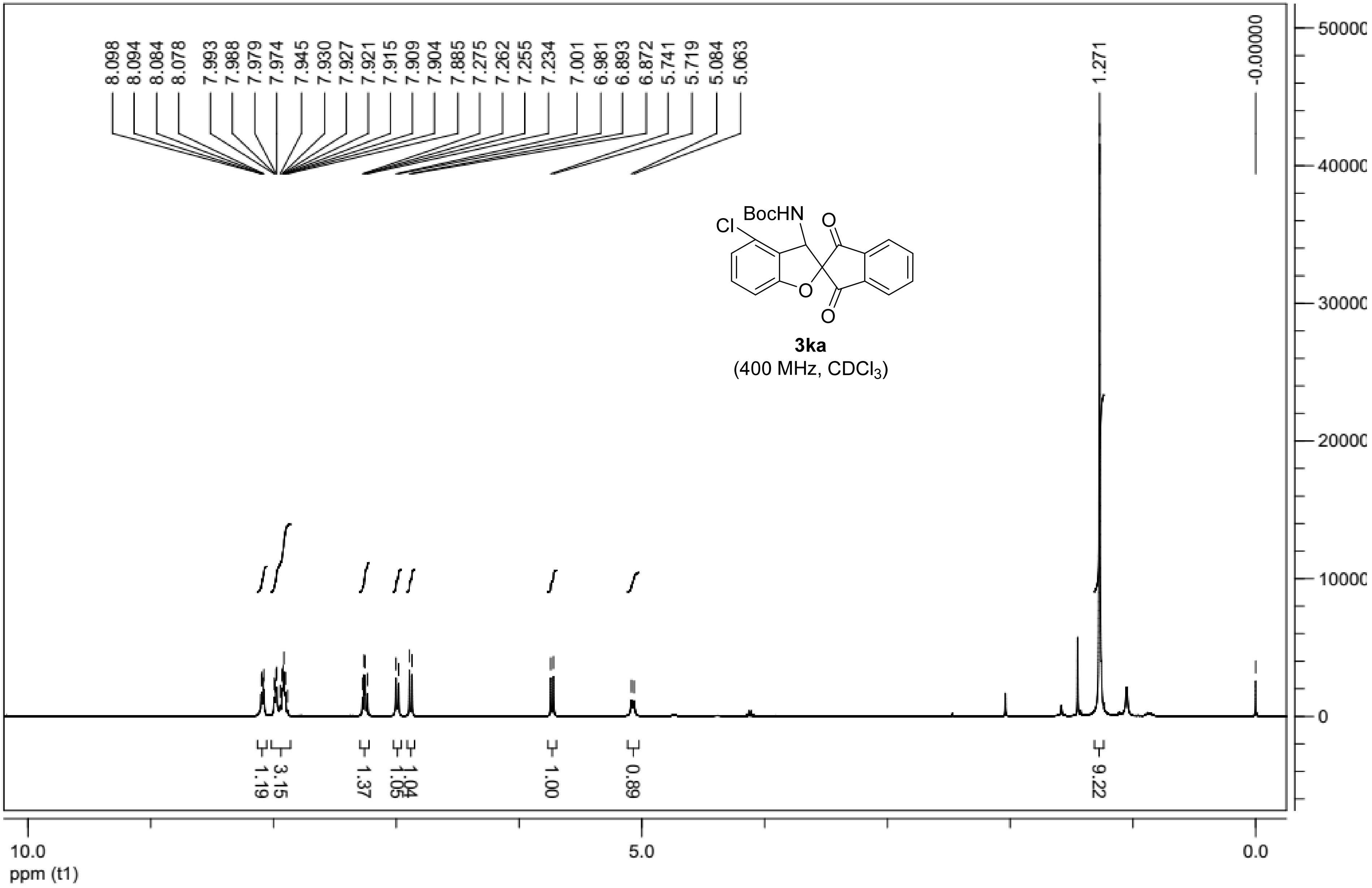
8.098
8.094
8.084
8.078
7.993
7.988
7.979
7.974
7.945
7.930
7.927
7.921
7.915
7.909
7.904
7.885
7.275
7.262
7.255
7.234
7.001
6.981
6.893
6.872
5.741
5.719
5.084
5.063
1.271
-0.00000
Cl
BocHN
O
O
O
3ka
(400 MHz, CDCl3)
1.19
3.15
1.37
1.05
1.04
1.00
0.89
9.22
10.0
5.0
0.0
ppm (t1)
50000
40000
30000
20000
10000
0

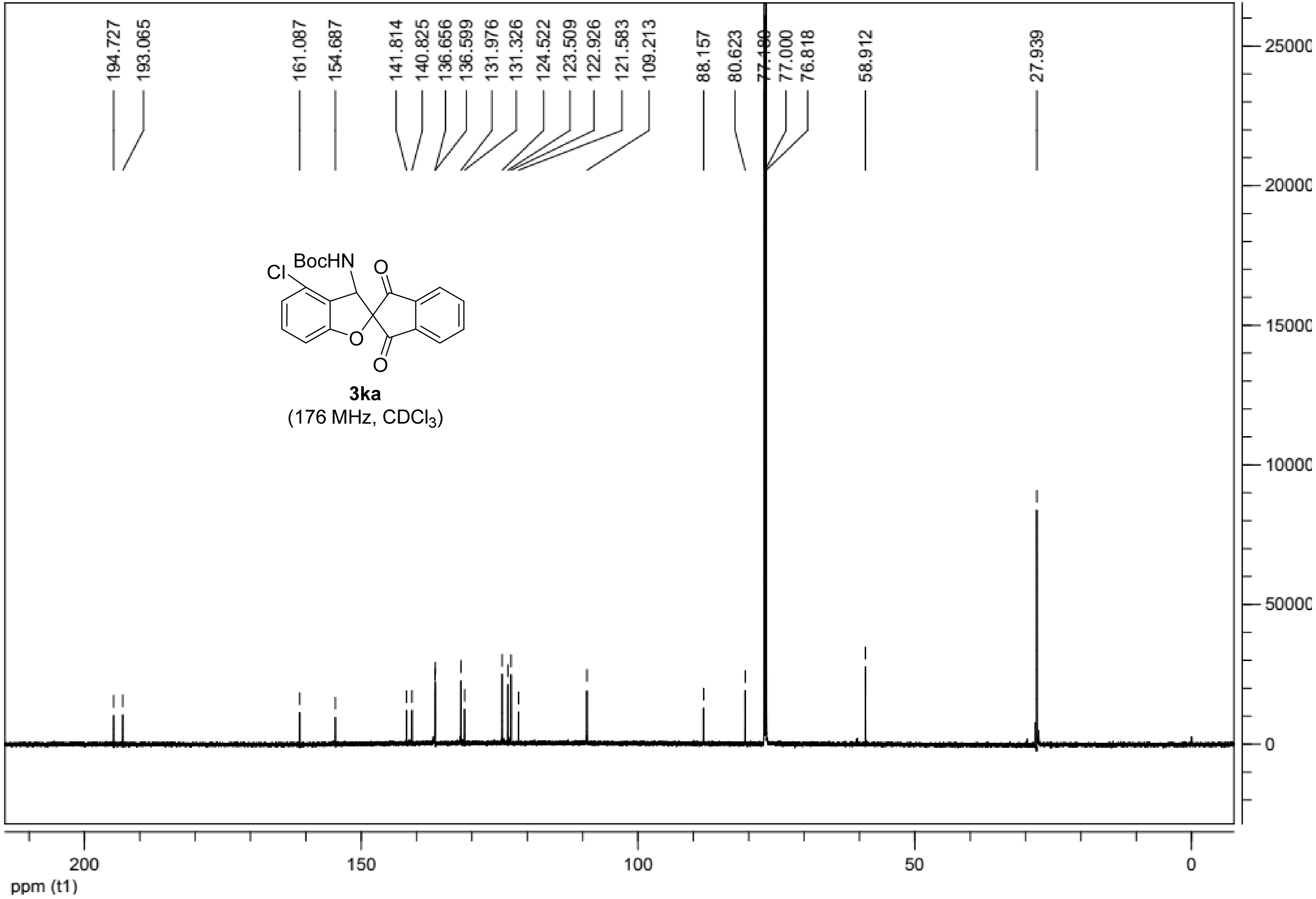
194.727
193.065
161.087
154.687
141.814
140.825
136.656
136.599
131.976
131.326
124.522
123.509
122.926
121.583
109.213
88.157
80.623
77.180
77.000
76.818
58.912
27.939
Cl
BocHN
O
O
O
3ka
(176 MHz, CDCl3)
25000
20000
15000
10000
50000
0
200
150
100
50
0
ppm (t1)

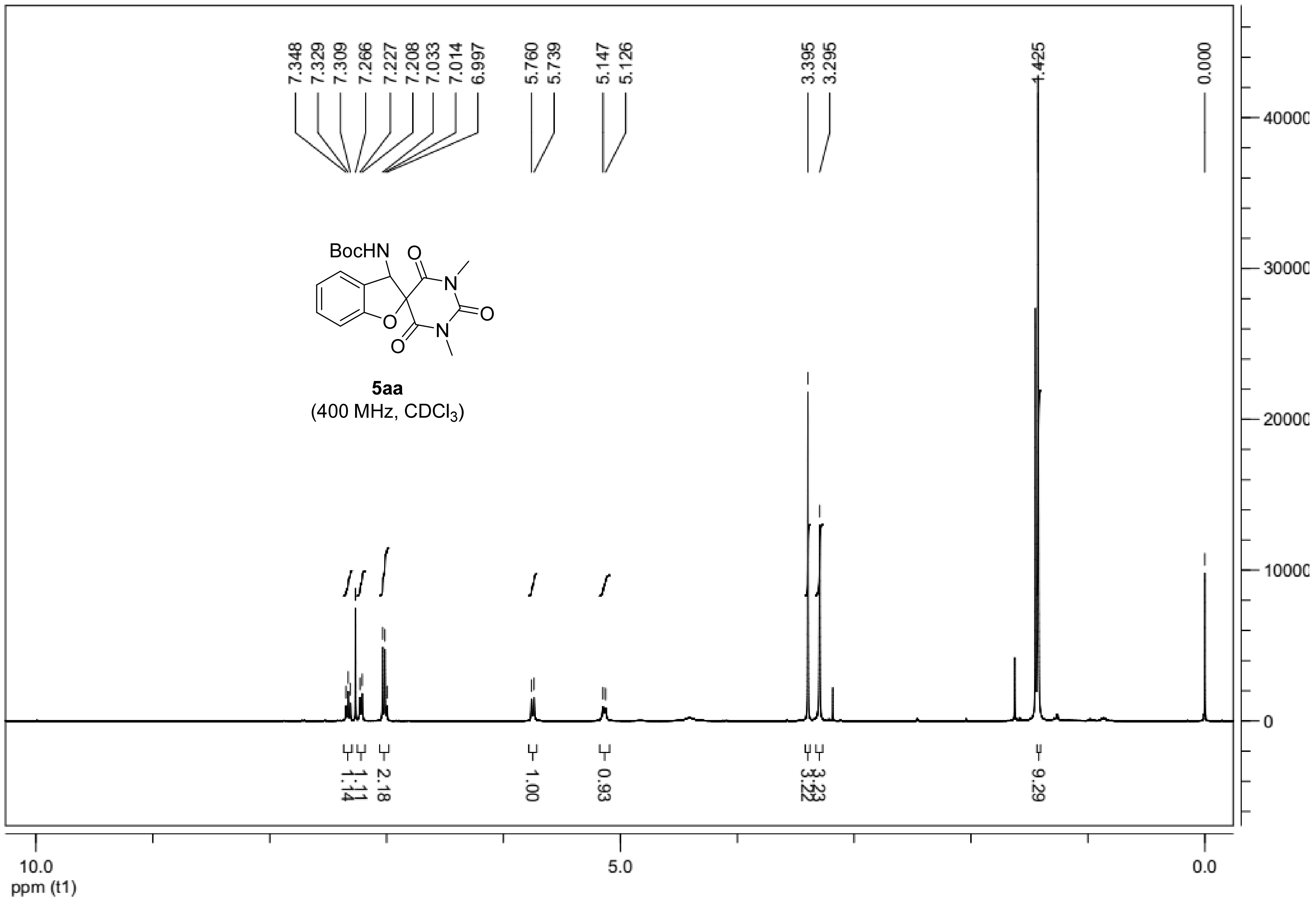
7.348
7.329
7.309
7.266
7.227
7.208
7.033
7.014
6.997
5.760
5.739
5.147
5.126
3.395
3.295
1.425
0.000
BocHN
O
N
O
O
N
O
5aa
(400 MHz, CDCl3)
40000
30000
20000
10000
0
1.14
1.11
2.18
1.00
0.93
3.22
3.23
9.29
10.0
ppm (t1)
5.0
0.0

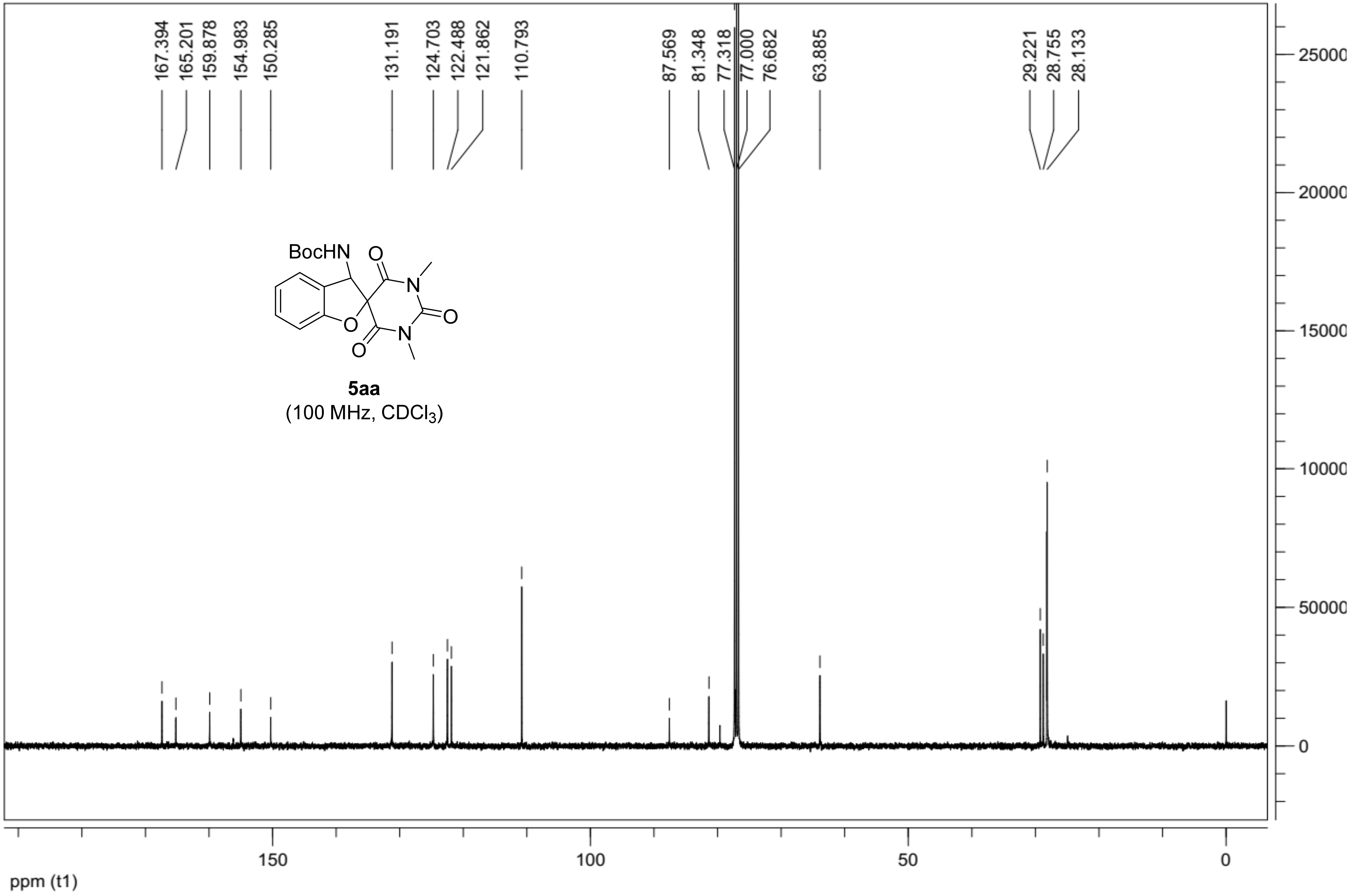
167.394
165.201
159.878
154.983
150.285
131.191
124.703
122.488
121.862
110.793
87.569
81.348
77.318
77.000
76.682
63.885
29.221
28.755
28.133
BocHN
O
N
O
O
N
O
5aa
(100 MHz, $CDCl_3$)
25000
20000
15000
10000
50000
0
150
100
50
0
ppm (t1)

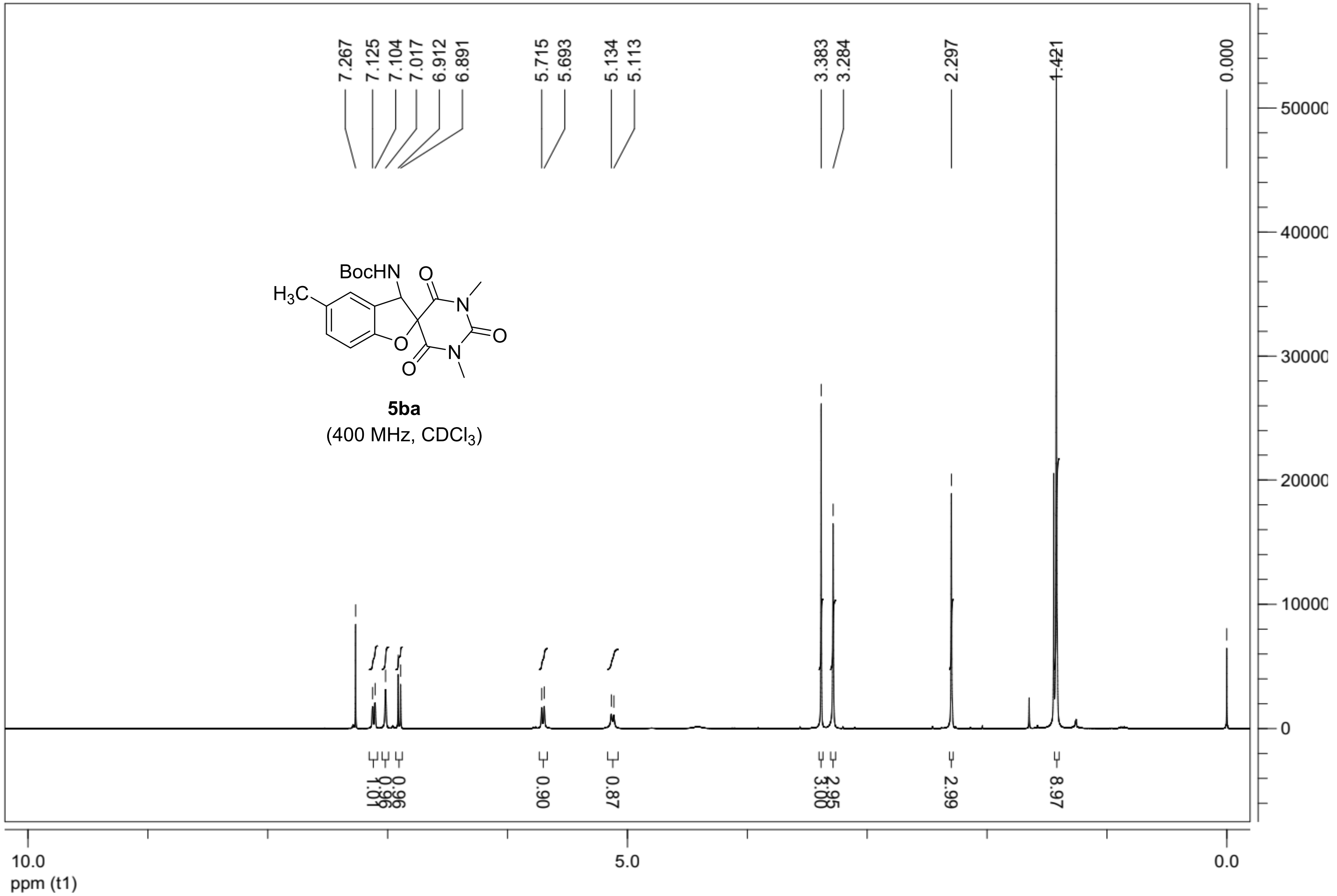
7.267
7.125
7.104
7.017
6.912
6.891
5.715
5.693
5.134
5.113
3.383
3.284
2.297
1.421
0.000
BocHN
H3C
O
N
5ba
(400 MHz, CDCl3)
50000
40000
30000
20000
10000
0
1.01
0.96
0.96
0.90
0.87
3.00
2.95
2.99
8.97
10.0
5.0
0.0
ppm (t1)

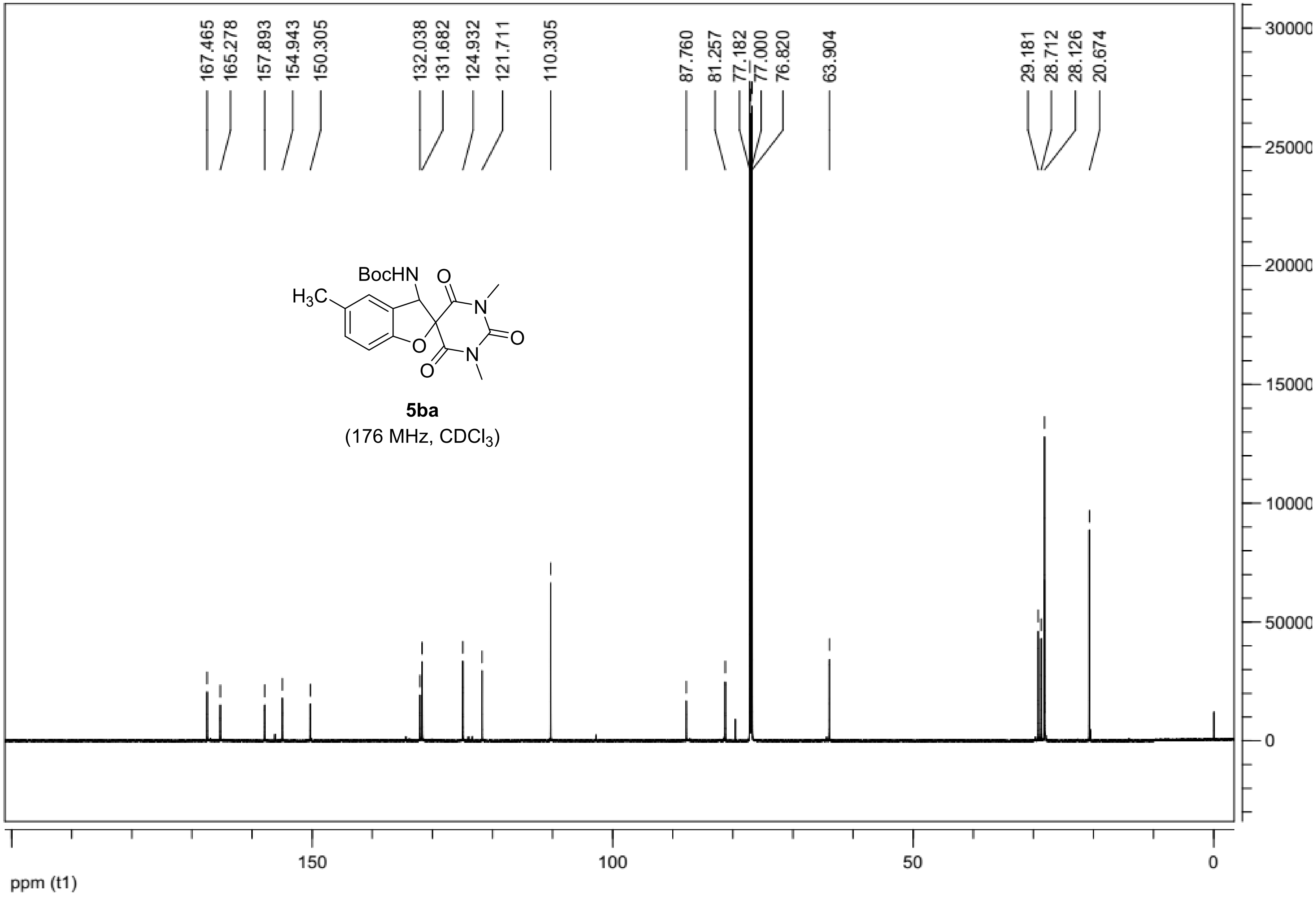

167.465
165.278
157.893
154.943
150.305
132.038
131.682
124.932
121.711
110.305
87.760
81.257
77.182
77.000
76.820
63.904
29.181
28.712
28.126
20.674
BocHN
H_3C
O
N
O
N
O
O
5ba
(176 MHz, $CDCl_3$)
30000
25000
20000
15000
10000
50000
0
150
100
50
0
ppm (t1)

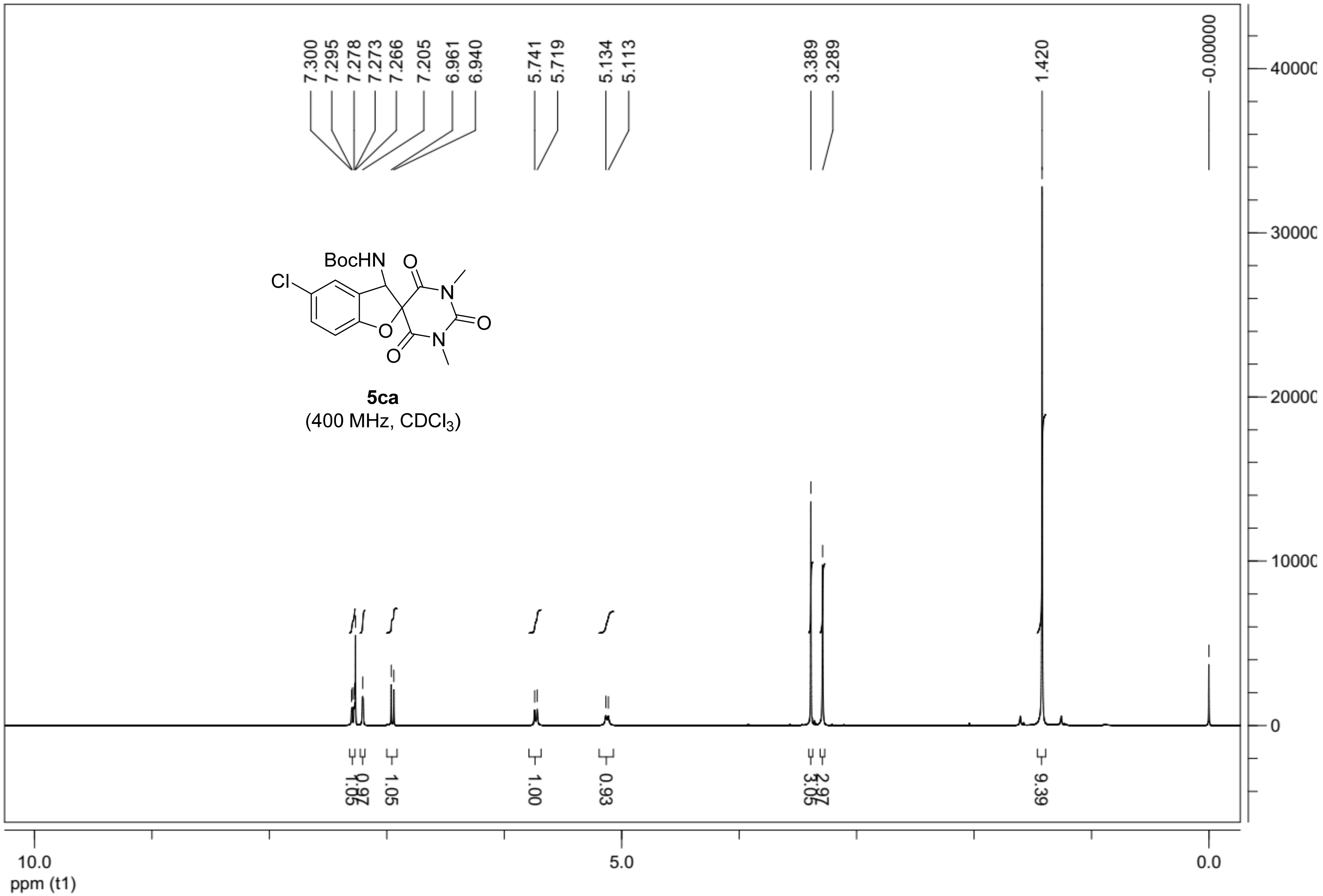

5ca
(400 MHz, $CDCl_3$)

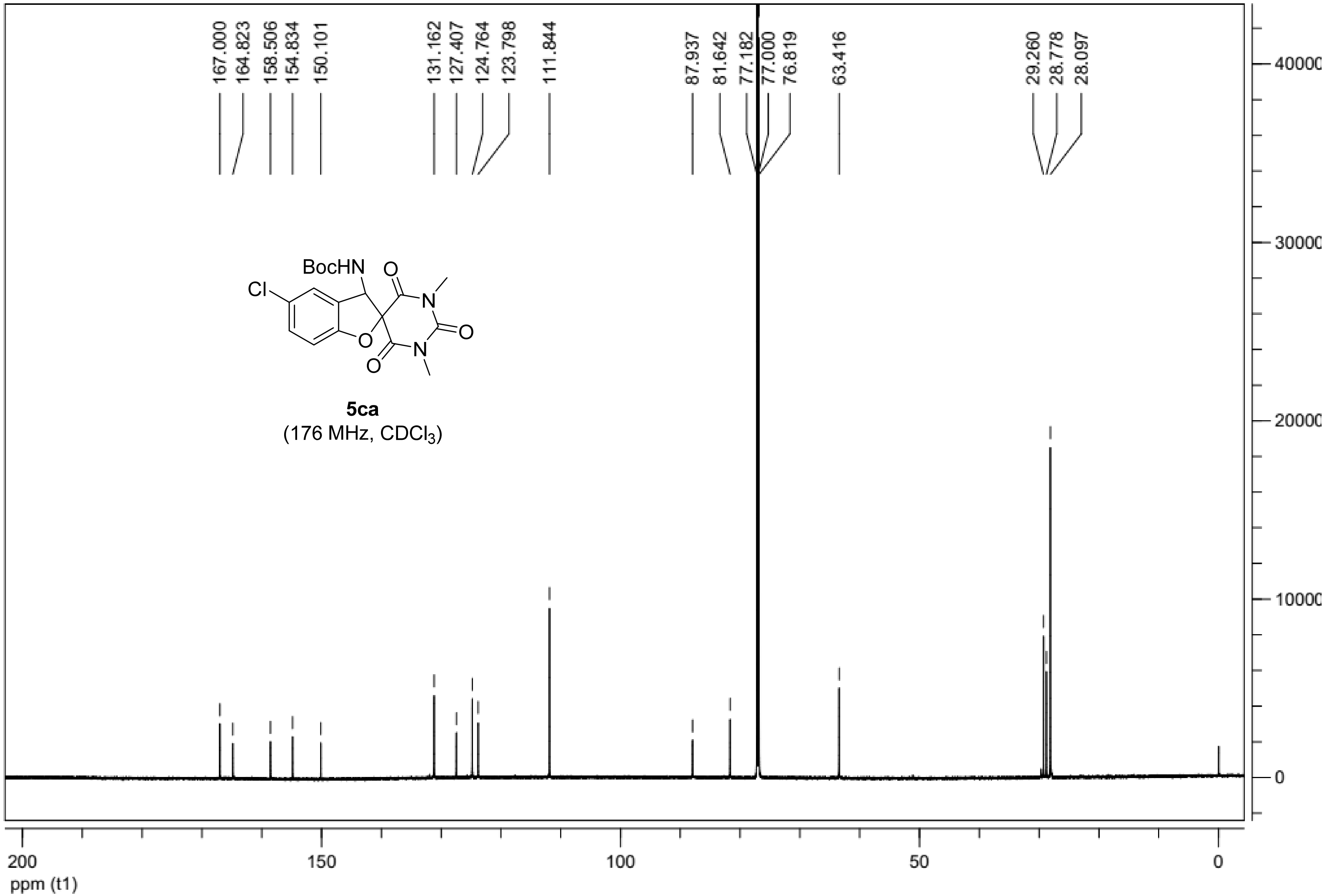
167.000
164.823
158.506
154.834
150.101
131.162
127.407
124.764
123.798
111.844
87.937
81.642
77.182
77.000
76.819
63.416
29.260
28.778
28.097
BocHN
Cl
O
N
5ca
(176 MHz, CDCl3)
40000
30000
20000
10000
0
200
150
100
50
0
ppm (t1)

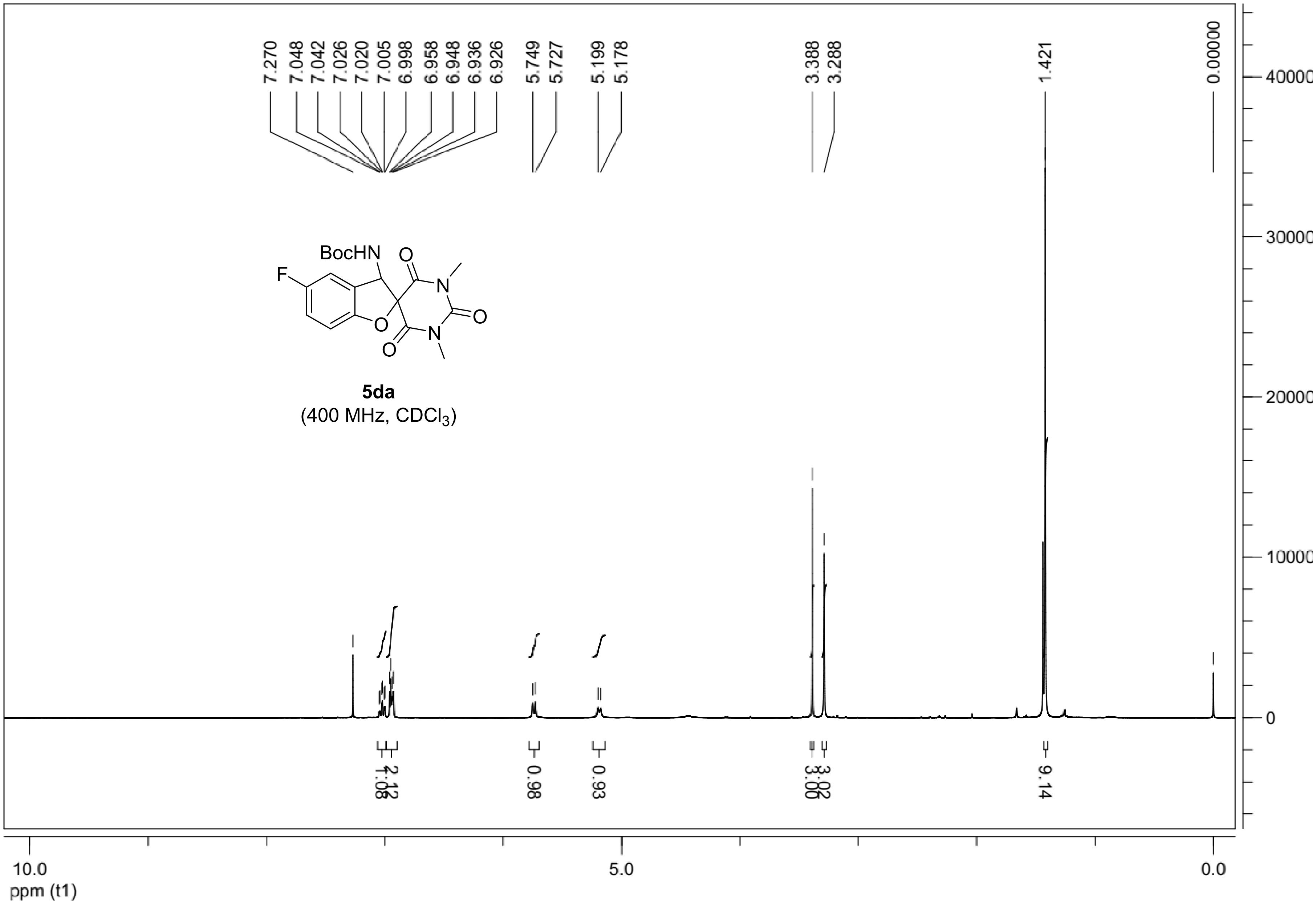
7.270
7.048
7.042
7.026
7.020
7.005
6.998
6.958
6.948
6.936
6.926
5.749
5.727
5.199
5.178
3.388
3.288
1.421
0.00000
BocHN
F
O
N
O
O
N
O
5da
(400 MHz, CDCl3)
1.08
2.12
0.98
0.93
3.06
3.02
9.14
40000
30000
20000
10000
0
10.0
5.0
0.0
ppm (t1)

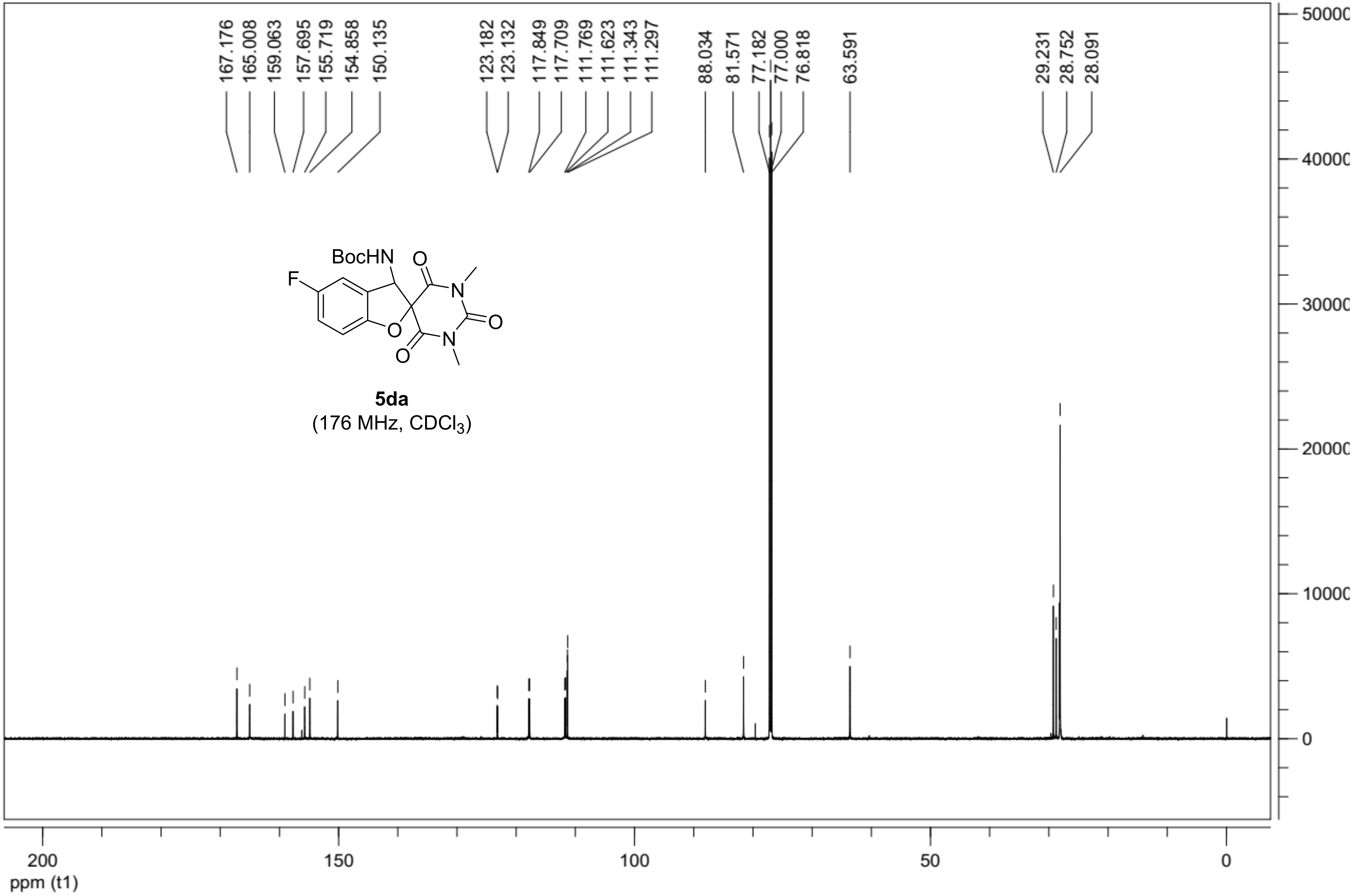

167.176
165.008
159.063
157.695
155.719
154.858
150.135
123.182
123.132
117.849
117.709
111.769
111.623
111.343
111.297
88.034
81.571
77.182
77.000
76.818
63.591
29.231
28.752
28.091
BocHN
F
O
N
5da
(176 MHz, CDCl3)
50000
40000
30000
20000
10000
0
200
150
100
50
0
ppm (t1)

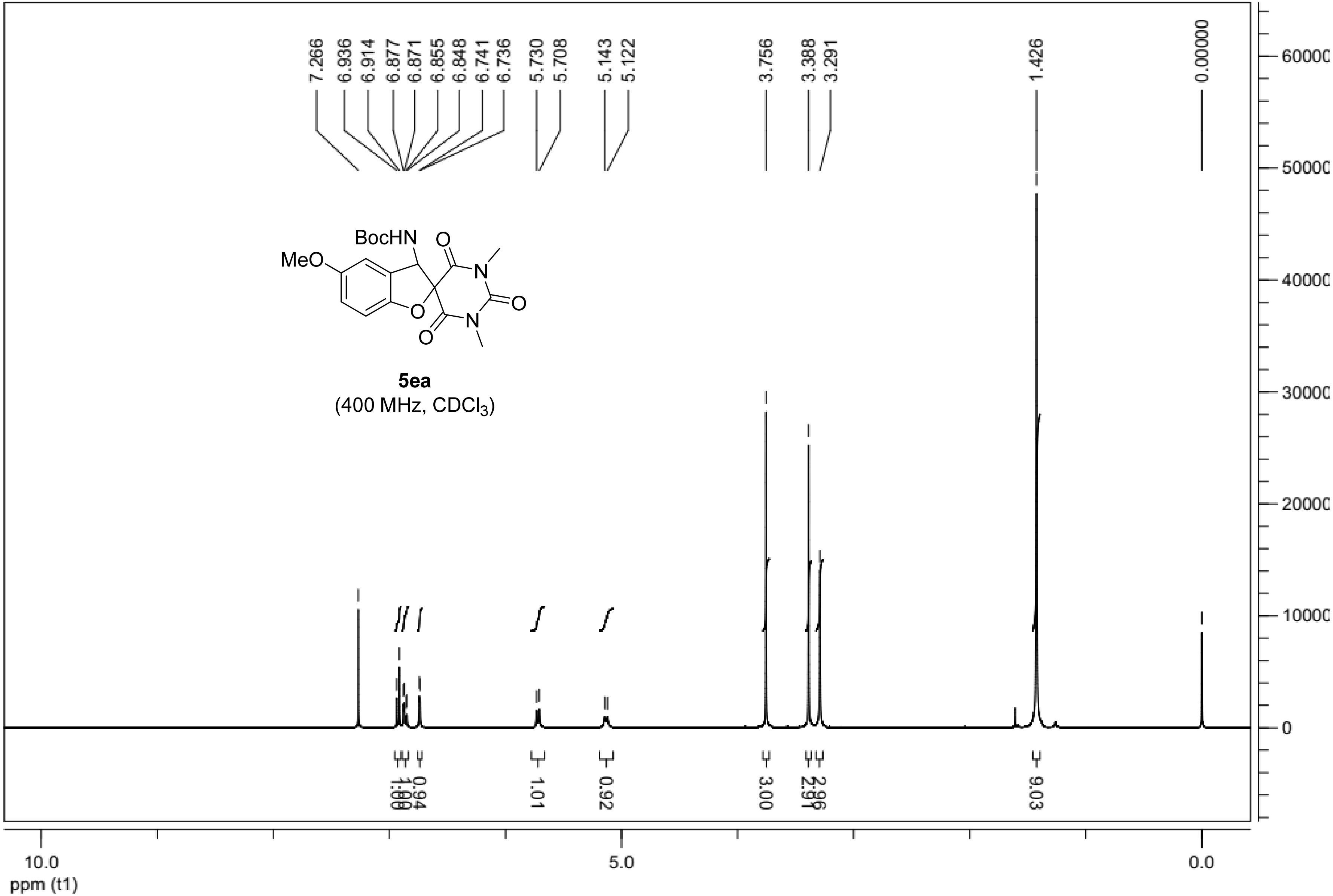

5ea
(400 MHz, $CDCl_3$)

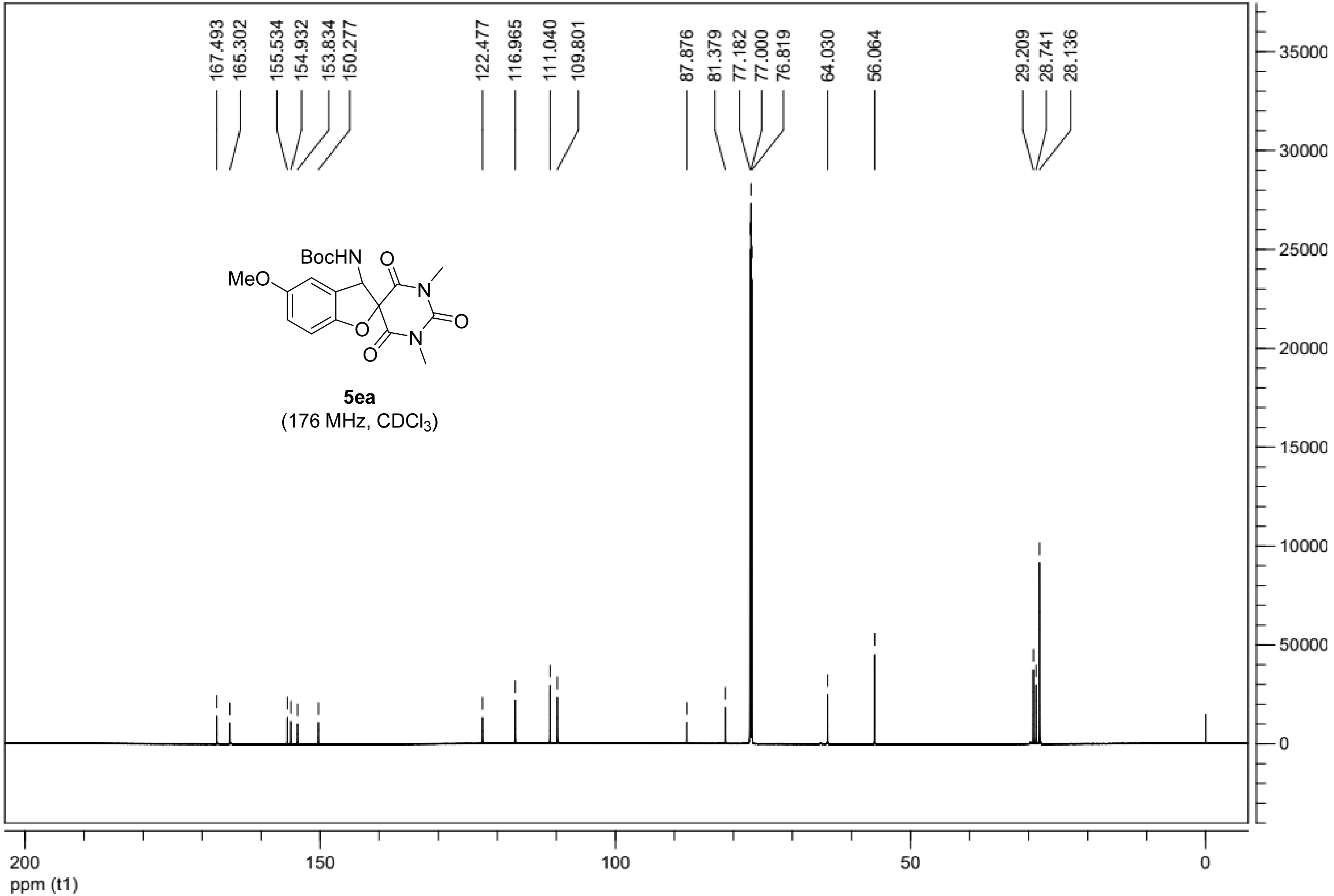
167.493
165.302
155.534
154.932
153.834
150.277
122.477
116.965
111.040
109.801
87.876
81.379
77.182
77.000
76.819
64.030
56.064
29.209
28.741
28.136
BocHN
MeO
O
N
5ea
(176 MHz, $CDCl_3$)
35000
30000
25000
20000
15000
10000
50000
0
200
150
100
50
0
ppm (t1)

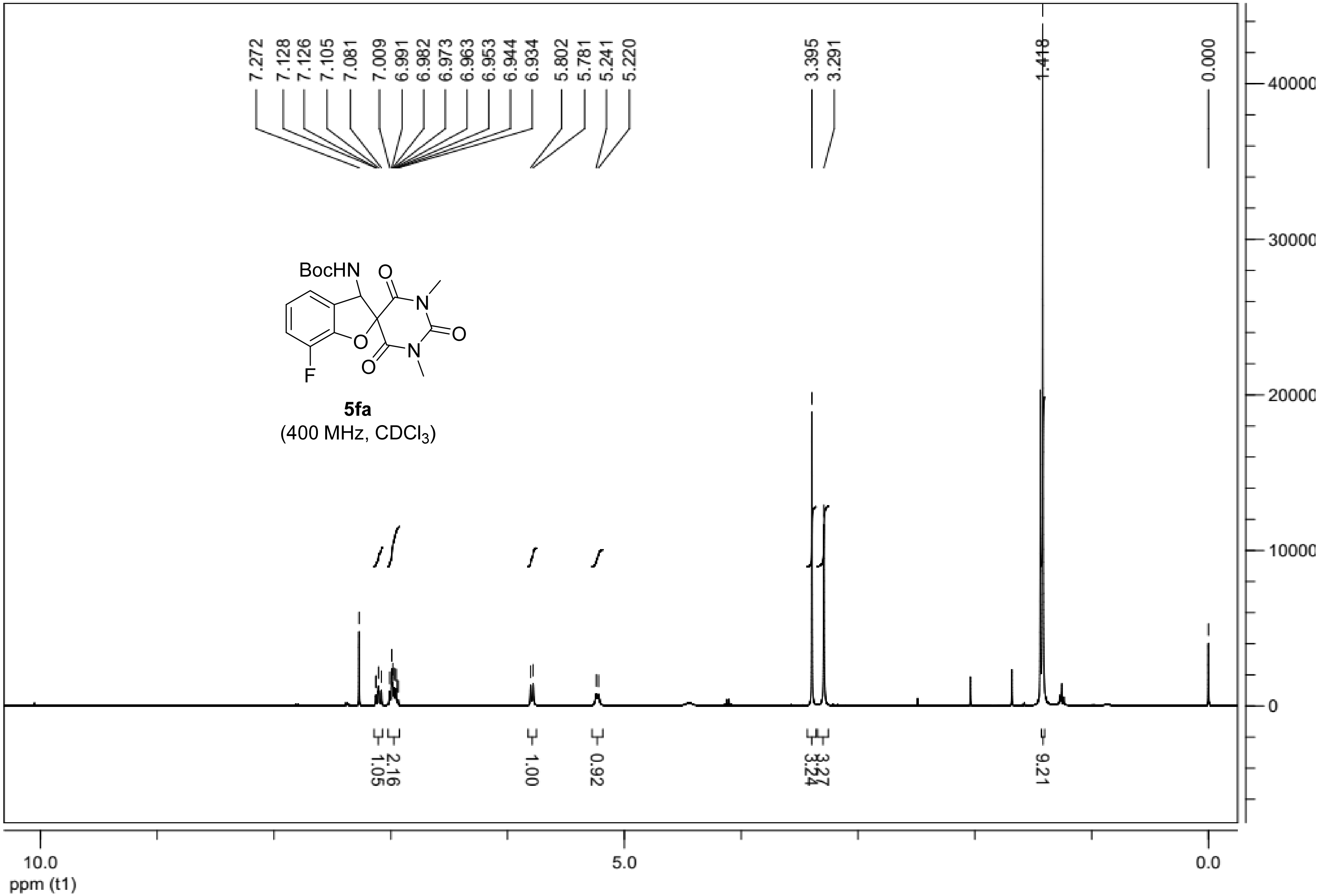
7.272
7.128
7.126
7.105
7.081
7.009
6.991
6.982
6.973
6.963
6.953
6.944
6.934
5.802
5.781
5.241
5.220
3.395
3.291
1.418
0.000
BocHN
O
N
O
N
O
O
F
5fa
(400 MHz, CDCl3)
1.05
2.16
1.00
0.92
3.24
3.27
9.21
40000
30000
20000
10000
0
10.0
5.0
0.0
ppm (t1)

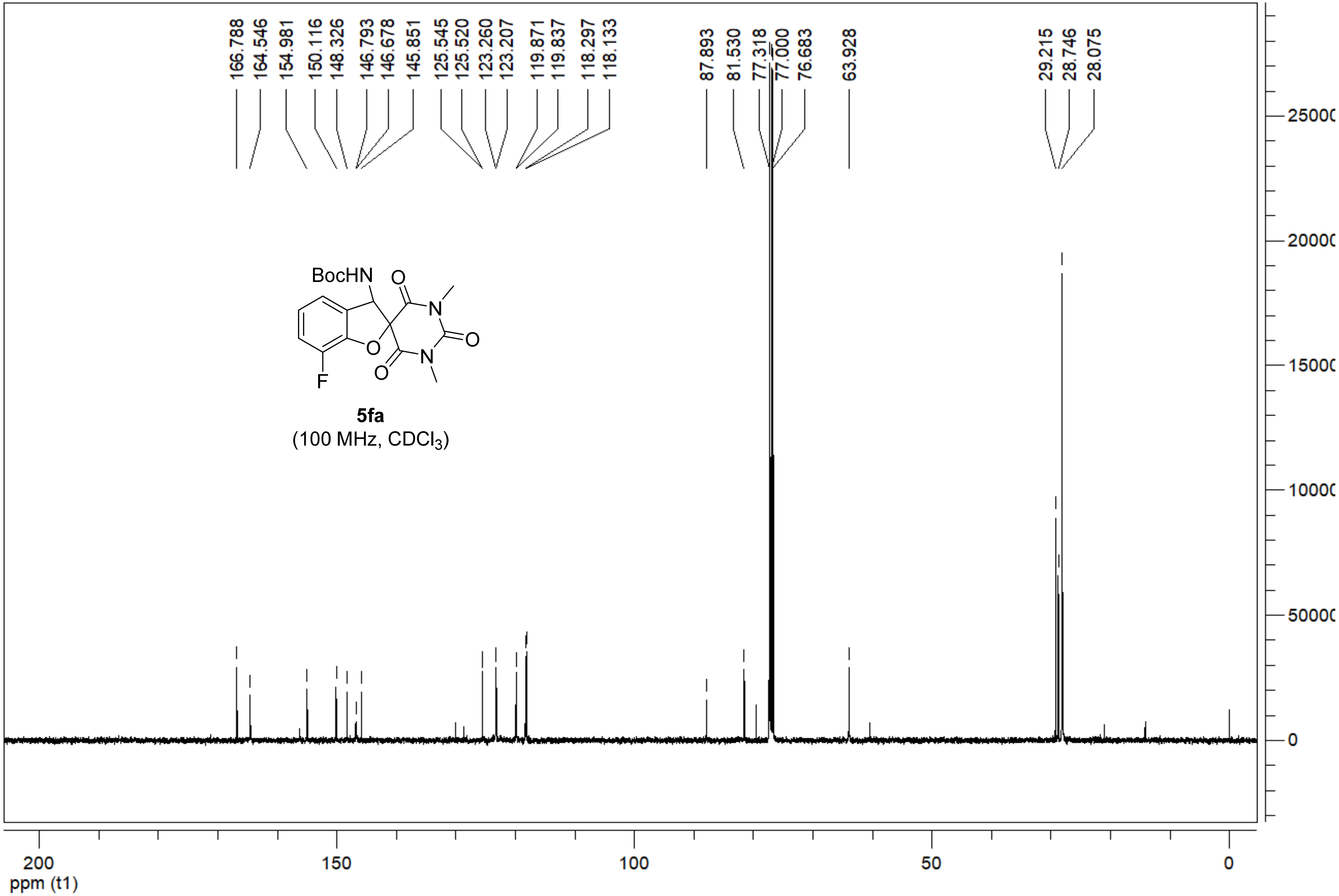
166.788
164.546
154.981
150.116
148.326
146.793
146.678
145.851
125.545
125.520
123.260
123.207
119.871
119.837
118.297
118.133
87.893
81.530
77.318
77.000
76.683
63.928
29.215
28.746
28.075
BocHN
O
N
O
O
N
O
F
5fa
(100 MHz, $CDCl_3$)
200
150
100
50
0
ppm (t1)
25000
20000
15000
10000
50000
0

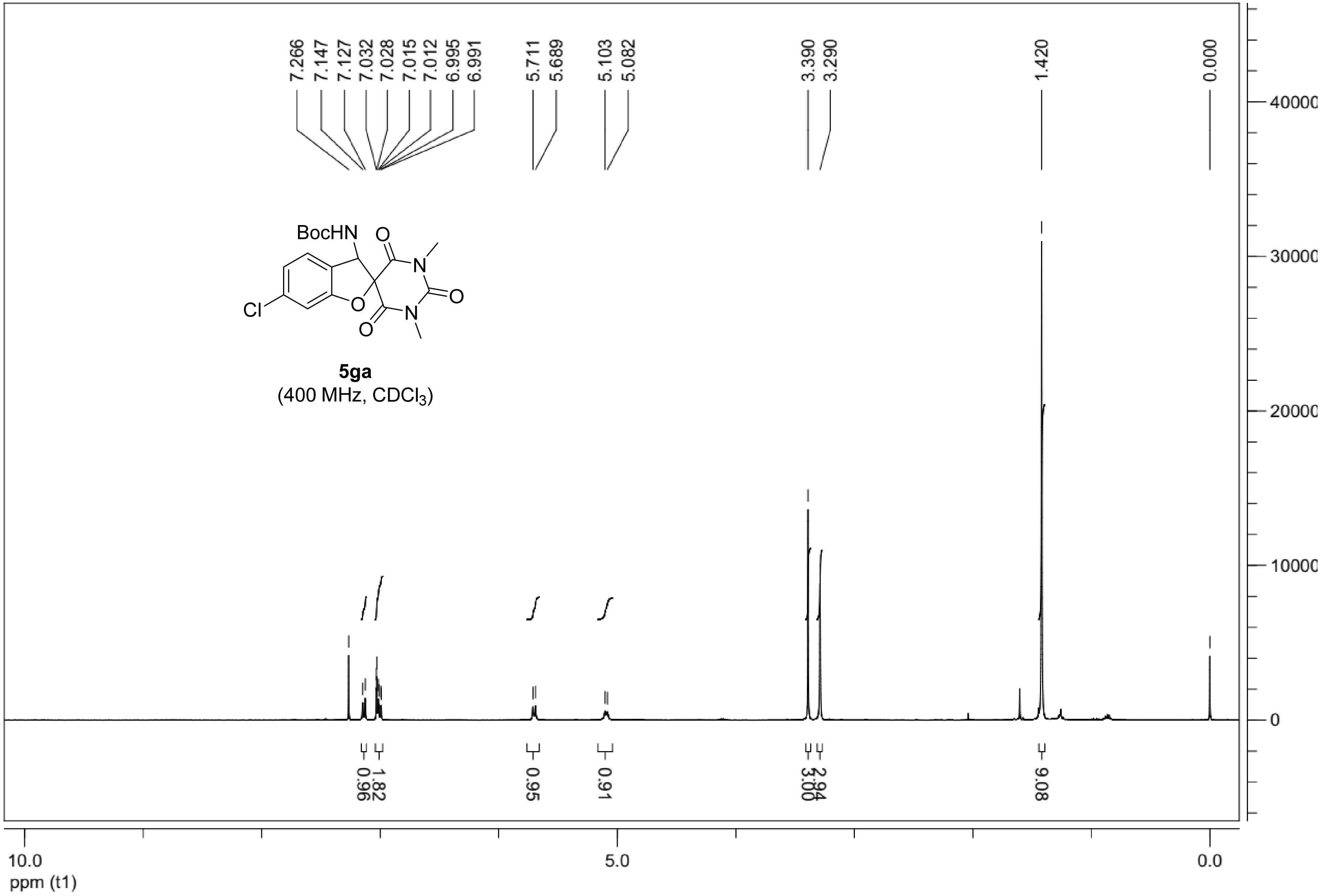
7.266
7.147
7.127
7.032
7.028
7.015
7.012
6.995
6.991
5.711
5.689
5.103
5.082
3.390
3.290
1.420
0.000
BocHN
O
N
O
Cl
O
N
O
5ga
(400 MHz, CDCl3)
40000
30000
20000
10000
0
0.96
1.82
0.95
0.91
3.00
2.94
9.08
10.0
5.0
0.0
ppm (t1)

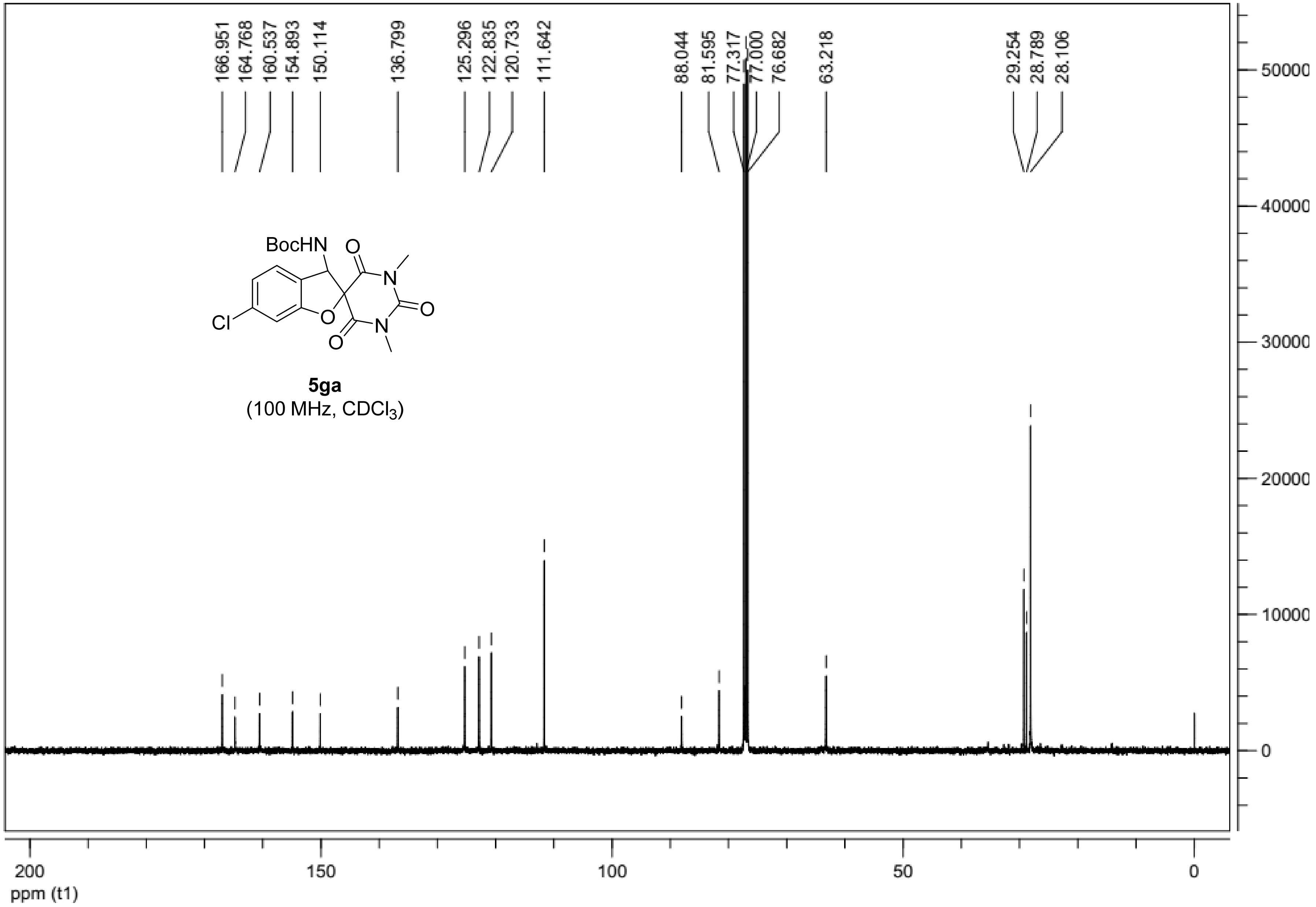
166.951
164.768
160.537
154.893
150.114
136.799
125.296
122.835
120.733
111.642
88.044
81.595
77.317
77.000
76.682
63.218
29.254
28.789
28.106
BocHN
Cl
5ga
(100 MHz, CDCl3)
50000
40000
30000
20000
10000
0
200
150
100
50
0
ppm (t1)

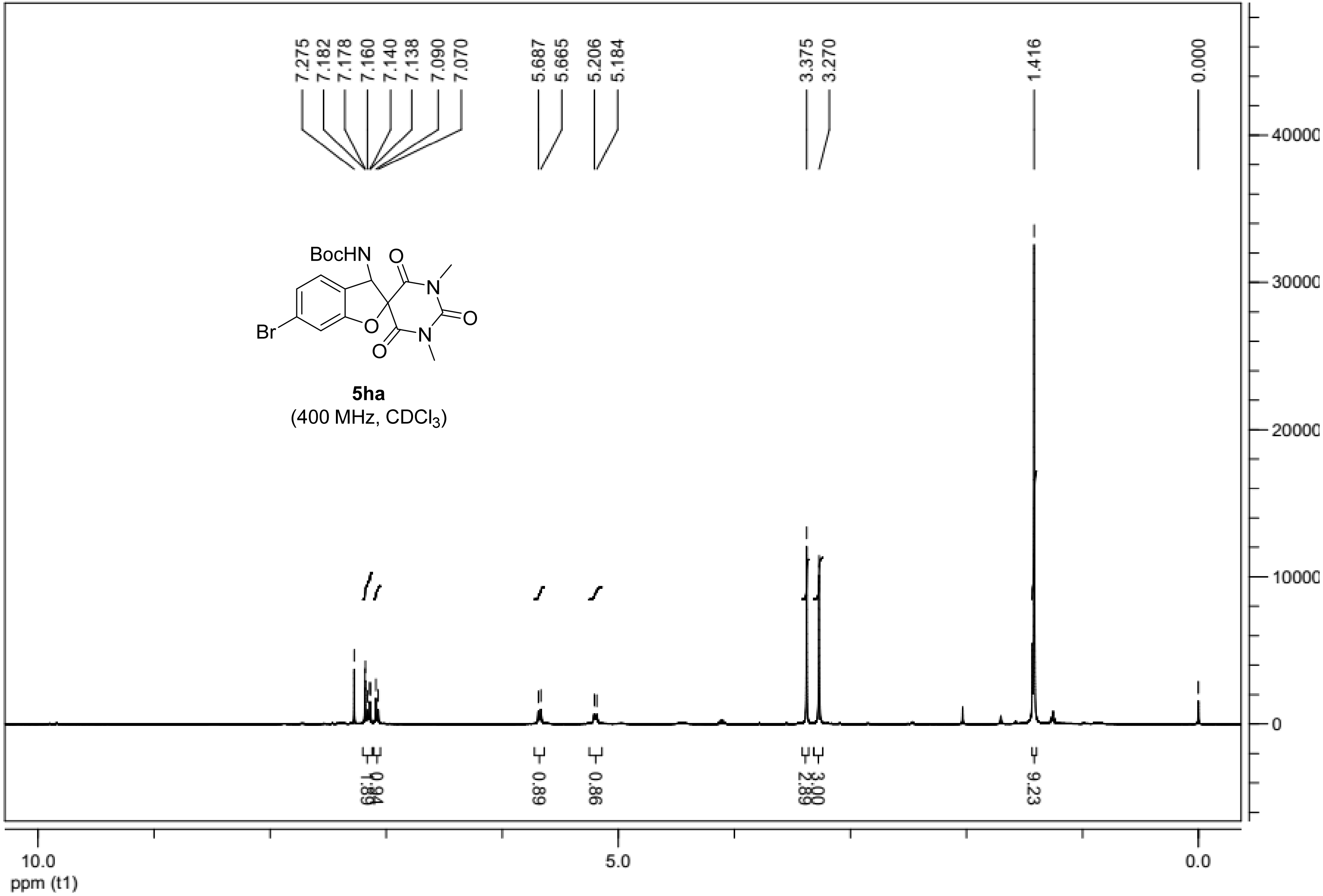
7.275
7.182
7.178
7.160
7.140
7.138
7.090
7.070
5.687
5.665
5.206
5.184
3.375
3.270
1.416
0.000
BocHN
O
N
O
Br
O
O
N
5ha
(400 MHz, CDCl3)
40000
30000
20000
10000
0
1.89
0.94
0.89
0.86
2.89
3.00
9.23
10.0
ppm (t1)
5.0
0.0

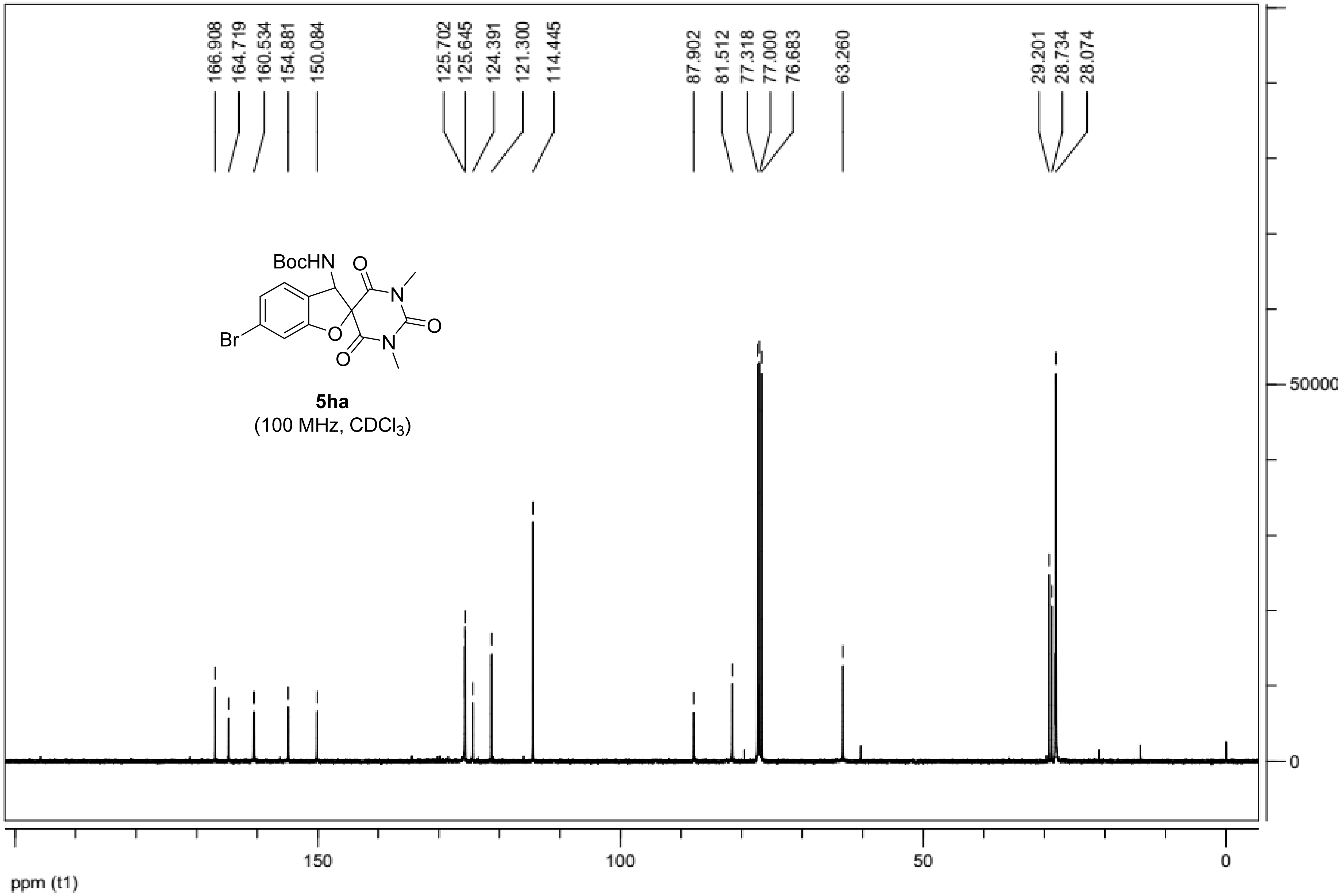

166.908
164.719
160.534
154.881
150.084
125.702
125.645
124.391
121.300
114.445
87.902
81.512
77.318
77.000
76.683
63.260
29.201
28.734
28.074
BocHN
O
N
O
Br
O
N
O
5ha
(100 MHz, CDCl3)
50000
0
150
100
50
0
ppm (t1)
